# Multicellular muscle-tendon bioprinting of mechanically optimized musculoskeletal bioactuators with enhanced force transmission

**DOI:** 10.1126/sciadv.adv2628

**Published:** 2025-07-16

**Authors:** Miriam Filippi, Diana Mock, Judith Fuentes, Mike Y. Michelis, Aiste Balciunaite, Pablo Paniagua, Raoul Hopf, Adina Barteld, Selina Eng, Asia Badolato, Jess Snedeker, Maria Guix, Samuel Sanchez, Robert K. Katzschmann

**Affiliations:** ^1^ Soft Robotics Laboratory, ETH Zurich, Tannenstrasse 3, 8092 Zurich, Switzerland.; ^2^ Institute for Bioengineering of Catalonia (IBEC), Barcelona Institute of Science and Technology (BIST), Baldiri-Reixac 10-12, 08028 Barcelona, Spain.; ^3^ Empa, Swiss Federal Laboratories for Materials Science and Technology, Überlandstrasse 129, 8600 Dübendorf, Switzerland.; ^4^ Department of Biomedical Engineering, University of Basel, Hegenheimermattweg 167b, 4123 Allschwil, Switzerland.; ^5^ Laboratory for Orthopedic Biomechanics, University of Zurich and ETH Zurich, Lengghalde 5, 8008 Zurich, Switzerland.; ^6^ Department of Materials Science and Physical Chemistry, Institute of Theoretical and Computational Chemistry, University of Barcelona, 08028 Barcelona, Spain.; ^7^ Institució Catalana de Recerca i Estudis Avançats (ICREA), Passeig de Lluís Companys 23, 08010 Barcelona, Spain.

## Abstract

Biohybrid actuators leveraging living muscle tissue offer the potential to replicate natural motion for biomedical and robotic applications. However, challenges such as limited force output and inefficient force transfer at tissue interfaces persist. The myotendinous junction, a specialized interface connecting muscle to the tendon, plays a critical role in efficient force transmission for movement. Engineering muscle-tendon units in vitro is essential for replicating native musculoskeletal functions in biohybrid actuators. Here, we present a three-dimensionally bioprinted system integrating skeletal muscle tissue with tendon-mimicking anchors containing fibroblasts, forming a biomimetic interdigitated myotendinous junction. Using computational models, we optimized muscle geometries to enhance deformation and force generation. The engineered system improved mechanical stability, myofiber maturation, and force transmission, generating contractile forces of up to 350 micronewtons over a 3-month period. This work highlights how biomimetic designs and mechanical optimization can advance bioactuator technologies for applications in medicine and robotics.

## Teaser

Creation of mechanically optimized living muscle-tendon units mimics natural force transfer for stable biorobotic systems.

## INTRODUCTION

Soft biohybrid robots combine synthetic and biological materials to replicate the architecture and functionality of biological tissue and the motion of living beings (*1*). When exposed to electrical fields, engineered skeletal muscle tissue (eSMT) in vitro can move as a result of the evoked contraction response from cells. eSMT has therefore been widely used as a controllable actuator that simulates natural muscle movement in dynamic biohybrid machines (*2*–*4*). However, a major limitation of engineered muscle-based actuators is their relatively low force output compared with conventional actuation technologies based on artificial materials, which restricts their applicability and performance (*3*).

Whereas single contractile cellular units (i.e., cardiomyocytes or skeletal muscle myotubes) generate forces of ~10 and 1 μN, respectively (*5*, *6*), their multicellular assemblies can produce up to a few millinewton forces (*3*). Depending on the bioactuator’s size, design, cell type, structural composition, and tissue maturation state, the force output of muscle tissue–based bioactuators varies across the micronewton to millinewton domains. For instance, three-dimensional (3D) ring-shaped bioactuators made of cardiac muscle tissue generated from human induced pluripotent stem cell–derived cardiomyocytes produced a contractile force of 0.9 mN (*7*). eSMT-based modular 3D bioactuators based on optogenetic myoblasts generated up to 300 μN (0.56 kPa) of active tension force in response to optical stimuli (*8*). By linking computational modeling to empirical validation, Pagan-Diaz *et al.* (*9*) augmented the force output of autonomous skeletal muscle biobots from ≈200 μN to ≈1.2 mN to actuate on poly(ethylene glycol) diacrylate (PEGDA) scaffolds with designs that were twofold larger than those shown in prior works. Later, centimeter-scale strip-designed bioactuators based on eSMT achieved 2.0-mN forces upon optimization of tissue alignment and maturation with exercise and weight training systems (*10*). When integrated into optimized biohybrid designs, such bioactuators could eventually produce forces in the range of 10 mN, enabling strength-demanding actions such as weight bearing for pick-and-place tasks (*11*).

While spanning a heterogeneous value range, the force output remains comparatively low to that of traditional actuators, remarking that increasing and preserving the force output of eSMT is of utmost relevance to expand the applicability of biohybrid robots. A key puzzle piece in the development of eSMT is to engineer a force transmission structure, conveying the muscle contraction to the structure of a biohybrid robot.

A major challenge within biohybrid systems is the mechanical mismatch between the soft living tissue and rigid synthetic components, which leads to force dispersion, poor mechanical stability, and limited functionality (*3*). The eSMT-based biohybrid robots depend on either biochemical mechanisms for attaching tissue to a substrate or purely mechanical tethering, where the muscle is stretched around synthetic skeletons and held in tension. These approaches can reduce the efficiency of force transmission from the bioactuator to the underlying substrate or skeleton (*3*) and contrast with the mechanisms found in natural musculoskeletal systems, which use tendons to efficiently transmit forces from the muscle tissue to the bones (*12*, *13*). The interface between the tendon and muscle, also known as the myotendinous junction (MTJ), consists of interdigitating tendon fibers and terminal myocytes, which create finger-like projections that increase the contact area between the tendon and muscle. This interdigitation reduces focal stress by dispersing the energy of muscle contractions (*14*). Replicating such a structure in biohybrid machines can efficiently interface the bioactuator to other rigid, synthetic elements (*15*). In particular, generating a coherent interface that features a stiffness gradient would enhance stability and force transmission.

In muscle tissue engineering and biorobotics, tendons are normally replaced by flexible, synthetic structures such as posts or notches (*11*, *16*). To replicate a muscle-tendon unit (MTU) in vitro, Nomura *et al.* (*17*) developed a modular muscle bioactuator with polydimethylsiloxane (PDMS)-based tendon-like structures that were anchored at each side of the eSMT. Even if this system provided the necessary drag force for myotube alignment and tissue growth, the synthetic, rigid tendons displayed mismatching mechanical properties with the soft tissue, making the interface fragile and prone to rupture. These structures may neither resemble native tissue nor biomimetically affect muscle differentiation and force transmission. Even if force dispersion is crucial for mechanically hybrid systems, to date, no study has focused on optimizing the interface to preserve forces in biohybrid robotic systems.

By enhancing muscle tissue maturation, cell coculture systems are promising for bioactuators with improved contractile performance. In particular, coculturing myoblasts with fibroblasts can improve muscle differentiation through direct cell interactions and facilitate growth factor secretion and extracellular matrix production and remodeling (*18*). Moreover, fibroblasts can serve to build tendon-mimicking structures in engineered MTUs. Merceron *et al.* (*19*) fabricated an MTU composed of living tendon and muscle tissue by bioprinting thermoplastic polyurethane and poly(ε-caprolactone) laden with mouse myoblasts and fibroblasts, respectively. With an elastic muscle-like phase and a stiffer tendon-like phase, this MTU presented similar mechanical properties to those of native MTJs and maintained biocompatibility and expression of MTJ-related genes (*19*). However, no data have been reported on the dynamic functionality of the MTU or the effects of coculture on eSMT maturation, which are crucial aspects for developing heterocellular cell culture models and more adaptive, efficient, and responsive biohybrid systems. Bioprinting of human adipose tissue–derived stem cells enabled the maturation of tendon and muscle tissue in coherent units with different physical interface shapes (*20*). In such a study, including a mixing zone of muscle-tendon cells in the interface induced a high MTJ-associated gene expression, suggesting a promising design strategy.

Here, we present a 3D-bioprinted MTU composed of a coculture of C2C12 myoblasts and NIH/3T3 fibroblasts, which features high interface biomimicry and stability, as well as enhanced functional actuation performance. After optimizing muscle and tendon bioinks for extrusion-based bioprinting and eSMT maturation, we realized an MTU with a myotendinous interface that combined layers of bioink mixing zones with a layer of interdigitated zones. This design improved the stability of the myotendinous interface and supported the maturation of the MTJ, effectively mimicking the architecture of the native MTJ. Then, we characterized the eSMT formation and actuation performance in response to the presence of fibroblasts and different bioactuator designs. Moreover, we demonstrate that coculturing two cell types stabilizes the architectural compartmentalization of the system in active and passive components (corresponding to muscle and fibroblast-seeded areas, respectively), by limiting muscle cell invasion of the anchor structures. The MTU-based actuators exhibited long-term contractility, generating forces up to 350 μN with responsiveness maintained over 3 months, thus outperforming similar systems reported in the literature (*21*, *22*).

## RESULTS

### Bioprinting of MTUs with structural biomimicry

**Fig. 1.**
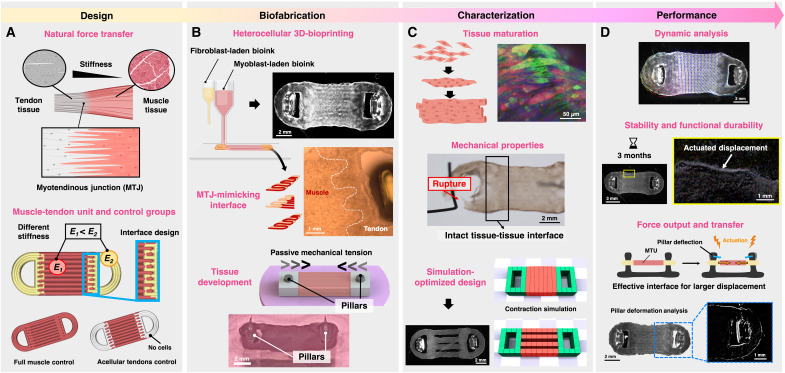
Study overview of engineered MTU for efficient bioactuation. (**A**) Schematic representation of the MTJ’s anatomy as the target of biofabrication (top) and the fabrication process of the muscle-tendon unit (MTU), comprising an eSMT with two tendon-mimicking tissues as anchoring sites in a robotic system. Created in BioRender. M. Filippi (2025) https://BioRender.com/5893ycx. (**B**) To mimic native MTJs, fibroblast- and muscle cell–laden bioinks are coprinted with an interdigitated design using extrusion-based multimaterial 3D bioprinting to form a multilayered structure (top). The construct matures into a tissue under passive mechanical tension (bottom). Created in BioRender. M. Filippi (2025) https://BioRender.com/87dompp. (**C**) The MTU is characterized by its biological and mechanical properties (top) and is further optimized via simulation to maximize surface area and enhance the production of larger active eSMTs (bottom). Created in BioRender. M. Filippi (2025) https://BioRender.com/3jetq93. (**D**) The MTU undergoes testing to assess its dynamic bioactuation response, stability, long-term contractile performance, force output, and force transfer efficiency when interacting with synthetic components. Created in BioRender. M. Filippi (2025) https://BioRender.com/zt7ua98.

We built the MTU and replicated the interdigitating architecture of the natural MTJ (Fig. 1A) using multimaterial extrusion-based 3D bioprinting. The chosen printing approach and the use of optimized bioinks with common polymeric components allowed us to interconnect the bioinks at a fine resolution (approximately 350 μm) and form a coherent phase between the muscle- and tendon-like tissue (Fig. 1B). The MTU consisted of a triple-layered tissue with a nominal size of 6 mm by 3 mm by 15 mm with two tendon-like anchors at the extremities of the eSMT.

We created a mechanical gradient promoting force transfer in the biohybrid MTU by coprinting two hydrogel bioinks with different mechanical properties, seeded with myoblasts and fibroblasts (Fig. 1, A and B). To assess the architectural and biological effects of the cellular coculture on the bioactuator, the MTUs were compared to control constructs realized from sole myoblast-laden bioink, or lacking fibroblasts in the anchors (“acellular tendons” condition) (Fig. 1A, bottom). The MTU constructs were directly printed on tissue maturation templates with pillars to generate passive mechanical tension for proper tissue development (Fig. 1B, bottom). After characterizing the biomechanical properties of the MTUs and optimizing their performance through dynamic simulation (Fig. 1C), we demonstrated a biohybrid system exhibiting long-term stability, functional durability, and improved force production as well as efficient force transfer (Fig. 1D).

**Fig. 2.**
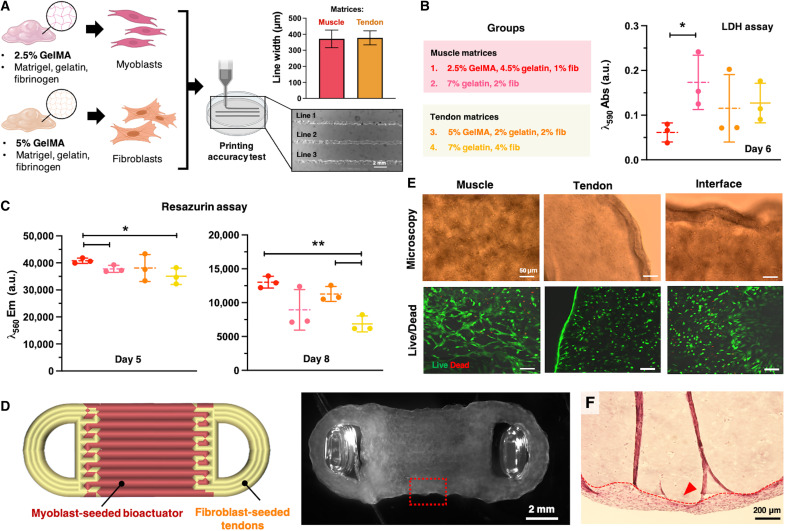
3D bioprinting of the muscle-tendon units. (**A**) Formulation of bioinks that were optimized for bioprinting of skeletal muscle tissue and tendon-mimicking structures with myoblasts and fibroblasts, respectively. Printability assessment via measurement of the printed line width. Created in BioRender. M. Filippi (2025) https://BioRender.com/zt7ua98. (**B**) Absorbance of bioinks with different matrix formulations reporting on LDH release after 6 days of culture. (**C**) Fluorescence emission of bioinks with different matrix formulations of matrix reporting on cell metabolic activity measured via Resazurin assay at culture days 5 and 8 (*n* = 3). In (B), *P* values are as follows: 0.039 (groups 1 and 2), 0.358 (2 and 3), 0.827 (3 and 4), 0.0806 (1 and 4), 0.299 (1 and 3), and 0.346 (2 and 4). In the left panel of (C), *P* values are as follows: 0.033 (1 and 2), 0.918 (2 and 3), 0.413 (3 and 4), 0.0343 (1 and 4), 0.413 (1 and 3), and 0.220 (2 and 4). In the right panel of (C), *P* values are as follows: 0.085 (1 and 2), 0.272 (2 and 3), 0.009 (3 and 4), 0.001 (1 and 4), 0.099 (1 and 3), and 0.328 (2 and 4). (**D**) CAD of MTU construct (red, muscle bioink; orange, tendon bioink) and stereomicroscopic image of the bioprinted construct (immediately after bioprinting). (**E**) Microscopic (top) and confocal (bottom) imaging of MTU obtained from optimized bioinks and stained for Live/Dead imaging assay, 2 days after bioprinting. (**F**) H&E staining of the central muscle structure [red, dashed box in (D)] showing the internal cell-lacking matrix and the eSMT layer growing at the surface (red arrowhead). The red dashed line highlights the border between the acellular matrix and the superficial tissue layer. Statistical significance is expressed as follows: *P < 0.05 and **P < 0.01.

We prepared the MTUs by developing two bioink formulations featuring suitable mechanical properties for bioprinting, cell spreading, tissue development, and stable coassembly (Fig. 2A and figs. S1 to S4). We formulated different hydrogel compositions based on a mixture of gelatin methacrylate (GelMA), Matrigel, and fibrinogen with variable relative fractions of gelatin as a modulator of viscosity (Fig. 2A). Specifically, the muscle-based bioink consisted of 2.5% (w/v) GelMA, 4.5% (w/v) gelatin, 1% (w/v) fibrinogen, and 20% (v/v) Matrigel. To create an MTU with distinct stiffness gradients, where the tendon is stiffer than the muscle, we developed a tendon-based bioink with higher concentrations of GelMA and fibrinogen, comprising 5% (w/v) GelMA, 2% (w/v) gelatin, 2% (w/v) fibrinogen, and 20% (v/v) Matrigel. The formulations were printed with similar space resolution, as assessed by measuring the printed ink line width (371.5 ± 86.5 and 377.4 ± 80.6 μm for muscle and tendon matrices, respectively; Fig. 2A and table S1). Then, to assess the biocompatibility of the optimized matrices, we added the cells to formulate the muscle and tendon bioinks, and printed ring-shaped 3D constructs, on which we evaluated cell cytotoxicity and viability for each bioink individually, via lactate dehydrogenase (LDH), Resazurin, and Live/Dead staining assays.

In bioinks, GelMA acts as a reinforcement to maintain construct integrity over time while preserving bioactivity and supporting cell adhesion, making it a promising strategy for more durable tissue constructs (*23*). As it can provide covalent cross-linking, reduce shrinkage, and improve mechanical resilience, GelMA was included in our bioink formulations to enhance stability. To evaluate the impact of GelMA addition on the final construct, the optimized matrices were compared with cell-laden constructs printed using bioinks without GelMA, that is, formulations containing either 7% (w/v) gelatin and 2% (w/v) fibrinogen or 7% (w/v) gelatin and 4% (w/v) fibrinogen, both with 20% (v/v) Matrigel.

Six days after biofabrication, cells in the tendon matrix-based rings displayed LDH release values that were similar to those measured for the samples without GelMA, whereas the LDH release in the optimized muscle matrix-based rings was significantly lower than in the GelMA-free samples (Fig. 2B). The Resazurin assay showed that 5 days after biofabrication, myoblasts were significantly more metabolically active in the optimized muscle matrix than in the related GelMA-free muscle rings, whereas fibroblasts in the tendon matrices displayed similar metabolic activity levels (Fig. 2C). A similar trend was observed on day 8, with fibroblasts also showing a more active metabolism when included in GelMA-enriched inks. These results confirm that, even in the presence of GelMA, the selected bioink formulations maintain a high level of cell viability and preserve cellular metabolic activity for several days after biofabrication.

We used the optimized matrices to bioprint our MTU structure accounting for a central, compact muscle tissue unit flanked by two anchors that mimic tendons for connection to rigid segments (Fig. 2D). As shown by microscopic observation and confocal imaging with Live/Dead staining, 2 days after biofabrication, the cells in the muscle and tendon-like components and interface of the MTU were viable and displayed elongated and spindle shapes, respectively, suggesting that the cell populations could grow on the two different substrates (Fig. 2E). A thick tissue layer formed on the surface of the construct (Fig. 2F), while a lack of nutrients and oxygen prevented cell formation at depths greater than 150 to 200 μm, as typically observed in hydrogel constructs lacking perfusable structures (*24*, *25*).

**Fig. 3.**
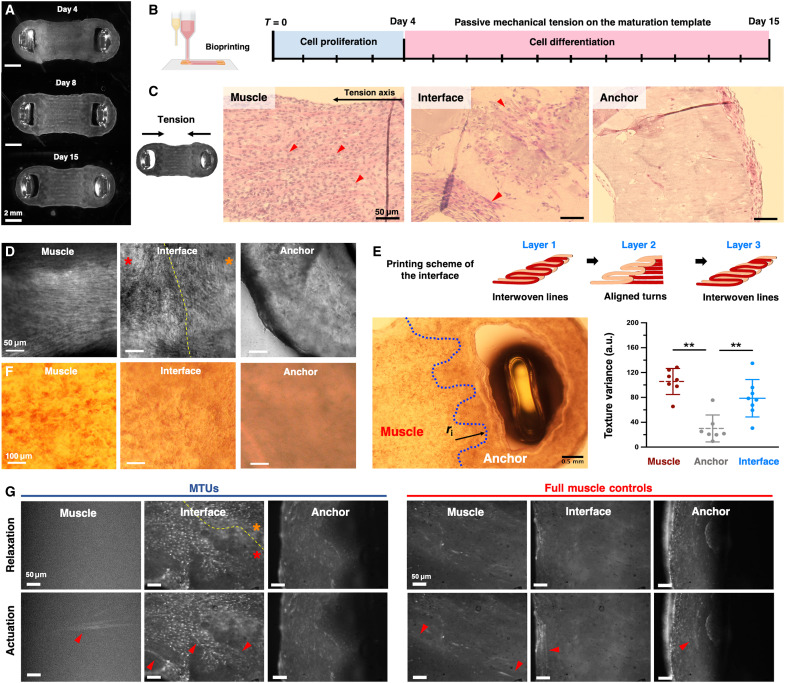
Stability and tissue architecture during tissue development. (**A**) Stereoscopic microscopy of the MTU at different time points of the tissue development process. (**B**) Culture conditions for muscle tissue maturation, accounting for culture in growth medium for 4 days and myogenic differentiation medium for 11 days after mounting on the tissue maturation template under mechanical tension. H&E staining (**C**) and confocal imaging (**D**) of the muscle tissue, tendon-muscle interface, and tendon structure on day 15. In (C), red arrowheads indicate myotubes. In (D), the yellow dashed line represents the interface border between the two tissues, as determined by manual positioning of the microscope. Orange and red asterisks indicate the muscle and tendon sides, respectively. Created in BioRender. M. Filippi (2025) https://BioRender.com/87dompp. (**E**) Scheme of the printing sequence to generate the interface and microscopic picture of the muscle-tendon interface (culture day 15), revealing the interdigitated architecture joining the muscle and fibroblast-seeded tissues and texture variance of microscopic pictures quantified in the muscle, interface, and anchor areas of the constructs (*n* = 8). The blue dashed line indicates the interdigitated pattern of the interface, and the black arrow indicates the radius of the invagination (*r*_i_). *P* values are as follows: 0.002 (muscle versus tendon), 0.002 (tendon versus interface), and 0.461 (muscle versus interface). (**F**) Representative pictures of the different MTU areas showing a different matrix texture. (**G**) Calcium imaging of the different regions of the MTU and controls. Red arrows indicate activated myotubes, and yellow dashed lines indicate the interface border between the two tissues, as determined by the manual positioning of the microscope. Orange and red asterisks indicate the muscle and tendon sides, respectively. Statistical significance is expressed as follows: **P < 0.01.

As multimaterial constructs that are exposed to mechanical stress for proper tissue maturation often have instability at the material interface, we monitored the maturation of the constructs via macro- and microscopic observation over time (Fig. 3). The MTU constructs remained intact during the whole tissue maturation process including the cell proliferation and differentiation phases (Fig. 3, A and B). Including GelMA in the bioinks (Fig. 2) and refining the tendon structures for a more compliant design resulted in enhanced stability of the bioassembly (figs. S1 to S4). The bioinks were optimized for high printing fidelity and stability, ensuring the formation of mature constructs that retained their designed features over weeks of culture. During this period, MTU constructs and controls exhibited similar size reductions, with final mass retention around 90 to 95% of the initial value (fig. S4). This indicates that material degradation was minimal, and hydrogel shrinkage along with tissue remodeling led to only mild deformations of the original structure.

After 15 days of culture, hematoxylin and eosin (H&E) staining on tissue sections (Fig. 3C) and microscopic imaging on whole constructs (Fig. 3D) revealed large multinucleated myotubes forming in the muscle area of the MTU [left panel in Fig. 3 (C and D)]. Cell nuclei were clustered and myotubes unidirectionally aligned parallel to the longitudinal axis of the construct, contributing to the anisotropic architecture expected for proper eSMT development (Fig. 3, C and D, left). Upon manual positioning of the microscope camera in the interface area, we inspected the connection area between the two tissues. The interface area contained matrix areas with cells with different morphologies and densities, including myotubes (Fig. 3, C and D). The tendon structures were covered with a tissue of ∼50 μm thickness (Fig. 3C, right), in which myotubes were only sporadically observed (Fig. 3D, right). In contrast, in the constructs composed of sole muscle bioink or featuring anchors realized with the acellular matrix, a large number of myotubes were observed in the anchor area (figs. S5 to S7).

We connected the two tissues by printing layers with different arrangements of the two bioinks, in which the bioink deposition of interwoven lines or aligned turns alternated (Fig. 3E and fig. S8). Under microscopic imaging, the two tissues displayed a different texture reflecting the diverse matrix composition (Fig. 3, E and F), which facilitated the visual inspection of the constructs to evaluate the architecture of the multimaterial assembly. The microscopic observation of the MTUs revealed an intertissue interface pattern that featured interdigitations. The interdigitation radius (i.e., depth of the turns within the interlocking zones, as shown by the black arrow in Fig. 3E) was approximately 0.8 mm. Thus, assuming an average construct length of 15 mm, the two tissues interpenetrated for approximately 5% of the total MTU length. Such an interlocking arrangement guaranteed the cohesion between the two printed materials, suggesting that combining interdigitated design with bioink mixing zones at the muscle and tendon interface resulted in the successful integration of the forming tissues.

As shown via calcium imaging, action potentials were primarily detected in the muscle area of the MTU, while only sporadic muscle activity was observed in the tendon region (Fig. 3G). Control constructs displayed functional muscle activity in the anchor areas. These data suggest that fibroblasts in the MTU can hinder the migration of myoblasts from the central part of the construct (bioactuator area) to the anchors, and limit the expansion of muscle tissue in the tendon regions.

### Simulation-driven design optimization

Owing to the limited oxygen and nutrient supply, the cells at the core of centimeter-scale 3D cell culture models typically become quiescent or die (*25*, *26*). This results in constructs covered with layers of viable cells but lacking cells in the internal regions. Thus, we designed the MTUs to augment the surface available and form active, contractile tissue for higher force output.

**Fig. 4.**
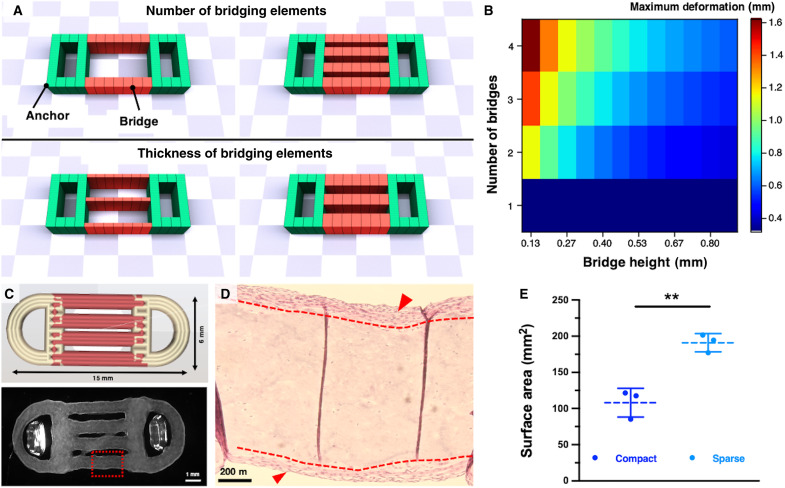
Bioactuator’s design consideration for optimal cell viability and maximized contractile tissue area. (**A**) Simulation of the actuation of the MTU realized with different configurations of the sparse design, varying the number and the thickness of the bridge elements. (**B**) Maximum deformation of the MTUs’ simulated motion in response to varying numbers and sizes of the bridge-like elements. (**C**) CAD of MTU constructs with a sparse, four-bridged design (red, muscle bioink; orange, anchor bioink) and stereotactic microscopic image of the bioprinted construct (culture day 15). (**D**) H&E staining of the sparse MTU (culture day 15) showing the inner muscle structure [red, dashed box in the bottom panel of (C)] with a bridged design (i.e., “sparse” design) and eSMT layers at the top and bottom surface of a bridge segment (red arrowheads). (**E**) Surface area of the muscle tissue within compact and sparse constructs, supposed to actively contribute to force generation, as estimated via image analysis (*n* = 3). *P* value*:* 0.004. Statistical significance is expressed as follows: **P < 0.01.

To anticipate the actuation performance of the MTU as a function of the realized design, we simulated the quasistatic contractile behavior of MTU constructs. The simulated MTU designs featured a sparse, bridged muscle structure (Fig. 4A). As our actuation model sets a fixed strain on the muscle elements, they always contract the same relative amount of their total length. Given that the length of the constructs would only monotonically augment the maximum deformation and was defined by the previously printed constructs, we did not investigate the dynamic effects of its variation. In contrast, we performed a parameter sweep over the number of bridges and the width of the bridges to observe how these different geometries affect the final amount of deformation. Increasing the number of central muscle tissue segments (i.e., “bridges”) and decreasing their diameter resulted in an overall movement increase, quantified by the maximum deformation of the muscle (Fig. 4B). These results in the parameter sweep show that a maximum surface area of contractile tissue will result in the most deformation.

In the end, we printed sparse MTUs with four bridges as a design that was optimized for maximal movement (Fig. 4C). Even if the surface tissue matured successfully in both the compact and sparse MTU designs, histological analysis demonstrated that no tissue formed in the internal region of the MTU (Figs. 2F and 4D). In the sparse design, the surface area available for tissue formation was estimated to be approximately 77% larger than the one in the compact design (Fig. 4E).

It should be noted that although the constructs were accurately printed with initially separated segments, during culture, in some cases, segments merged at contact points due to their close positioning and movement within the medium (fig. S9). Segment fusion primarily occurred in the central bridges, likely because of their constrained position, while external segments had greater freedom of movement.

To evaluate whether design principles could effectively improve MTU performance, the optimized construct design (referred to as “sparse MTU”) was incorporated into further experiments and compared with both the compact MTU and control conditions. This comparison aimed to assess the impact of the sparse design on the mechanical and functional properties of the MTUs.

### Characterization of the tissue maturation

**Fig. 5.**
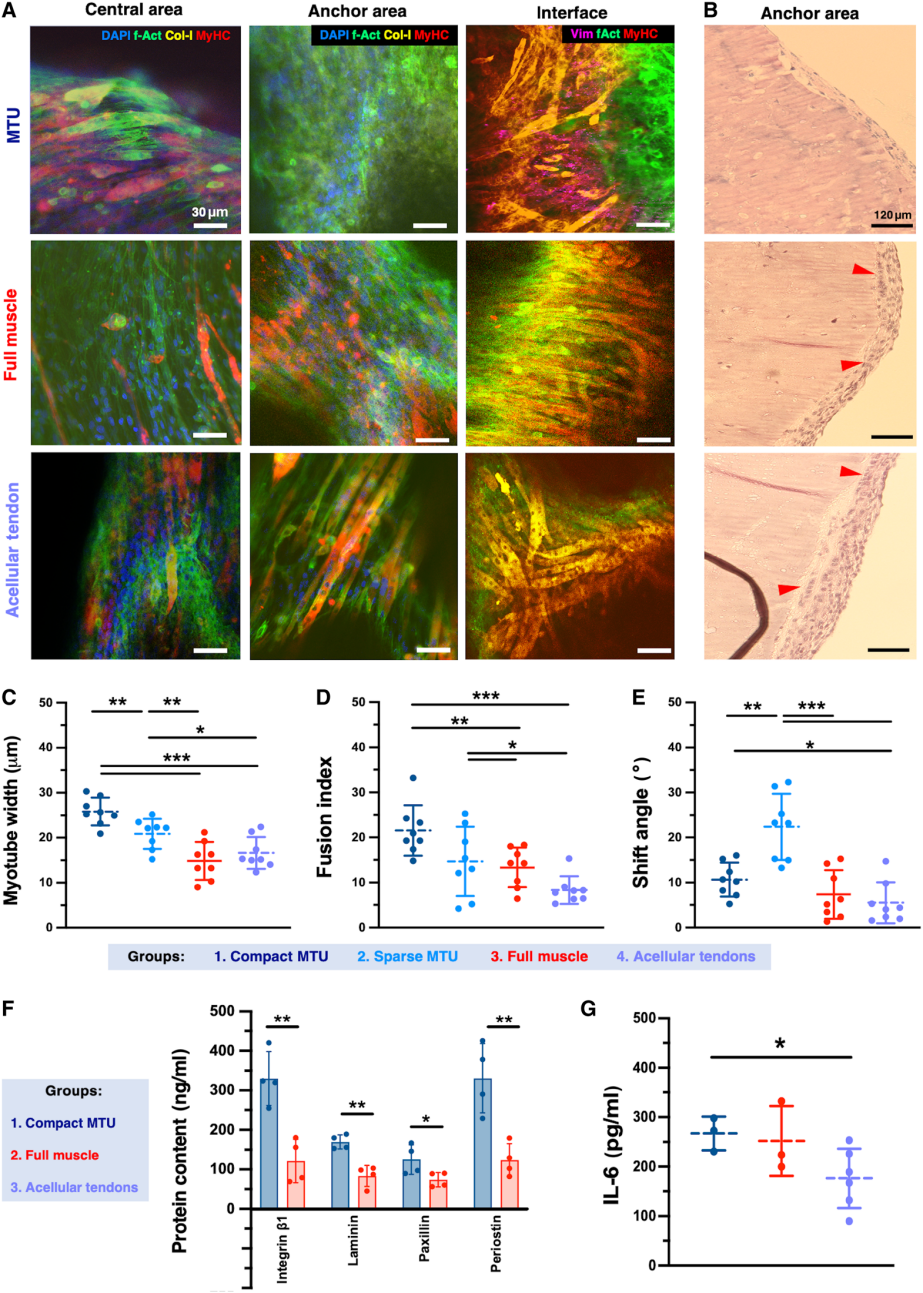
Effects of the coculture on the tissue maturation process. (**A**) Confocal imaging of compact MTUs and controls with sole muscle bioink or acellular tendons, on the central area, anchor, and interface (culture day 15). Nuclei, F-actin, MyHC, and collagen I/vimentin are shown in blue, green, red, and yellow, respectively. (**B**) H&E staining of the anchor areas of MTUs (culture day 15). Red arrowheads indicate aligned nuclei, suggesting myotubes. Myotube width (**C**) and fusion index (**D**) were calculated from myotubes visualized in the muscle tissue areas of MTU and control constructs (culture day 15) by F-actin staining (*n* = 8). Fusion index is calculated as the number of nuclei inside MyHC^+^ myotubes divided by the total number of nuclei present in a field of view (*n* = 8). In (C), *P* values are as follows: 0.008 (groups 1 and 2), 0.007 (2 and 3), 0.378 (3 and 4), 0.027 (2 and 4), 7.231 × 10^−5^ (1 and 4), and 3.546 × 10^−5^ (2 and 3). In (D), *P* values are as follows: 0.061 (1 and 2), 0.674 (2 and 3), 0.019 (3 and 4), 0.048 (2 and 4), 4.218 × 10^−5^ (1 and 4), and 0.006 × 10^−5^ (1 and 3). (**E**) Myotube alignment is expressed as a shift angle between the long axis of the myotube and the *x*-axis direction (*n* = 8). In (E), *P* values are as follows: 0.001 (1 and 2), 3.7 × 10^−4^ (2 and 3), 0.470 (3 and 4), 7.599 × 10^−5^ (2 and 4), 0.028 (1 and 4), and 0.181 (2 and 3). (**F**) MTJ marker proteins were quantified via ELISA of the tissue homogenates (*n* = 4). In (F), *P* values are as follows: 0.003, 0.002, 0.049, and 0.005 for integrin β1, laminin, paxillin, and periostin, respectively. (**G**) Release of IL-6 as measured in the supernatant of constructs via ELISA assay (culture day 11; *n* ≥ 3). In (G), *P* values are as follows: 0.714 (1 and 3), 0.049 (1 and 4), and 0.158 (3 and 4). Statistical significance is expressed as follows: *P < 0.05, **P < 0.01, and ***P < 0.001.

To investigate the benefits of using living tendon components in the bioactuators, the tissue development performance of the MTUs was compared to full muscle constructs and constructs with acellular tendons to understand how the myoblast-fibroblast coculture affects tissue development. Whole-mount confocal imaging of the MTUs and H&E staining of their sections revealed myotubes in the central area of all construct types, indicating successful eSMT maturation (Fig. 5, A and B, and figs. S5 and S6).

Myotubes expressing the muscle maturity marker Myosin Heavy Chain (MyHC) were also found in the anchor area of acellular tendon constructs [Fig. 5, A (bottom right) and B (bottom)], indicating that myoblasts migrated from the central area of the constructs to the anchors. In contrast, in the anchors of the MTU constructs, fewer or no myotubes were observed, suggesting that fibroblasts hindered myoblast migration to the anchor area (Fig. 5A, top). The interface area of the MTUs displayed distinct compartments with cells with different morphologies, while in control constructs, myotubes extended over the interface. In the MTUs, vimentin, an intermediate filament protein that is primarily expressed in fibroblasts, was detected, helping differentiate the two cell types at the interface (Fig. 5A and fig. S7). In coculture systems, fibroblasts often contribute to extracellular matrix deposition, while myoblasts rely more on the extracellular matrix for support and signaling (*27*). Coherently, we found that fibroblasts have a significantly higher collagen secretion capacity than myoblasts (Fig. 5A and fig. S7).

To gain more quantitative insights into the tissue development outcome, we performed a morphological and biochemical analysis of the tissue. To maximize the bioactuator’s performance, the eSMT must achieve maturity across the myogenic differentiation process and create aligned fiber arrays to properly combine the output force of each fiber during their collective contraction. Thus, we assessed the ability of myoblasts to fuse and form large, mature myofibers, as well as the myotubes’ orientation.

In compact MTUs, the myotube width was measured at 25.8 ± 3.1 μm. These myotubes were larger than those observed in all other construct types, with a width increase of 22.1, 42.3, and 35.3% compared to sparse MTUs, full muscle, and acellular tendon constructs, respectively (Fig. 5C). We then calculated the fusion index, as the percentage of nuclei located within MyHC^+^ multinucleated myotubes relative to the total number of nuclei in the sample (Fig. 5D). This parameter is commonly used to quantify cell fusion, which reflects enhanced differentiation and maturation of the engineered muscle tissue. We observed that myotubes in both compact and sparse MTUs had a significantly higher fusion index (21.5 ± 5.6) than full muscle (13.3 ± 4.4) and acellular tendon (8.3 ± 3.1) constructs.

We then calculated the myotubes’ shift angle, which reports on the misalignment of myotubes from a predefined direction, and reflects the casualty of the myofiber organization. Although no significant difference was observed in the fusion index between the compact and sparse MTUs, the shift angle of myotubes was significantly lower in compact MTUs than in sparse MTUs. This indicates that myotubes in compact MTUs had better alignment along the tension axis than those formed in sparse MTUs (Fig. 5E), possibly due to the loosening of the mechanical tensioning on the central segments. Overall, these results indicated that the presence of fibroblasts positively affected the maturation of the eSMT and that the design choice can also affect the process of tissue development from a mechanical perspective.

To identify the extent of MTJ development, we compared the protein content patterns of our bioprinted MTUs with muscle-only controls (Fig. 5F). Enzyme-linked immunosorbent assay (ELISA) protein quantification on the tissue homogenates revealed that MTJ-associated proteins (i.e., paxillin, integrin β1, laminin, and periostin) were more abundant in the MTUs than in controls. Periostin is a crucial extracellular matrix protein expressed at the MTJ, playing a crucial role in the interface between muscle fibers and tendons (*28*). Augmented periostin levels colocalizing with increased integrin and laminin production are likely to relate to MTJ stabilization. These findings are in line with the gene expression analysis of other constructs fabricated from the coculture of fibroblasts and myoblasts, in which the focal adhesion markers were found up-regulated at the MTJ region (*19*).

To verify the tissue maturity of MTU and controls, the cell culture medium was sampled at 11 days of culture and analyzed for the content of interleukin-6 (IL-6), a myokine involved in the early stages of myogenesis and tissue remodeling, and secreted by muscle cells in response to mechanical loading and differentiation cues (*29*–*33*). IL-6 serves as a strong molecular indicator of eSMT maturity at a late stage of tissue development. During skeletal muscle development, IL-6 plays a crucial role in satellite cell activation, myoblast proliferation, and subsequent differentiation into mature myotubes (*30*, *32*). In addition to being involved in extracellular matrix remodeling and cross-talk between myoblasts and fibroblasts (*34*–*36*), IL-6 is released in response to muscle contractions and metabolic demands, serving as a marker of muscle activity (*37*–*39*). Its presence on day 11 may reflect the onset of functional contractile properties in our MTUs.

As compared to acellular tendon controls, after 11 days of tissue development, the MTU constructs released a higher amount of IL-6. By this time point, an increase in IL-6 suggests that the biofabricated MTU is undergoing active maturation (Fig. 5G). The amount of released cytokine was not significantly different from the one measured in the full muscle constructs, in which, however, the initial muscle cell seeding density was higher. As native muscle tissue exhibits transient IL-6 expression during early development and regeneration following injury (*29*, *40*, *41*), the observation that the MTU mimics this pattern a few days after biofabrication suggests that the tissue development process successfully recapitulates physiological muscle maturation cues. Overall, these results suggest that coculturing fibroblasts with myoblasts enhances the myogenic differentiation process while preserving cell compartmentalization within our heterocellular system.

### Assessment of the contractility and dynamic properties

**Fig. 6.**
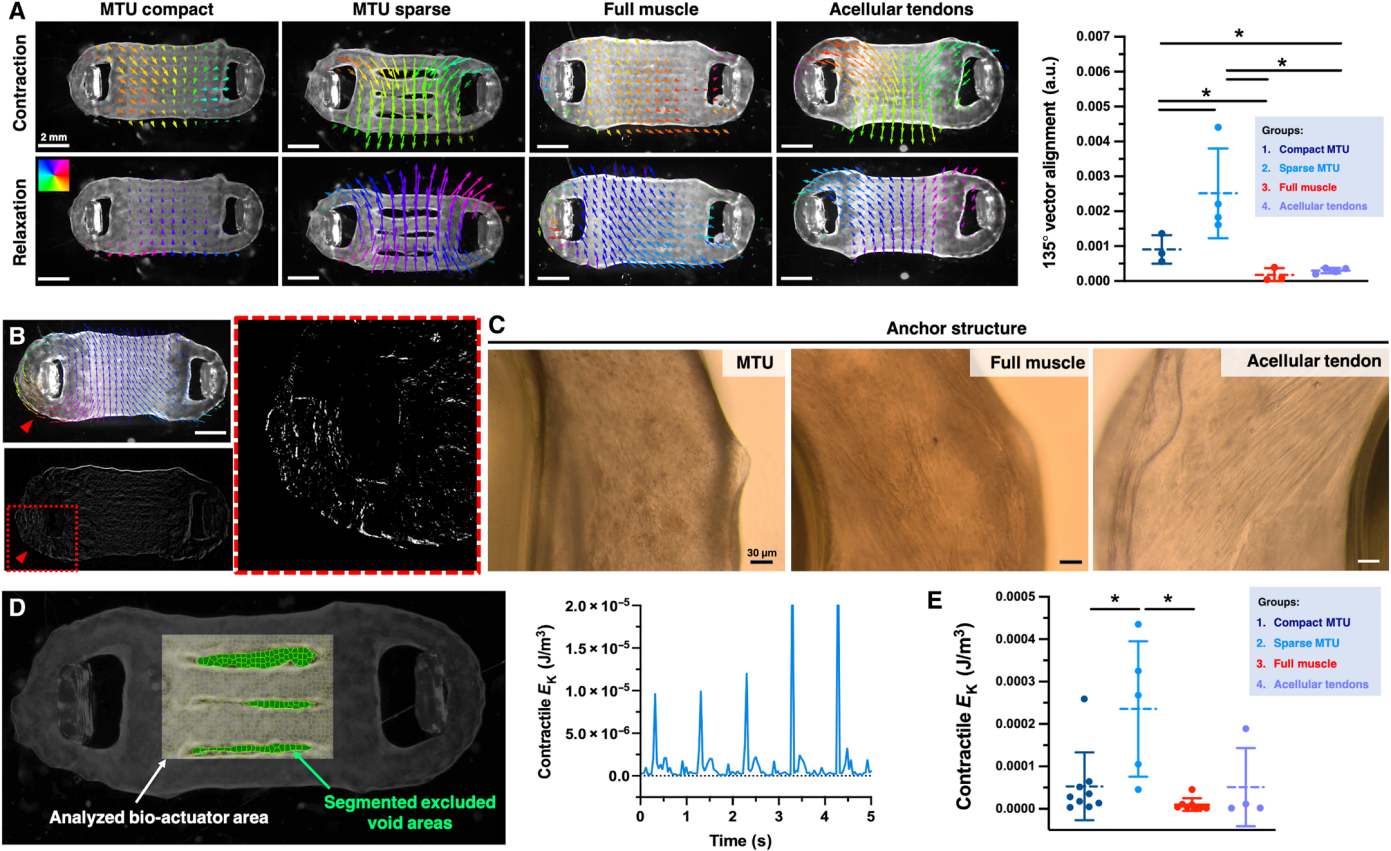
Characterization of the active mechanics. (**A**) Optical flow (pixel-wise velocity) of MTUs and control tissues during stimulated contraction with applied electrical fields (1 Hz) on day 15 of culture. The contraction angle is colored using a hue, saturation, value (HSV) wheel. Arrow sizes were scaled (25×). Amount of motion flow vectors aligned with a specific direction (135°), as calculated via color quantification from optical flow videos of different construct groups (*n* ≥ 3). In the right panel of (A), *P* values are as follows: 0.048 (groups 1 and 2), 0.049 (1 and 3), 0.031 (1 and 4), 0.028 (2 and 3), 0.013 (2 and 4), and 0.287 (3 and 4). (**B**) Optical flow analysis (top) and image subtraction analysis of a construct with acellular tendons indicating motion in the anchor area (red arrowhead); zoomed-in view of the subtraction results on the anchor area (circumscribed by red dashed lines). (**C**) Optical microscopy of the anchor structure in the MTU, full muscle, and acellular tendon constructs. (**D**) Image segmentation (left) to select the muscle tissue in the MTU with a sparse design (culture day 15) and analyze the mass transfer by considering the displacement in the contractile direction, and time-dependent contractile kinetic energy (*E*_k_) (right) in contractile direction of an MTU during stimulated contraction at 1 Hz electrical pulse frequency. (**E**) Mean contractile kinetic energy (*E*_k_) of MTU and control constructs during stimulated contraction (1 Hz frequency; time frame of 5 s) (*n* ≥ 3). In (E), *P* values are as follows: 0.013 (groups 1 and 2), 0.156 (1 and 3), 0.970 (1 and 4), 0.002 (2 and 3), 0.081 (2 and 4), and 0.226 (3 and 4). Statistical significance is expressed as follows: *P < 0.05.

To assess the contractile performance of the MTU, we stimulated the contraction of the tissue with electrical pulses and analyzed the motion via optical flow analysis and image subtraction techniques (Fig. 6, A and B, fig. S10, and movie S1). To determine whether the studied groups generated different displacements, we assessed the lateral motion response during the contraction phases. Specifically, we calculated the alignment of the optical flow vectors to a selected direction (contraction angle: 135°) by performing the corresponding color quantification on the images (Fig. 6A, right). As compared to compact MTUs, the sparse counterparts generated larger displacements, as indicated by the larger optical flow vectors along this angle of contraction (Fig. 6A). In contrast, full muscle bioactuators and acellular tendon controls displayed more contained displacements.

In acellular tendon constructs, we observed local deformations occurring in the anchor areas (Fig. 6B and movie S2). Optical microscopy images also show cells in the anchor structure of acellular tendon constructs with similar morphology to cells in full muscle constructs, whereas cells in the anchor structure of MTU show a different morphology (Fig. 6C). These results further confirm myoblast migration from the central area to the anchor structure in acellular tendon constructs as well as formation of functional eSMT in the initially acellular anchor structure. While spontaneous contractions were frequently observed in the sparse MTUs, acellular tendons, and full muscle constructs, these events rarely occurred in the compact MTUs.

When electrical pulsed stimulation was applied to evoke contraction, the MTUs accurately followed the stimulus pattern (Fig. 6D and movie S3). To characterize the contractile performance of the MTUs, we computed the bioactuator’s contractile kinetic energy by performing image segmentation to select the central muscle without void areas and analyzing the mass transfer by considering the displacement in the contraction direction (Fig. 6, D and E). From the optical flow analysis, we determined the movement speed of each pixel in the MTU ( v*_i_*) and, as we assumed the tissue has a uniform density across all pixels, we calculated its kinetic energy as the quadratic sum of all individual velocity values v*_i_* multiplied by their corresponding masses. To get a measure that accounts for the entire MTU, we normalized this value by the total tissue volume, eventually obtaining the volumetric contractile kinetic energy *E*_k_.

We observed no significant difference in *E*_k_ between the compact MTU (5.3 × 10^−5^ ± 7.6 J/m^3^), full muscle (1.3 × 10^−5^ ± 1.6 J/m^3^) and acellular tendon (7.8 × 10^−5^ ± 0.9 J/m^3^) constructs (Fig. 6E), even if *E*_k_ values of sole muscle tissue were 3.9- and 5.8-fold lower than for MTU and acellular tendon controls, respectively, which suggests a behavior trend with smaller deformations. While acellular tendon constructs were initially seeded with the same initial cell number as MTUs, eSMT forms in the anchor structure as maturation progresses, potentially leading to an increased eSMT volume and comparable deformability results. In contrast, the sparse MTU had a significantly higher contractile *E*_k_ (2.5 × 10^−4^ ± 1.4 J/m^3^) than the compact MTU (Fig. 6E), indicating that a less compact structure with larger amounts of actuating eSMT augmented the deformation response.

Comparing sparse MTU with the other compact controls with full muscle and acellular tendons introduces at least two variables (i.e., design and cell composition), making the analysis inherently more complex. However, sparse MTUs displayed a statistically relevant increase of *E*_k_ when compared to compact full muscle constructs. While we did not observe any statistically significant difference between the sparse MTU and compact constructs with acellular tendons, the data suggest that a divergent trend exists, as indicated by a one–order of magnitude difference in the average values (means: 2.4 × 10^−4^ ± 1.4 J/m^3^ versus 7.8 × 10^−5^ ± 0.9 J/m^3^).

Last, the large data variability observed for the sparse design could be at least partially attributed to the loss of the initial configuration, which naturally occurs during tissue maturation. In particular, fusion among segments may cause the sparse design to resemble the compact configuration, thereby influencing kinetic energy variations (fig. S9). The varying extent of fusion could explain the disparities in the calculated kinetic energy values. Nonetheless, these observations indicate that a sparse design can overall enhance the motility of MTU systems.

### Mechanical properties and interface stability of the MTU assembly

**Fig. 7.**
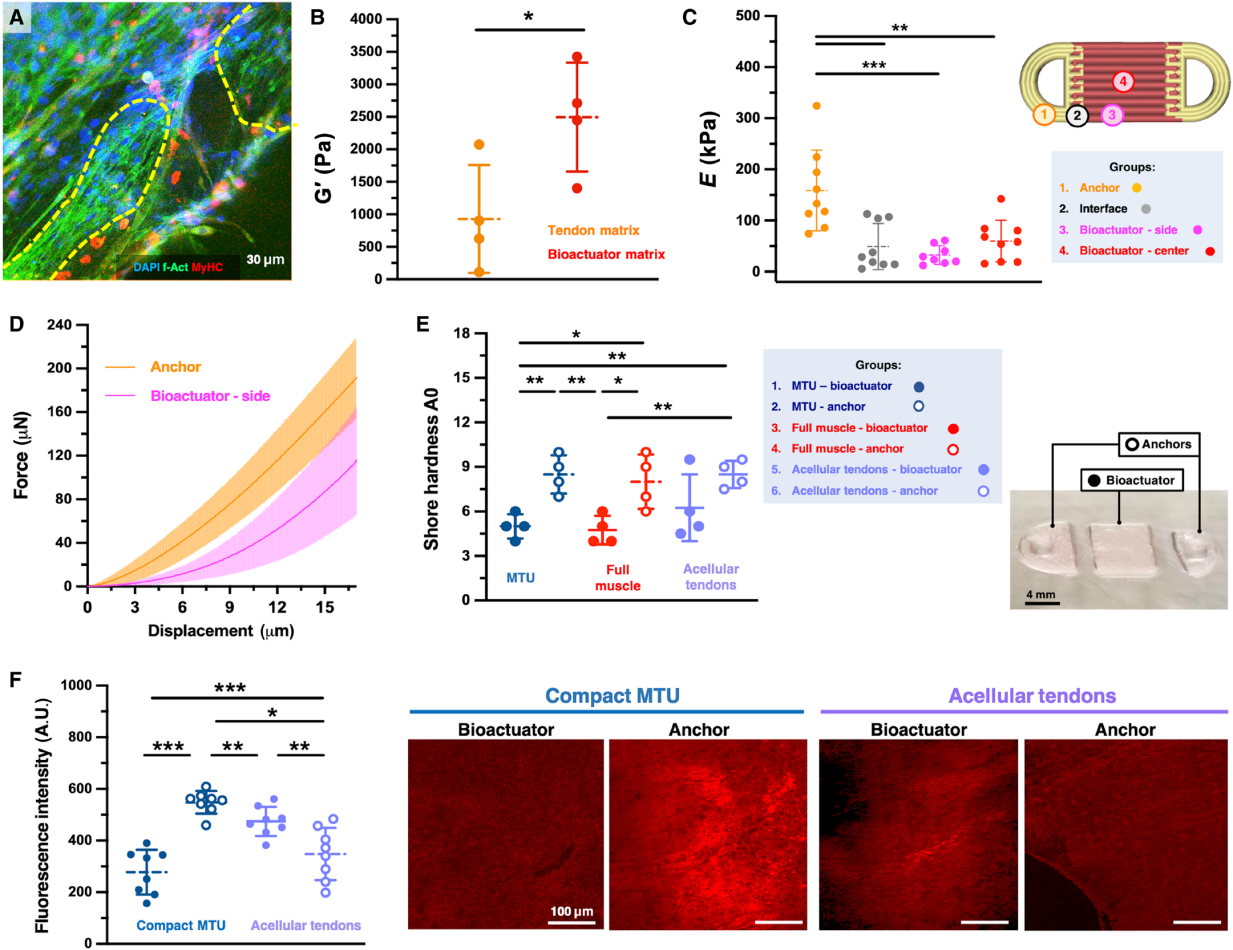
Mechanical properties of the MTU materials and interface. (**A**) Confocal imaging of the intertissue interface in a compact MTU construct (culture day 15), showing myofibers and clusters of fibroblast-shaped cells (encircled by yellow dashed lines), aligned along the tension axis. (**B**) The storage modulus of the polymer matrices (without cells) used to print the MTU, indicated as “tendon matrix” and “bioactuator matrix,” respectively. *P* value: 0.038. (**C**) Young’s modulus (*E*) measured via microindentation at different sites on the compact MTU (day 15 of culture): anchor area (representing tendon tissue, shown in orange), interface (shown in white), and bioactuator (representing muscle tissue) tested at lateral and central positions (magenta and red, respectively), as shown in the scheme. *P* values are as follows: 0.002 (groups 1 and 2), 5.17 × 10^−4^ (1 and 3), 0.004 (1 and 4), 0.345 (2 and 3), 0.602 (2 and 4), and 0.101 (3 and 4). (**D**) Representative force-displacement curves of the tested anchor and bioactuator areas. (**E**) Shore hardness of the MTUs and controls (culture day 15) with bioactuator and tendons’ areas indicated with full and empty circles, respectively (*n* = 4). *P* values are as follows: 0.004 (groups 1 and 2), 0.701 (1 and 3), 0.024 (1 and 4), 0.337 (1 and 5), 0.001 (1 and 6), 0.003 (2 and 3), 0.670 (2 and 4), 0.134 (2 and 5), 0.891 (2 and 6), 0.019 (3 and 4), 0.267 (3 and 5), 0.001 (3 and 6), 0.273 (4 and 5), 0.641 (4 and 6), and 0.113 (5 and 6). (**F**) Fluorescence intensity and representative imaging of tissue sections of compact MTU and acellular tendon controls (culture day 15) stained for picrosirius red revealing collagen in the matrices. Bioactuator and anchors’ regions are shown as full and empty circles, respectively (*n* = 8). *P* values are as follows: 1.61 × 10^−6^ (MTU bioactuator versus MTU anchor), 9.74 × 10^−5^ (MTU bioactuator versus control’s anchor), 0.008 (control’s bioactuator versus control’s anchor), 0.156 (MTU bioactuator versus control’s bioactuator), 1.51 × 10^−4^ (MTU anchor versus control’s bioactuators), and 0.011 (MTU anchor versus control’s anchor). Statistical significance is expressed as follows: *P < 0.05, **P < 0.01, and ***P < 0.001.

Furthermore, to assess the suitability of the construct for proper force transfer, we characterized the biomechanical properties of the muscle-tendon interface. In the interface areas, cells with different morphologies were found. Not only myotubes but also fibroblast-like cells displayed coherent directionality and aligned with the main construct axis (Fig. 7A).

As shown by rheological tests, the matrices for generating the bioactuator and anchor regions of the MTUs (referred to as “muscle” and “tendon matrix,” respectively) displayed storage moduli of approximately 0.9 ± 0.81 and 2.5 ± 0.81 kPa (Fig. 7B), suggesting that their combination could form a multicomponent assembly with heterogeneous mechanical properties. To quantify the stiffness differences across the matured tissue, we performed microindentation with a spherical cubic zirconia indenter with a 100-μm radius and recorded indentation curves for different areas in the MTU construct (Fig. 7, C and D, and fig. S11). Calculating the Young’s modulus (*E*) using a Hertzian contact model, we found that the *E* value in the anchor area was 3.4 times higher than in the bioactuator region (158.9 ± 78.9 versus 47.23 ± 34.3 kPa), confirming the increased stiffness of the fibroblast-seeded tendons. The *E* measured in the muscle-tendon interface zone significantly differed from the *E* values obtained for the tendon but not from those of the muscle tissue regions. However, these interface values (49.4 ± 45.1 kPa) were intermediate between those of the tendons and adjacent muscle areas (32.7 ± 18.4 kPa) and exhibited considerable variability, likely reflecting the stiffness heterogeneity inherent to a multimaterial interface.

Moreover, at the end of tissue maturation, the bioactuator and anchor components exhibited shore hardness values of 5.1 ± 0.81 and 8.5 ± 1.29, respectively (Fig. 7E), further confirming the stiffness heterogeneity. Statistically relevant differences were not found between the components of controls made of sole muscle bioink or containing acellular anchors, suggesting that the higher stiffness of the anchor regions is contributed by the polymer matrix composition and, to some extent, also by the presence of fibroblasts. In sum, the distinct mechanical properties of the MTU’s regions were retained during tissue development, generating a softer muscle bioactuator and more rigid tendon-like anchors.

We performed picrosirius red staining and quantified its intrinsic fluorescence intensity to assess the collagen deposition by cells (Fig. 7F). In MTUs, the matrix’s collagen content was higher than in the bioactuator’s region, with a 2.7-fold more intense fluorescence signal. In contrast, in controls lacking fibroblasts, the collagen content was higher in the bioactuator’s region than in the anchors. While the collagen content of the bioactuator's area was not significantly different between the MTUs and the controls with acellular anchors, one of the anchors’ area was (*P* < 0.05), suggesting that, even if both myoblasts and fibroblasts are involved in the production of collagen, fibroblasts might contribute to collagen-matrix enrichment to a higher extent. These results further confirmed data from collagen quantification via immunostaining and confocal imaging (Fig. 5 and figs. S5 and S7).

**Fig. 8.**
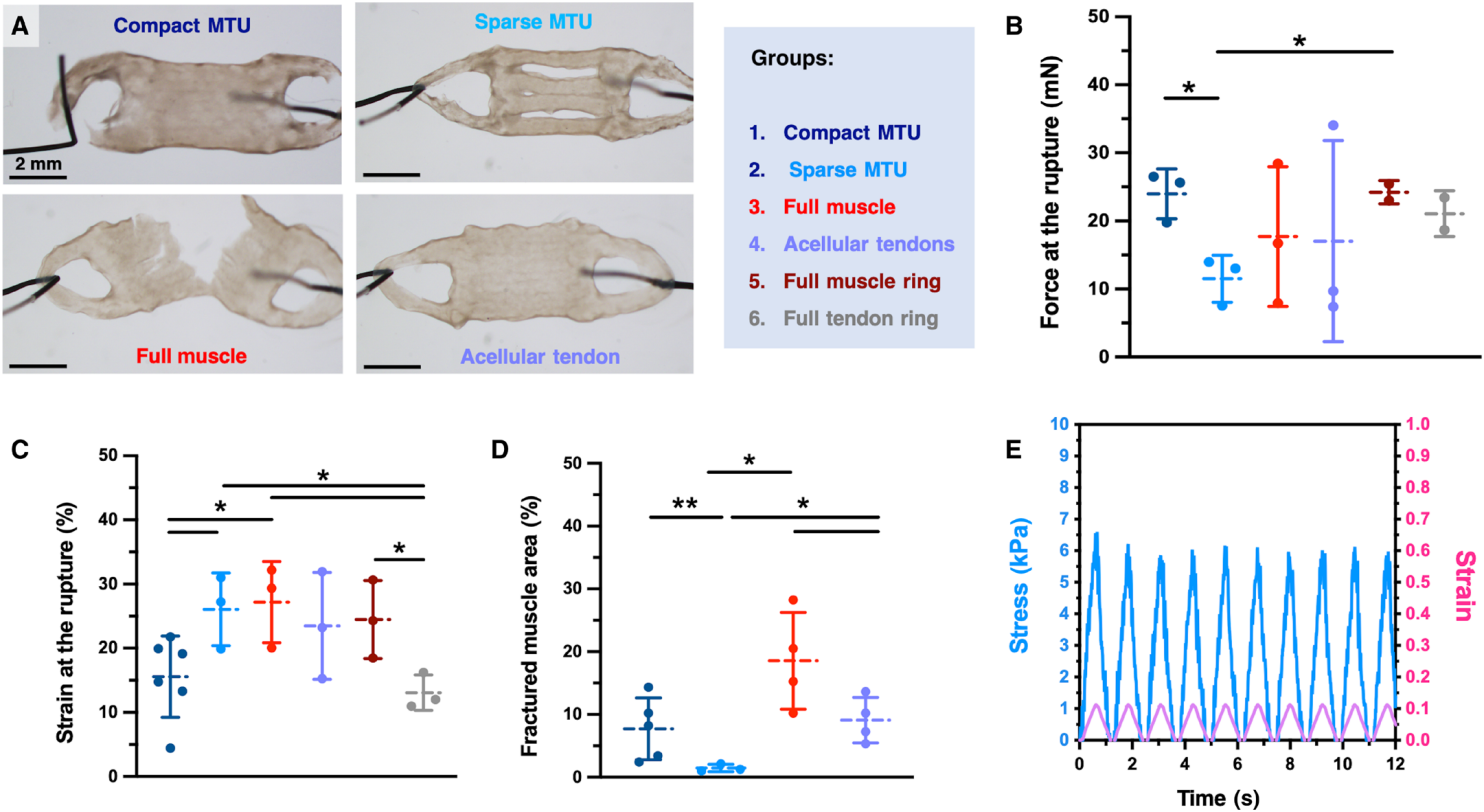
Characterization of the system’s mechanics. (**A**) Optical pictures of the tensile fracture tests, revealing recurrent breaking points of various constructs (day 15 of culture). Force (**B**) and strain (**C**) at the fracture point for constructs undergoing tensile testing after 15 days of culture. (**D**) Extension of the fracture area emerging in the muscle tissue after tensile tests. The fracture area is calculated as a percentage fraction of the fragmented or damaged area of the muscle (*n* ≥ 4). (**E**) Representative stress-strain response of the compact MTU subjected to a cyclic tensile test with an imposed displacement of 0.2 mm at a frequency of 1 Hz. In (B), *P* values are as follows: 0.0128 (groups 1 and 2), 0.376 (1 and 3), 0.474 (1 and 4), 0.935 (1 and 5), 0.437 (1 and 6), 0.378 (2 and 3), 0.562 (2 and 4), 0.019 (2 and 5), 0.055 (2 and 6), 0.952 (3 and 4), 0.459 (3 and 5), 0.698 (3 and 6), 0.562 (4 and 5), 0.742 (4 and 6), and 0.355 (5 and 6). In (C), *P* values are as follows: 0.046 (groups 1 and 2), 0.036 (1 and 3), 0.152 (1 and 4), 0.105 (1 and 5), 0.952 (1 and 6), 0.829 (2 and 3), 0.680 (2 and 4), 0.758 (2 and 5), 0.024 (2 and 6), 0.573 (3 and 4), 0.621 (3 and 5), 0.024 (3 and 6), 0.875 (4 and 5), 0.109 (4 and 6), and 0.042 (5 and 6). In (D), *P* values are as follows: 0.008 (groups 1 and 2), 0.056 (1 and 3), 0.654 (1 and 4), 0.013 (2 and 3), 0.017 (2 and 4), and 0.048 (3 and 4). Statistical significance is expressed as follows: *P < 0.05 and **P < 0.01.

We hypothesized that more collagen in the MTU anchor’s area would increase the stiffness and reduce the stretchability of the anchor component. Thus, we performed a tensile test to the rupture to assess the mechanical robustness of the constructs (day 15 of culture) and their components (Fig. 8A).

More than twofold higher forces were required to break the compact MTUs as compared to sparse MTUs (23.9 ± 3.7 versus 11.5 ± 3.5 mN, as shown in Fig. 8B), likely due to the smaller cross-sectional area. However, compact MTUs endured smaller strains (15.6 ± 6.3 versus 26.1 ± 5.7 percentage elongation; Fig. 8C), which confirms that less compact systems break under lower forces but are more stretchable. Controls composed of sole muscle bioink and containing acellular anchors also deformed more than compact MTUs but broke with lower forces (Fig. 8, C and D). To investigate how matrix composition influences mechanical behavior, we introduced two control systems composed solely of tendon bioink or muscle bioink, printed in a conventional ring-like design (referred to as the “full tendon ring” and “full muscle ring” conditions, respectively). Both matrices exhibited a similar force at rupture, but the muscle ring demonstrated a higher elongation at break (~24.5%), likely due to its lower stiffness, making it more compliant and capable of greater deformation under load. In contrast, the tendon matrix, with its lower strain at rupture (~13.1% elongation), was stiffer and more prone to brittle failure.

The distinct mechanical properties of compact and sparse MTUs are a direct result of their contrasting structural configurations. From the tensile tests, we estimated the *E* for the compact and sparse MTU being ~61.5 and 27.2 kPa, respectively (fig. S12). Having a continuous, solid material distribution, the compact MTU provides more resistance to deformation. In contrast, a sparse structure with holes or gaps has less material to bear the load, resulting in lower stiffness. In contrast, the sparse MTU, with gaps between parallel bridges, offers enhanced flexibility. Increasing the line spacing would further reduce stiffness by decreasing the continuity of the tissue. This relationship between line spacing and stiffness is a crucial factor in MTU design, offering potential for customizing the mechanical properties of the constructs to suit specific bioactuator functional requirements.

The bulk *E* values obtained from tensile tests are in line with expectations for eSMTs, which typically have an *E* in the range of 1 to 70 kPa (*25*). It should be noted that these *E* values do not substantially differ from those obtained from microindentation tests, which ranged from 30 to 160 kPa, depending on the analyzed tissue area. The existing differences in values are likely due to the distinct nature of these methods. While local stiffness measured via microindentation reports on the properties of small, specific regions, bulk stiffness, assessed via uniaxial tensile testing, represents the overall stiffness of the entire construct, resulting in averaged *E* modulus values (*42*). In addition, boundary effects may play a role, as local measurements are taken from isolated regions, while bulk measurements are influenced by the entire structure, including areas where stress might be concentrated or more uniformly distributed. Last, the bulk *E* values of the MTU might be dominated by the muscle component, as it constitutes the major fraction of the whole MTU. Consequently, the bulk *E* is expected to approximate the value range observed for the eSMT (~47.2 kPa, as measured via microindentation).

We never observed any fracture emerging at the MTJ site, indicating that the two tissues were well integrated. In contrast, the MTU constructs and the controls with acellular anchors broke in the anchor’s area that was attached to the force transducer’s pulling connector, likely due to the thinness of this structure and the brittleness of the anchor’s matrix. Sparse MTUs also broke in the same area, suggesting that the lighter bioactuator’s structure could retain its integrity under the applied stress. To assess the extent of damage inflicted on the bioactuators after the tensile test, we measured the fracture area within the muscle tissue and expressed it as a percentage of the total muscle tissue area (Fig. 8D). The full muscle controls frequently broke on the bioactuator region or displayed more serious events of bioactuator’s fragmentation, with larger damaged areas as compared to other groups (Fig. 8D). In the sparse MTU, the bioactuator region showed only minimal damage.

To verify the dynamic behavior of the MTU when constrained onto the pillars of the maturation template and electrically actuated, we modeled the induced deformations by subjecting the constructs to cyclic stress testing with variable frequencies (0.1 to 5 Hz) and displacements (0.2 to 0.8 mm). Throughout the cyclic loading, the stress response varied with the applied strain, reaching peaks of 5.7 to 6.5 kPa at a strain of 0.12 (Fig. 8E and figs. S12 and S13). The material’s deformation coherently followed the applied stress path without showing permanent deformation or hysteresis, confirming its elastic behavior.

### Force output and transmission

**Fig. 9.**
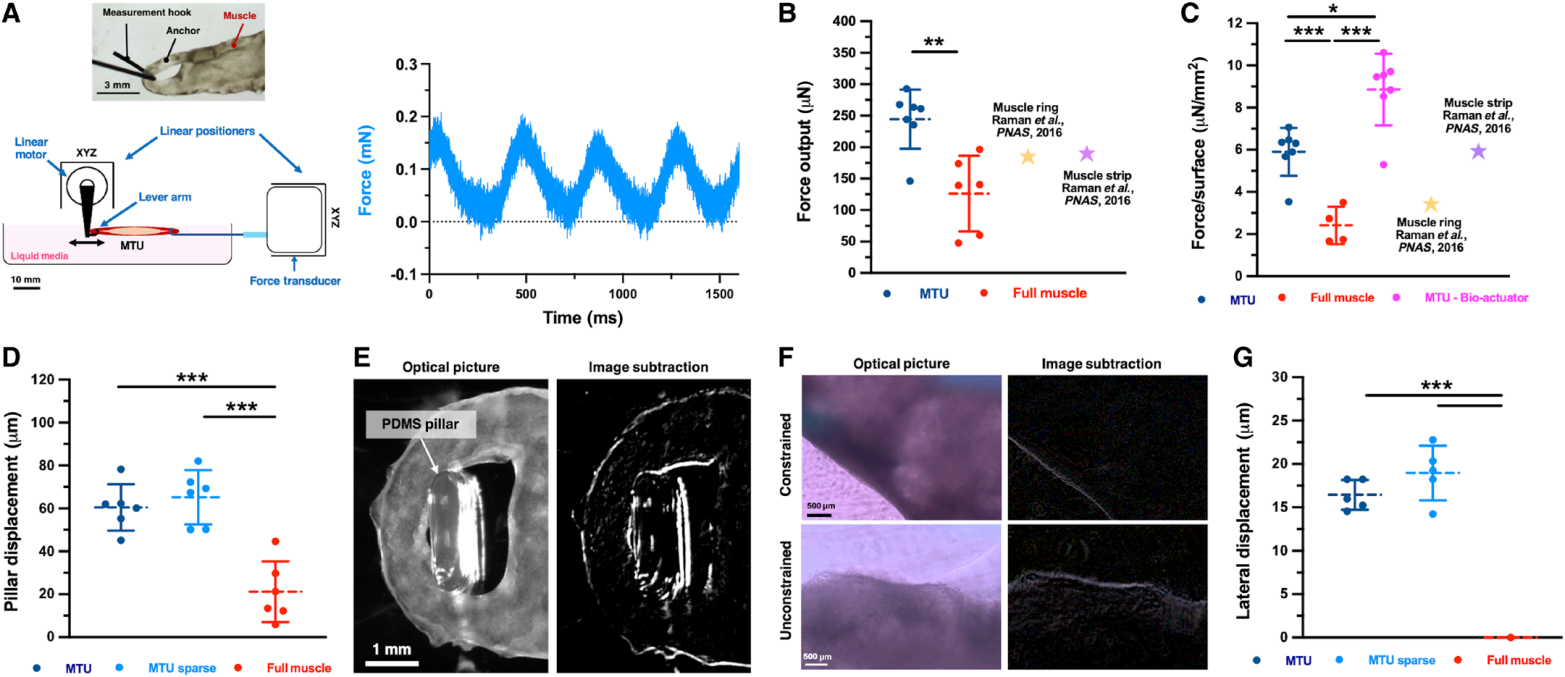
Force output and transmission. (**A**) Scheme of the force transducer setup and time-dependent force output of the compact MTU (culture day 15), showing synchronized muscle response with the applied electrical pulse pattern (1 Hz, 1 V/mm). Inset: metallic wires are looped in the anchors’ holes to connect the MTU to the transducer lever arm and a linear piezoresistive positioner fixed to a static point. (**B**) Force output of the compact MTUs and full muscle controls (culture day 15), and similarly sized and shaped constructs reported in the literature (*21*). *P* value MTU versus full muscle control: 0.002. (**C**) Force output normalized to the surface area (tissue-specific force) for full muscle controls, constructs from the literature, and MTUs (considering either the whole MTU surface or the MTU bioactuators’ surface only). *P* values: 5.1 × 10^−4^ (MTU versus full muscle), 6.7 × 10^−5^ (full muscle versus MTU bioactuator), and 0.002 (MTU versus MTU bioactuator). (**D**) Pillar displacement induced by actuated compact MTUs (culture day 15). *P* values: 0.501 (MTU compact versus sparse), 2.0 × 10^−4^ (full muscle versus MTU sparse), and 2.9 × 10^−4^ (MTU compact versus full muscle). (**E**) Representative picture of the anchor-pillar interaction during the MTU’s contraction (left), and image subtraction between relaxed and contraction states (right), highlighting the deformation area. (**F**) Representative optical pictures and image subtraction analysis revealing the contractile behavior of the compact MTUs after 3 months of tissue culture, as mounted or not in the maturation templates (indicated as “constrained” and “unconstrained,” respectively). (**G**) Lateral displacement calculated from image subtraction analysis, quantifying the motility of the constrained constructs (3 months of culture). *P* values: 0.156 (compact versus sparse), 3.73 × 10^−8^ (compact versus full muscle), and 1.1 × 10^−6^ (sparse versus full muscle). Statistical significance is expressed as follows: *P < 0.05, **P < 0.01, and ***P < 0.001.

To understand the tissue functionality in our MTUs, we measured their force output via a force transducer while stimulating contraction via electrical stimulation (Fig. 9A and figs. S9 and S14). The MTUs generated forces of up to 320 to 350 μN (Fig. 9B), with a 1.7-fold stronger output than that of the controls made of full muscle (approximately 245 versus 145 μN of average system force). The forces generated by the MTUs were also larger than those of similarly sized or designed muscle tissue constructs reported in literature, such as centimeter-scale ring and strip-shaped bioactuators electrically actuated with similar parameters (average force of approximately 185 and 190 μN, respectively, or lower) (*21*, *22*).

To understand whether the heterocellular nature of our system contributed to the force output, we assumed the construct’s surface to be the limiting factor on muscle tissue formation in all considered systems and then normalized the measured force to it (as calculated via image analysis) to obtain the “tissue-specific force” (Fig. 9C). MTUs were capable of approximately 6 μN/mm^2^, displaying a 2.4- and a 1.9-fold higher tissue-specific force than that of the full muscle controls and ring-shaped bioactuators (*21*), respectively. Even if bioactuators with a strip-like design produced normalized force values in similar ranges, MTUs’ tissue showed a better performance when the force was normalized to the area corresponding to the eSMT (i.e., the actual “bioactuator”).

As a consequence of the differential force production, the actuated MTUs induced stronger stresses on the pillars of the maturation platforms they were mounted on, causing their larger deformations (Fig. 9, D and E, and fig. S15). Nevertheless, the force and pillar deformation increments between the muscle controls and MTUs were approximately 100 and 200%, respectively. Thus, as the pillar deformation did not linearly change with the applied force, the dynamic behavior of the MTUs might be affected by other factors, such as the progressive variation of the interface’s mechanical properties that is provided by the fibroblast-populated anchors, which might enable more efficient force transfer to the synthetic structures of the biohybrid assembly. In particular, for our silicone pillars, we measured an *E* of 206 kPa, which falls within the stiffness range expected for printable resins with elastomeric properties, formulated with a low base-to-curing agent ratio and cured at low temperatures (~100 to 300 kPa) (*43*, *44*). This stiffness aligns with the *E* values measured for our muscle and tendon tissues (Figs. 7 and 8), helping to form a stiffness gradient, optimize tendon-pillar interaction, and reduce mechanical mismatch within the biohybrid assembly.

Last, our MTUs were viable and contractile for approximately 3 months (Fig. 9, F and G, and movie S4), thus largely overcoming the durability of similar muscle tissue–based bioactuators (approximately 2 to 3 weeks) (*21*). The contractile abilities of our MTU emerged early in tissue development with the onset of spontaneous contractions on day 5 (movie S5 and fig. S16), and the responsiveness to electrical stimulation persisted for several weeks beyond the typical tissue development period (2 weeks). In agreement with what was reported for these more freshly biofabricated and yet design-wise comparable systems, the active tension strain of MTUs was limited to minimal deformations (i.e., tens of micrometers of lateral displacement) when the constructs were mounted on their maturation platforms (*21*). Unconstrained MTUs exhibited more pronounced lateral movements (i.e., 100 to 200 μm per contraction) and achieved lateral displacement speeds of approximately 550 to 650 μm per minute, demonstrating strong contractility even after several weeks of maturation (fig. S17).

With this set of characteristics, our MTUs qualified as candidate bioactuators to engineer complex biomimetic models, especially replicating soft-hard hybrid anatomical systems where force transfer efficiency is crucial (fig. S18). Moreover, 3D bioprinting of combined muscle and tendon bioinks allowed us to explore additional musculoskeletal-inspired architectures. For example, we fabricated a core-shell structure, where the eSMT was encapsulated within an outer tendon layer (figs. S19 to S23). This design mimics the in vivo tissue organization, where muscle tissue is surrounded by connective layers (endomysium, perimysium, and epimysium) that transition into the tendon at the MTJ. Aiming to achieve efficient energy transfer via structural integration, the core-shell design enhanced the mechanical cohesion of the engineered construct.

## DISCUSSION

Living cells are unparalleled in their energy efficiency, converting biochemical energy into mechanical work with remarkable precision, which makes them ideal candidates for engineering systems such as biohybrid robots (*45*–*47*). Yet, to fully leverage cellular energetic efficiency, biohybrid systems need to be optimized for energy preservation, and engineering interfaces for minimized energy dissipation are crucial. Thus, to design efficient musculoskeletal systems actuated by living cells, properly integrated and biomimetic interfaces should be envisioned to ensure effective energy transfer across components and enhance the overall functionality and durability of the system. Musculoskeletal designs for biohybrid robots based on eSMT should include reasoned strategies for effective energy use in realizing motion.

To promote in vitro maturation of the eSMT or use it as a bioactuator, the eSMT is typically combined with synthetic and more rigid elements that form tissue maturation templates or robotic systems (*17*, *21*, *25*). These biohybrid assemblies entail the intrinsic challenge of creating a mechanical mismatch between the soft tissue and the artificial components. Such abrupt disparities in the mechanical properties of the components limit the stability of the assembly and reduce the force transfer from the bioactuators to the surrounding system. Engineering well-matched muscle-skeleton attachments (apodemes) in biohybrid robotic devices can improve their functionality and accelerate their real-world applications (*3*).

In this work, we aimed at blurring the stiffness mismatch in the muscle-robot interface to achieve more performant eSMT-based bioactuators. Inspired by the efficiency of the musculoskeletal system, we engineered living tendon-like anchors that incorporate intermediate mechanical properties for a seamless interface between the muscle tissue and PDMS elements. We 3D-bioprinted a linear assembly with a “tendon-muscle-tendon” configuration by flanking a myoblast-laden block with two fibroblast-laden structures. The tendon-like anchors were printed using a stiffer matrix to provide mechanical support while being further enriched with collagen secreted by the embedded fibroblasts. Moreover, these cells promoted eSMT maturation, which resulted in larger myofibers and force outputs, as demonstrated by tissue analysis and dynamic tests. These findings highlight the dual role of the fibroblasts in reinforcing the interface mechanically and biologically enhancing muscle tissue development. Last, to ensure the stability of the assembly, we stabilized the tissue interface by 3D bioprinting an interdigitated zone that mechanically interlocked the two tissues and closely mimicked the native MTJ.

To replicate the MTJ’s complex architecture, high-resolution biomanufacturing (like 3D bioprinting) is often used (*15*, *48*). Our construct was realized from polymeric matrices that were optimized for proper tissue development, allowing for the formation of robust fibroblastic tissue and contractile eSMT. In particular, the development of contractile, functional eSMT in our system unlocked dynamic studies that were precluded in other MTU studies, in which the eSMT was not reported to contract.

For example, Merceron *et al.* (*19*) fabricated biphasic MTUs by alternating the printing of thermoplastic materials seeded with C2C12 and NIH/3T3 cells, respectively. These constructs displayed good cell biocompatibility, expression of MTJ-related genes, and biomimetic system mechanics comprising an elastic muscle-like side and a stiffer tendon-like side. Nevertheless, no functional activity of the muscle was reported. Similarly, other MTU-focused studies have provided a biological and mechanical characterization of MTUs (*12*, *15*, *49*). Still, none of these studies investigated the dynamic responsiveness of the stimulated muscle or the effects of the coculture system on the eSMT’s development.

Thus far, only Laternser *et al.* (*50*) demonstrated that co-fabricating human primary skeletal muscle cells and rat tenocytes could result in 3D dumbbell-shaped MTUs with successful differentiation of both tissues and biological functionality of the muscle tissue, as assessed via calcium imaging. Nevertheless, their study did not report on how the contraction force was affected by the coculture system and lacked a quantitative assessment of the force acting through the mechanics of the system, which is crucial for replicating the dynamic functionality of the musculoskeletal system and enabling more adaptive, efficient, and responsive biohybrid systems.

In contrast, our MTU was conceived to operate in a dynamic system that mimics the musculoskeletal system arrangement, with a functional bioactuator that interfaces to rigid elements through two flanking tendons. Thus, we considered that such a triple-unit composite would require stability and aimed to reinforce the tissue-tissue interface with an intricate printing design. By exploring different MTU-printed interface designs, Kim and Kim (*20*) showed that including a “cell mixing zone” in the MTU increased the stability of the assembly and the expression of integrin β1 and MTJ-associated genes. Inspired by that work, we have proposed an alternative arrangement of overlying tissue-tissue interlocking layers and bioink mixing zones, which guaranteed full stability during the tensile tests to rupture.

In our study, we investigated the biological dimension of the engineered system and highlighted the advantages of combining a bioactuator with living tendons. Moreover, beyond the biological characterization, our study also investigated the dynamic behavior of the engineered systems and aimed to highlight potential relations with the biological findings. To validate our hypotheses, our study plan included multiple control systems, such as constructs composed solely of muscle tissues or tendon tissues, systems with acellular anchors, and MTUs with varying interface designs.

We first formulated muscle and tendon bioinks suitable for extrusion bioprinting and fine-tuned their mechanical properties to introduce a stiffness gradient from the soft eSMT through the tendon-like components, and to the synthetic PDMS pillars. As an initial interesting observation, we reported that in the constructs featuring anchors printed with no cells (i.e., acellular tendon systems), during tissue culture, the myoblasts migrated from the bioactuator zone to the anchors’ region. As a consequence, eSMT formed and produced spontaneous and stimulated contractions within the anchor structures that were supposed to remain passive elements within the assembly.

In contrast, the presence of fibroblasts in the MTUs effectively prevented myoblast migration into the anchors’ components and resulted in a compartmentalized system with a clear distinction between active (muscle) and passive (tendon-like) components. We argued that the mechanisms responsible for such an effect are related to spatial occupancy, as well as cell physical confinement and migration inhibition. In the first place, a high cell concentration in certain areas can generate physical obstacles, reducing the available space for other cells to migrate into those regions. The cellular mechanisms regulating spatial occupancy are contact inhibition (*51*) and plithotaxis (i.e., tendency to migrate along the local orientation of maximal principal stress) (*52*, *53*). In regions of high cell density, such as the fibroblast-populated anchors in our MTU, the collective mechanical forces and stress fields are likely to produce physical constraints limiting the migration of myoblasts into these areas.

Second, cell density alters cell migration dynamics by limiting cell contraction and integrin-mediated adhesion function (*54*–*56*). These cell responses are typically mediated by mechanotransduction intracellular pathways, which regulate how cells perceive and react to their surroundings, affecting their motility (*54*, *57*). In addition, the extracellular matrix and adhesion molecule availability are modified in multicellular environments, further restricting migration (*58*).

In our system, we could observe that fibroblasts confined myoblasts to a specific hydrogel area throughout the tissue maturation process. This compartmentalization strategy can be valuable for tissue engineering applications requiring one to maintain precise spatial organization of different cell types and replicate the complex architecture of native tissues (*59*–*62*). Kriegman *et al.* (*63*) showed how combining passive and active tissue components enables the realization of functional living systems with specific dynamic properties. To manufacture simulation-optimized designs made of discrete passive and active tissue portions, the group used micromanipulation and microcauterization techniques to merge tissues from amphibian cardiomyocytes and epidermal cell progenitors in diverse architectures. Such reconfigurable living machines locomote with reproducible behaviors for a period of days or weeks, suggesting the retention of the tissue discretization in the design. These findings provide insight into cell migration dynamics and their implications for biohybrid robotics and tissue engineering.

To define the tissue maturity degree, we provided a morphological tissue analysis encompassing parameters typically adopted in eSMT characterization, such as the fusion index, shift angle, and myotube width (*64*). In the first place, we showed that coculturing myoblasts with fibroblasts enhances myogenic differentiation compared to the controls, as evidenced by increased myotube width and fusion index. While various morphological features of myotubes can report on the tissue maturation state, the fusion index can be considered as an indicator with a primary relevance in predicting the force generation ability of the tissue, as it determines the percentage of myoblasts that successfully fuse into multinucleated myotubes (*65*). As force generation in muscle largely depends on the number of nuclei that support protein synthesis and cellular function, one can assume that higher fusion results in larger, more mature myotubes with greater contractile force. Moreover, even if MTUs displayed higher myotube width as compared to controls, it has to be mentioned that myotube width (as well as other structural features like the length and aspect ratio) per se is a less influential parameter and should be evaluated as combined with other indicators (*66*). Broader myotubes with poor fusion may not generate as much force as thinner, well-fused myotubes. Nevertheless, myotube width correlates with maturity and sarcomere packing density, contributing to overall force output.

Last, we provided myotube orientation data (as quantified by the shift angle indicator), which, in light of maximizing the force output, is also a crucial parameter (*67*, *68*). While other parameters (e.g., fusion index, myotube length, width, and aspect ratio) contribute to force production, myotube misalignment can dramatically impair functional output even if those factors are optimized. For example, a high fusion index increases the number of multinucleated myotubes capable of strong contraction, but if these myotubes are not properly oriented, their collective force does not add up efficiently. Similarly, longer myotubes with more sarcomeres in series improve contraction efficiency, but only when properly aligned. Thus, myotubes’ orientation dictates the effective contribution of all other structural parameters to overall contractile performance (*69*).

Therefore, to optimize bioactuators, the focus should be on promoting high myoblast fusion and ensuring proper alignment, while maintaining appropriate elongation and width for sarcomere organization and mechanical performance. As in our system, the myotubes’ orientation is highly dependent on the construct’s design, and future research should look at optimizing MTU sparse architectures to preserve the mechanical tension’s effect on cell alignment.

At the end of maturation, the skeletal muscle and tendon tissues of our MTU constructs exhibited lower stiffness compared to their native counterparts (*70*–*73*), which aligns with trends observed in other tissues engineered from soft hydrogels (*74*–*78*). Our microindentation tests revealed Young’s moduli of 49.7 and 158.9 kPa for muscle and tendon, respectively, making the tendon 3.2-fold stiffer than muscle. eSMT prepared from hydrogel-based constructs (e.g., gelatin, fibrin, and GelMA) displays *E* varying in the 1- to 50-kPa range (*77*–*79*). In early-stage tendon constructs from soft hydrogels, *E* can vary between 50 and 500 kPa (*74*, *75*). Enriching tendon-building hydrogels with particulate fillers can largely increase the *E* to 100 to 400 MPa (*80*).

While our stiffness values fall within the range reported for eSMT and early-stage tendon constructs generated from hydrogel matrices, differences with the native human and murine tissues persist. Native human skeletal muscle and tendon tissues typically have *E* in the range of 10 to 60 kPa and 500 to 1500 MPa, respectively (*72*, *81*–*83*). Native murine skeletal muscle and tendon tissues exhibit *E* of approximately 12 kPa and 2 to 36 MPa (in the mouse tail tendon) (*70*, *84*). This discrepancy of value ranges between engineered and native tissues highlights the structural limitations of hydrogel-based systems, revealing the need for future optimization.

To improve the perfusability of the MTU and increase the available surface for muscle tissue growth, we developed a sparse MTU design featuring a four-bridged muscle structure and studied its biological and dynamic functionality in comparison with its counterpart with a compact design. We noticed that, even though there was no significant difference in the fusion index, the compact MTU formed significantly wider myotubes with a better alignment than the sparse MTU. We argued that the central bridge elements of sparse constructs might not experience equivalent stress during tissue maturation under mechanical tension; thus, future research efforts will look at adapting the maturation platform for intimate interaction with the sparse MTU tendons and even tension distribution for improved myotube alignment. Nevertheless, in agreement with simulation data, the sparse MTU demonstrated the highest amount of deformation, likely due to the larger amount of skeletal muscle tissue combined with the lighter structure.

Our study aimed to provide an in-depth understanding of the functional capabilities of bioactuators and their composition and design variations. We carried out a mechanical and actuation characterization that enables a more comprehensive understanding of the system’s ability to move and deform; to generate contraction force along the tension axis; and to efficiently transfer the force from the muscle to the tendon and ultimately to the rigid synthetic elements. To fairly understand the dynamic response of MTUs and control groups to stimulated contraction, we carried out an analysis that took into consideration the amount of tissue that actively contributed to the resulting motility. For example, we focused the optical flow analysis and the quantification of the contractile kinetic energy exclusively on the central area of the construct that corresponded to the bioactuator area in all MTUs and control groups. Similarly, we reported force output production as normalized to the surface constructs’ area and considering the MTU’s bioactuator region only.

While, as mentioned above, the kinetic energy of MTUs was found significantly higher than that of other systems, no significant difference was observed between MTUs, full muscle, and acellular tendon constructs. This observation suggests that lightening the design and providing more surface for muscle tissue expansion is crucial to determining the higher motility of the system. However, because the total contractile kinetic energy alone does not directly reflect the force generated along the tension axis, we then directly measured the force output of the MTUs, learning that our system was capable of large force performance. Normalizing the force to the bioactuator’s area revealed that an eSMT cultured in the presence of fibroblast-seeded anchors develops higher strength (on average, almost 9 μN/mm^2^) than constructs realized with larger amounts of pure muscle tissue. In addition to describing the directional force production and the impact of mechanical properties on the overall actuation dynamics, we investigated whether introducing stiffer tendon-like anchors would reduce energy loss to deformation of soft bodies without eventually being transferred to the synthetic elements of a maturation template. Video analysis demonstrated that a greater displacement of the pillar occurred during MTUs’ actuation, which was caused by the larger force output of the MTU bioactuator and the presence of a mechanical gradient in the interface. Together with the biomimetic MTJ-like interface, high assembly stability, tissue maturity degree, and functional durability, the energetic efficiency contributed to the high biomimetics of the natural musculoskeletal system, shown by our proposed MTU system.

In terms of alternatives, current state-of-the-art bioactuators, such as those developed by Raman *et al.* (*21*, *22*, *85*) (e.g., muscle rings generating 138 to 300 μN of force), produce a similar range of force outputs. However, such a design lacks the hierarchical complexity of our MTU constructs, particularly the biomimetic MTJ, which significantly improves force transmission efficiency and mechanical stability. Moreover, our approach emphasizes tissue-specific force, achieving higher alignment and maturation of muscle fibers through fibroblast coculture, as a clear advancement over other systems. Nonetheless, for broader applicability, particularly in biohybrid robotics or therapeutic devices, overcoming the inherent limitations of cell-laden hydrogels and their mechanical properties is essential.

Whereas our 3D-bioprinted MTUs demonstrate biomimetic designs and promising contractile responsiveness, the generated force output remains modest for practical applications. Even if aligning with expectations for eSMT bioactuators, MTU’s forces remain significantly lower than those produced by native skeletal muscle. With a peak contractile force of 300 to 350 μN, these constructs are below the threshold required for real-world robotic or biomedical tasks, where forces in the millinewton to newton range would be necessary (*86*, *87*). Despite the comparative improvements in tissue-specific force achieved through our system, the absolute output remains limited, which, as for current biohybrid actuators, decreases the immediate usability of the system outside controlled laboratory settings (*3*).

The force-generating capacity of biohybrid actuators spans a wide range, as it is influenced by a multitude of variables related to the choices in cells, biomaterials, and design criteria. Even if principles of tissue engineering and development leveraged the few micronewton forces of single cells to the millinewton scale in multicellular constructs (*7*, *10*, *11*, *88*), biohybrid actuators still fall short of the force output seen in traditional artificial actuators (*3*). The opportunities to address this limitation include strategies to promote cell functionality and tissue maturation (such as muscle tissue training) (*8*, *10*, *89*), simulation-driven optimization of design for specific task performance (*9*), and soft materials and assembly design for maximized agility (*90*). In our work, we found that living tendons had a role in both enhancing the contractile response of myofibers and the effectiveness of interacting with synthetic skeletons, being promising to effectively convert muscle contractions into mechanical work within biohybrid robots.

It has to be mentioned that, while our research demonstrated enhanced force generation, the achieved force output remained lower than some prior works (*11*, *91*), raising the need for a broader discussion on performance benchmarks. Even if our results in terms of force output were not the highest in absolute terms, we highlight that contractility data should be considered in light of the specifics of the studied system, and direct comparisons with other reported systems should be accompanied by considerations on the composition and design differences. To fully capture the relevance of introducing mechanical gradients for interfacing robotic muscles, we validated our hypothesis by experimenting on geometry- and size-matched control constructs lacking the tendon-like interface. In addition, we normalized the force output to the muscle’s active surface area and compared it to similarly sized bioactuators from other studies (*8*). Our findings indicate that the specialized interface enhances force transmission and suggest that applying this strategy to existing high-force bioactuators could push performance beyond the current state of the art.

Looking at bioactuators’ applicability, another hurdle is the inherent fragility and metabolic demands of living tissues. While relevant to the proposed system and similar ones, our data about long-term contractility, structural stability, and advanced performance do not overcome the critical issue of scalability for translational implementation of biohybrid robotic systems. Future advances in perfusion systems, resilient bioinks, and hybrid designs incorporating synthetic materials could bridge this gap (*4*, *25*, *92*). Moreover, the MTU’s modular design represents a potential pathway for scaling, pointing future research to robust multiunit assembly based on clustering multiple MTUs. While the utility of our constructs for replicating complex dynamic biological systems (such as middle ear models, fig. S18) showcases the potential of biohybrid robots for niche applications, their extension to tasks demanding precise, high-force output remains to be further investigated.

Last, the versatility of the multimaterial biofabrication opens perspectives to investigate more biomimetic designs of the tendon-muscle segment based on fibroblast-muscle cell cocultures (figs. S19 to S23), as well as more competent or clinically relevant cell types, such as muscle cells and tenocytes of human origin (*50*, *74*, *93*). While extrusion-based 3D bioprinting efficiently and reliably produced the desired MTUs with a high printing fidelity and more than 15 constructs printed in one go, it has to be mentioned that other 3D printing and bioprinting methods with higher space resolution hold the potential for fabricating intricate constructs with enhanced functionality. In the future, techniques such as laser-assisted bioprinting, which achieves single-cell resolution (~10 μm), inkjet bioprinting (10 to 50 μm resolution), and stereolithography or two-photon polymerization (20 to 50 μm resolution) might allow for precise control over the structure and organization of engineered tissues (*94*). For example, such high-resolution methods could serve to create MTU interfaces with higher densities of invaginations to produce larger contact areas between the two tissues. This way, high-resolution intricate designs could lead to more efficient force transmission and enhanced mechanical stability in biohybrid robots.

The eSMT’s unique ability to replicate motile functions of the native tissue was crucial in determining the emergence of the biohybrid robotic systems (*1*, *95*), and our work proposed to leverage the bioactuator’s dynamic ability to align with the energetic intelligence of musculoskeletal system designs. We provided insights into understanding force production across different bioactuator designs and, importantly, highlighted that tendons, being stiffer than the soft muscle tissue, can mediate the interface with the stiffer printable PDMS, thereby enabling effective energy transfer across the system elements. Our bioprinted MTU showcased the potential of designing and realizing bioactuators with strong similarity with the native musculoskeletal system’s mechanics.

Considering the broad versatility of the eSMT, our approach has the potential to inspire system engineering solutions across multiple domains beyond actuating dynamic soft machines, including pathophysiological studies and drug development, tissue grafts for regenerative medicine, and cultured meat production. In conclusion, our work demonstrated a pivotal step toward scalable, biomimetic muscle-tendon constructs that bridge the gap between tissue engineering and biohybrid robotics, providing a versatile, dynamic platform to advance both robotic systems and cell culture models.

## MATERIALS AND METHODS

### Cell culture and harvesting for 3D bioprinting

The murine myoblast cell line (C2C12) and fibroblast cell line were obtained from the American Type Culture Collection (Manassas, VA). Cells were cultured in a monolayer at 37°C in a 5% CO_2_-containing humidified atmosphere in a complete growth medium (GM). GM for myoblasts consisted of Dulbecco’s modified Eagle’s medium (DMEM) (D6429, Sigma-Aldrich) supplemented with 10% (v/v) heat-inactivated fetal bovine serum (FBS) (F7524, Sigma-Aldrich), 200 mM l-glutamine, penicillin (100 U ml^−1^), and streptomycin (100 μg ml^−1^; all from Thermo Fisher Scientific, Switzerland). GM for fibroblasts consisted of DMEM supplemented with 10% (v/v) FBS. For routine maintenance and expansion, the cells were seeded at an initial seeding density of 5 × 10^3^ cells cm^−2^ and detached from flasks by trypsinization (Trypsin EDTA 0.25%, Sigma-Aldrich) at 70% of cell confluency. For 3D bioprinting of the MTU, C2C12 myoblasts and NIH/T3T fibroblasts were harvested at 70 to 80% confluency by trypsinization and mixed at a concentration of 7 × 10^6^ cells ml^−1^ with the respective bioinks.

The bioprinted constructs were cultured in complete GM supplemented with 1 mg ml^−1^ 6-aminocaproic acid (ACA) (A2504, Sigma-Aldrich) (GM+). After 3 days of culture, the GM+ was replaced with differentiation medium (DM+), composed of 10% horse serum, 1% v/v penicillin-streptomycin, 200 mM l-glutamine, IGF-1 (50 ng ml^−1^), and ACA (1 mg ml^−1^).

### Bioink preparation

Gelatin methacryloyl (X-Pure GelMA, 160P40, Rousselot) was dissolved in GM supplemented with ACA (1 mg ml^−1^) at 120 mg ml^−1^. The photoinitiator lithium phenyl(2,4,6-trimethylbenzoyl)phosphinate (LAP) (900889, Sigma-Aldrich) was dissolved in phosphate-buffered saline (PBS) at 3 mg ml^−1^. Gelatin from porcine skin (G9136, Sigma-Aldrich) was dissolved at a concentration of 210 mg ml^−1^ in GM+. Fibrinogen from bovine plasma (F3879, Sigma-Aldrich) was dissolved in PBS at 100 mg/ml. GelMA and gelatin stock solutions continued to be stirred at 45°C. The fibrinogen stock solution was kept at 4°C until completely dissolved.

To prepare the cell-laden muscle bioink, C2C12 myoblasts were harvested and mixed at 7 × 10^6^ cells ml^−1^ with LAP (0.1% v/v), fibrinogen (10 mg ml^−1^), GelMA (25 mg ml^−1^), gelatin (35 mg ml^−1^), and Matrigel (20% v/v; Corning, 354234). For the cell-laden tendon bioink, NIH/T3T fibroblasts were harvested and mixed at 7 × 10^6^ cells ml^−1^ with LAP (0.1% v/v), fibrinogen (20 mg ml^−1^), GelMA (50 mg ml^−1^), gelatin (20 mg ml^−1^), and Matrigel (20% v/v). To prepare the acellular bioinks, the same steps were followed as for the cell-laden bioinks and the cell pellet was replaced by GM+.

### Bioprinting of 3D constructs

The 3D cell culture constructs were manufactured via extrusion-based bioprinting as three-layered constructs. The bioinks were aseptically loaded into different cartridges and kept at 4°C for 10 min. Then, the bioinks were extruded through conical nozzles with an inner diameter of 200 μm (27G) with the BioX6 Extrusion Bioprinter (CELLINK, Boston, USA). Before printing, the nozzles were aligned to determine their respective X-Y-Z offsets with a minimum 0.01-mm accuracy. To optimize temperature conditions and ensure an extrudable consistency, we induced gelatin/GelMA gelation by pre-cooling the bioinks to 4°C for 10 min. After printing, the 3D constructs were cross-linked with UV light (BioX6 Extrusion Bioprinter, 405 nm, 30 s) and by adding a 5 U ml^−1^ solution of thrombin (T4648, Sigma-Aldrich) in GM+ for 10 min at room temperature (RT) and 24 hours at 37°C in a 5% CO_2_-containing humidified atmosphere. After 24 hours, the cross-linking solution was replaced by GM+.

### Tissue imaging

After in vitro culture, the constructs were fixed overnight in 4% paraformaldehyde (PFA) solution at RT. The samples were washed several times with PBS. Then, the samples were embedded in paraffin, and sections of 4.5 μm thickness were cut with a microtome (Microm, HM430, Thermo Fisher Scientific). The sections were stained with hematoxylin (GHS116, Sigma-Adrich) and eosin (HT110116, Sigma-Aldrich). The slides were mounted and imaged in a bright field with a light microscope (Olympus CKX41, Olympus Schweiz AG). The sections were also stained for tissue-specific markers, F-actin (Alexa Fluor 488 phalloidin, R37110, Invitrogen) and DAPI (4′-6-diamidino-2-phenylindol) using a standard fluorescence imaging staining protocol. To assess skeletal muscle tissue development, MyHC (Myosin 4, eFluor 660, Clone: MF20, Affymetrix eBioscience) was used. To identify anchor tissue development, anti–collagen I antibody (ab21286, abcam) and anti-vimentin antibody (ab137321, abcam; dilution 1/250) were used. All images were taken using a confocal microscope (Zeiss LSM 780 Airyscan, Zeiss AxioObserver.Z1, ScopeM) and analyzed in Fiji ImageJ. Moreover, to quantify collagen, the tissue sections were stained with picrosirius red (365548, Sigma-Aldrich), imaged via fluorescence microscopy for the intrinsic fluorescence (λ_ex_: 540 to 560 nm; λ_em:_ 620 to 630 nm), and analyzed for fluorescence intensity with Fiji ImageJ. Imaging analysis was performed on at least three images from each analyzed sample feature (≥3 samples/condition; ≥3 experimental replicates).

### Tissue analysis

To characterize the connection area between the two tissues (i.e., interface), the bio actuator, and the tendons, we inspected the different areas of the constructs upon manual positioning of the microscope camera. The pictures referring to the tendons were taken from the external border of the anchors, while those referring to the bioactuators were taken in the central area of the muscle tissue. The pictures representing the interface were taken in the medial area of the anchor insertion spot as expected from the initial printing scheme (approximately 5 mm of distance from the anchor’s hole). Optical pictures of the interface of the muscle and fibroblast tissue were used to detect differences in the tissue texture in various areas of the MTU assembly by quantifying the signal intensity variance in selected regions of interest (Fiji, ImageJ), which were manually drawn in the related areas (i.e., muscle, tendon, and interface). The contrast between the different textures of the two tissues allowed us to visualize the interface of the two materials and estimate the radius (*r*) of the invaginations (as an indicator of the interpenetration) by measuring the distance from the base of the invagination to its distal peak.

To assess the maturity of our bioprinted muscle tissue, we calculated the fusion index and the shift angle from confocal imaging of the samples’ fluorescent staining. The fusion index is a quantitative measure used to assess muscle tissue maturity by evaluating myoblast fusion. To calculate the fusion index, we counted the total number of nuclei and the number of nuclei within MyHC-positive myotubes. The fusion index was expressed as a ratio between the number of nuclei within MyHC-stained myotubes and the total number of nuclei. The images were randomly selected from at least three regions from each sample, and the experiments were replicated three times. We defined the shift angle of the myotubes as the average angular deviation of myofiber orientations from a predetermined reference axis (usually the desired alignment direction). A lower shift angle indicates that the fibers are well aligned, suggesting higher tissue maturity and more efficient force transmission, while a higher shift angle reflects greater disorganization and reduced maturation of the muscle tissue. The alignment of myotubes was evaluated by measuring the shift angle between the long axis of the myotube and the *y*-axis direction (corresponding to the longitudinal axis of the constructs) by using ImageJ, according to existing protocols (*96*).

### 3D cell viability

To assess cell viability in the constructs, Live/Dead staining was performed 2 days after bioprinting, following the manufacturer’s instructions (R37601, Thermo Fisher Scientific). Calcein and propidium iodide were excited at 488- and 561-nm laser wavelengths, respectively, and imaged on a confocal microscope (Zeiss LSM 780 Airyscan, Zeiss AxioObserver.Z1). To assess the biocompatibility of the bioinks, cell-laden rings were printed. Alamar Blue assay (Invitrogen, DAL1025) was performed on days 1 and 4 of differentiation (days 5 and 8 of culture), and CyQUANT LDH Cytotoxicity Assay (Invitrogen, C20301) was performed on day 2 of differentiation (day 6 of culture) following the manufacturer’s instructions.

### Microindentation tests

We performed microindentation with a spherical cubic zirconia indenter (*R* = 100 μm) by using the FT-MTA02 micromechanical test station (FemtoTools AG, Baar, Switzerland). We tested two configurations: (i) a sample was immersed in PBS and (ii) a sample was dried in air for 10 min. The samples were fixed with a small amount of cyanoacrylate glue to ensure stable and well-defined mechanical boundary conditions. For each configuration, nine indentation curves were recorded in different areas of the MTU. To estimate the *E* from each indentation curve, we used a Hertzian contact model. We set the maximum indentation depth to 10% of the indenter’s radius (*d*_max_ = 10 μm). This indentation depth of 10 μm was consistently used for all tests, to ensure that the Hertzian contact formula provides an acceptable approximation

### Electrical stimulation of the tissue

On day 8 of differentiation, the 3D bioprinted constructs were stimulated using a function generator. Graphite rods connected to platinum wires were attached to the function generator, and electric pulses 1 V mm^−1^, 10 ms, and 1 Hz were applied. Simultaneously, video recordings were taken with a Zeiss Stemi 508 stereomicroscope and a Basler ace2 camera at 25 frames per second (fps).

### Characterization of the responsiveness to electrical stimulation

For calcium imaging, the 3D bioprinted constructs were stained with calcium-sensitive Fluo4 dye following the manufacturer’s protocol (F10489, Thermo Fisher Scientific). The constructs were electrically stimulated following the protocol previously described. Simultaneously, video recordings were taken with a Zeiss Stemi 508 stereomicroscope and a Basler ace2 camera at 50 fps. To characterize the connection area between the two tissues (i.e., interface), the bioactuator, and the tendons, we inspected the different areas of the constructs upon manual positioning of the microscope camera. The contractility of tissue during long-term stimulation was shown as displacement of the constructs’ border during actuation via image subtraction analysis (Fiji ImageJ).

### Optical flow analysis

As noninvasive optical flow analysis can provide information on the contraction of cells and tissues during video postprocessing (*97*), we used an open-source (OpenCV) optical flow algorithm with Gunnar Farneback’s algorithm to detect MTU contraction (*98*, *99*). The algorithm provided a dense pixel-wise velocity measure between different frames of the actuation video. The motion direction was colored using a hue, saturation, value (HSV) wheel. Arrow size and opacity were scaled with the magnitude of contraction. For visibility, arrow length was scaled by a factor of 25, and only a subset of arrows were shown by uniformly sampling 0.02% of the pixels. To assess directional motion (displacement) along a specific direction and compare the groups for displacement when stimulated in anchored states, we calculated the number of motion flow vectors aligned with a designated direction (contraction angle: 135°) using color quantification from optical flow videos of the different construct groups (*n* ≥ 3). Similarly, a superpixel algorithm from OpenCV (Simple Linear Iterative Clustering) was used for segmenting the void areas. Areas were clustered based on brightness, and we tuned the threshold values to best remove the dark void areas. Because we normalized results by the area of muscle, excluding these voids made a difference in the final metrics for comparison of the MTUs and control groups. Contractile kinetic energy can be computed from the optical flow images by knowing the depth of the construct to compute the volume of each image pixel. In particular, from the optical flow analysis, we know the velocity of each pixel $v_{i}\;$ , resulting in a kinetic energy of $\frac{\rho \;V}{2}\;\sum_{i}{v_{i}}^{2}$ , because we assume a homogeneous density ρ in each pixel of the MTU. Normalizing this quantity by the total MTU volume V , we found the volumetric contractile kinetic energy $E_{k}=\frac{\rho }{2}\;\sum_{i}{v_{i}}^{2}$ . Then, computing the mass from this because the density of the material is constant, which provides us with a mass and a velocity resulting in kinetic energy for each frame of the video.

### Numerical simulation

We performed simulations in the Differentiable Projective Dynamics framework for soft body deformation and contractile tissue modeling (*100*). We use a corotational material model, with a Young’s modulus of 49.7 and 158.9 kPa for muscle and tendon, respectively, and a Poisson’s ratio of 0.4 for both. The hexahedral mesh for the sparse MTU had approximately 3 × 10^5^ vertices, with exact values varying depending on bridge thickness and number of bridges. The anchors were simplified as 2 mm by 1 mm rectangular holes. Because we do not simulate the contact forces of the MTU with the anchors, the difference in anchor hole shape between simulation and reality can be neglected. The surface layer of the middle section was defined as contractile elements where we defined a fixed strain in each hexahedron along a principal contractile direction (parallel to the bridges). We dynamically simulated the construct for 0.4 s with 10-ms time steps, but only extracted the first and final frames for analysis, as we did not match the simulation and real-world muscle dynamics. The maximum deformation metric was computed by considering the deformation between the first and last frames of the simulation and computing the maximum magnitude of the difference in position in each vertex.

### ELISA for tissue and secretome analysis

The amount of IL-6 in the liquid media of constructs was quantified by ELISA, according to the manufacturer’s protocol. Briefly, media aliquots were collected and added to an ELISA kit plate coated with an antibody against the protein of interest (capture antibody), then a biotinylated secondary antibody (detection antibody) was added and the plate was incubated at RT for 2 hours. The reaction catalyzed by horseradish peroxidase was stopped by adding 1 M sulfuric acid and the absorbance was measured at 450 nm. The mouse IL-6 kit was purchased from R&D Systems (Minneapolis, MN, USA; product numbers: M4000B, M6000B, and M1300CB). To quantify the tissue MTJ-specific proteins, we homogenized the tissue after 15 days of tissue development and performed ELISA according to the manufacturer’s instructions (integrin β1: #A312184-96, VWR; periostin: #ab243689, abcam; laminin, #ab119572, abcam; paxillin, #50-252-8568, Thermo Fisher Scientific). Briefly, 10 volumes of lysis buffer per milligram of tissue were added and the samples were sonicated at low intensity for three to five pulses (each lasting ~5 s). Homogenates were centrifuged at 12,000*g* for 10 min and the clear supernatant (protein extract) was transferred to a fresh tube for further ELISA analysis.

### Rheology of polymer formulations

Rheological measurements of the acellular polymer blends were performed using a rotational MCR series rheometer (Anton-Paar, AT) with a parallel plate geometry. The polymer blends were deposited on the measuring surface of the rheometer plate (radius: 8 mm) and the upper plate was lowered until direct contact with the material was established. Frequency sweep tests were performed with a frequency range of 0.1 to 100 rad/s and a constant shear strain of 0.5%. All measurements were performed at RT. Matrix hydrogels were tested in cross-linked states, after irradiation with a handheld UV light (BioX6 Extrusion Bioprinter, 405 nm module, CELLINK, Boston, USA) for 30 s, followed by overnight incubation with thrombin (5 U/ml) at 37°C.

### Shore hardness test

To measure the shore hardness of different areas of the printed constructs, the constructs were manually cut to separate the anchor components from the central area. A tissue portion of equivalent size from anchors or centers was analyzed with an analog shore hardness tester (HB0 100-0, Sauter, Balingen, Germany). Measurements were performed at RT with four constructs analyzed per condition. In each construct, the two anchors were independently tested and the measurements were averaged. Measurements were not taken repeatedly on the same samples.

### Force measurements and tensile test

To directly measure the force output of our actuated MTU constructs (day 15 of culture), we used the Aurora Scientific 404C force transducer system and 302C linear positioner. The MTUs and control constructs were secured between the transducer lever arm and a post mounted to a linear piezoresistive positioner fixed to a static point. The MTUs were connected to the system by using metal wires bent to hook the anchor structures (or the rings in the case of ring-shaped control constructs). While maintaining the constructs in the cell culture medium to preserve tissue functionality, we applied electrical pulses (intensity: 1 V mm^−1^, pulse duration: 10 ms, frequency: 1 Hz) via field electrodes to elicit contractions, and co-recorded videos and output force data. Data were collected with the Aurora Scientific 600A software. To study the tissue interaction with synthetic components, we subjected MTUs to dynamic analysis while mounted on maturation templates consisting of two vertical pillars. The pillars were fabricated via extrusion 3D printing using the BioX6 Extrusion Bioprinter (CELLINK, Boston, USA), equipped with conical nozzles with an inner diameter of 200 μm (27G). The pillars were made from Dowsil 1700 E Clear Base (GA Lindberg, Kista, Sweden), a printable resin with elastomeric properties, formulated with a low base–to–curing agent ratio (20:1) and cured at low temperatures (37°C for 1 week). Pillars’ displacement was quantified via image subtraction analysis (Fiji ImageJ).

For tensile tests, PFA-fixed MTUs were mounted on the instrument in the same way, but the MTUs were stretched until rupture by allowing the arm to move away and take distance. The strain at the fracture point and the location of the fracture point were analyzed. To investigate how matrix composition influences mechanical behavior, we introduced two control systems with a ring design: one composed solely of tendon bioink and the other of muscle bioink, referred to as the full tendon ring and full muscle ring, respectively. These control systems used the optimized bioinks that were printed in a conventional ring shape instead of the typical MTU design. To quantify the damage caused by the tensile test on the bioactuators, we measured the fracture area within the muscle tissue and expressed it as a percentage of the total muscle tissue area. Image analysis was performed by manually defining regions of interest to segment the fracture zone and measure the total muscle tissue area. To determine the elastic behavior of the MTUs, the samples were examined with a uniaxial tensile testing machine (Instron 5942, Instron, Norwood, MA, USA) at RT. The constructs were washed, and their extremities were then carefully glued to two pieces of paper with a strong tissue adhesive glue. The papers were folded around the construct, which was then carefully positioned vertically on the sample holder of the tensile machine. The samples were characterized with cyclic stress tests, with variable frequencies (ranging from 0.1 to 5 Hz) and applied displacements (ranging from 0.2 to 0.8 mm).

### Statistical analysis

Data are presented as means ± SD, and statistical analysis was carried out using MS Excel Software. Biocompatibility was assessed by considering data acquired from at least three independent experiments and three technical replicates unless otherwise stated. Imaging analysis was performed on at least three images from each analyzed sample feature (≥3 samples per condition; ≥3 experimental replicates). Mechanical characterization was performed for three samples. Measurements were taken from distinct samples unless otherwise stated in the text. Sample exclusion from experimental data collection or analysis was allowed when samples featured evident defects or damages compromising the experimental performance or validity (e.g., contaminated tissue samples). We used a two-sided Student’s *t* test to determine the statistical significance of multiple datasets, including cell viability tests, sensor conductance, and sensor resistance response during the functional characterization experiments. Multiple comparisons between groups were performed using Tukey’s post hoc test. Statistical significance is expressed as follows: *P < 0.05, **P < 0.01, and ***P < 0.001. The absence of asterisks indicates no significant difference between the compared conditions.

## Supplementary Materials

### The PDF file includes:

Supplementary Text S1 to S16 Table S1 Figs. S1 to S23 Legends for movies S1 to S5 References
