# Supplementary material for: Multicellular muscle-tendon bioprinting of mechanically optimized musculoskeletal bioactuators with enhanced force transmission
Source: Sci Adv. 2025 Jul 16;11(29):eadv2628. doi: 10.1126/sciadv.adv2628 (PMC12266124; doi:10.1126/sciadv.adv2628)
Supplement: Supplementary file 1 — Supplementary Text S1 to S16 Table S1 Figs. S1 to S23 Legends for movies S1 to S5 References [file sciadv.adv2628_sm.pdf]

Science Advances
AAAS

Supplementary Materials for

Multicellular muscle-tendon bioprinting of mechanically optimized musculoskeletal bioactuators with enhanced force transmission

Miriam Filippi *et al.*

Corresponding author: Robert K. Katzschmann, rkk@ethz.ch; Miriam Filippi, miriam.filippi@srl.ethz.ch

Sci. Adv. **11**, eadv2628 (2025)
DOI: 10.1126/sciadv.adv2628

The PDF file includes:

Supplementary Text S1 to S16
Table S1
Figs. S1 to S23
Legends for movies S1 to S5
References

Other Supplementary Material for this manuscript includes the following:

Movies S1 to S5

S1. Supplementary Introduction

In the musculoskeletal system, the myotendinous junction is a highly specialized structure designed to efficiently transfer forces generated by muscle contractions through the tendon to the bone, facilitating movement. Recreating muscle-tendon units (MTUs) in vitro is essential for replicating this biomechanical efficiency in engineered systems, such as bio-hybrid actuators. In this work, we developed a functional 3D-bioprinted MTU system that integrates muscle and tendon-like tissues through a stable, biomimetic interface modeled after the natural myotendinous junction. The system consisted of a muscle bio-actuator based on C2C12 myoblasts, flanked by tendon-like anchors laden with NIH/3T3 fibroblasts and printed with an interdigitated interface designed to mimic the native junction.

To establish a mechanical gradient, the tendon matrix was engineered to be stiffer than the muscle bioink, enhancing force transmission. Immunostaining demonstrated that fibroblasts facilitated myoblast differentiation, myofiber maturation, and alignment. Histological and mechanical studies revealed that fibroblasts played a critical role in compartmentalizing tissue regions, limiting myoblast migration, and enriching the collagen matrix, which contributed to interface stability and mechanical robustness.

In our work, tensile testing confirmed the MTUs' mechanical stability, while COMSOL simulations informed design optimizations that improved force output. Compared to muscle-only bio-actuators, the MTUs generated higher force outputs and greater deformation of interacting synthetic elements, indicating superior force transmission. Dynamic testing revealed sustained contractility, with the constructs producing up to 350 µN of force and maintaining responsiveness for up to three months, outperforming previously reported systems.

Our work underscores the significance of mechanical optimization, biomimetic designs, and heterocellular co-culture in creating efficient bio-actuators. These advancements lay the groundwork for developing high-performance, energy-efficient dynamic systems with applications in biomedicine and robotics. Here below, we would like to report supplementary information and additional ancillary experiments that complement our manuscript and further inspire researchers in their next endeavors in the field of MTU fabrication.

S2. Bioprinting parameters optimization

To optimize the bioprinting process of the biphasic tissue constructs, we parameterized the extrusion processes of the muscle and tendon bioinks, and selected the most effective printing parameters combination for each of them.

	Muscle-Bioink	Tendon-Bioink
**Pressure [kPa]**	90-100*	90-100*
**Speed [mm/s]**	2	2
**Temperature [°C]**	RT**	RT**

Table S1 | Printing parameters of the muscle- and tendon-bioink.
* Corresponds to the starting printing pressure. For longer printing sessions the pressure was decreased accordingly. ** After preparing the bioinks, they were kept at 4°C for 10 min before printing.

The constructs shown in this work were fabricated using a co-extrusion process with two syringes. Importantly, the formulations chosen to create our bioinks are highly sensitive to temperature. In general, maintaining the correct printing temperature is therefore crucial to prevent both overgelation and undergelation, and achieve effective bioink's printability. In our work, to optimize temperature conditions, we pre-cooled the bioink to 4°C to induce gelatin/GelMA gelation and ensure an extrudable consistency. Pre-cooling the bioinks in the fridge (at least 10 minutes) proved to be sufficient to retain a stable consistency throughout the printing process.

S3. Stability over time

We optimized the bioink formulation and the culture protocol to increase the stability of the bioassembly during the maturation phase on the tensioning platform. When cells remodel the bioink matrix, hydrogel constructs exposed to mechanical tensioning tend to lose integrity and fracture at their connection point with the pillars of the maturation template (**Fig. S1**). By iteratively changing the composition of the hydrogel formulation and varying cytokine concentrations through different biofabrication and culture protocols, we identified the optimal parameter combination to obtain constructs that could endure mechanical tensioning.

Concerning the polymer components in our bioinks, we selected gelatin, fibrinogen, Matrigel, and GelMA, which are polymers commonly used to engineer skeletal muscle tissue in vitro.

Gelatin plays several crucial roles in muscle tissue bioinks, particularly in biofabrication and tissue engineering.(*101-104*) Firstly, gelatin provides a biocompatible scaffold that supports cell adhesion, proliferation, and differentiation, which are essential for creating functional muscle tissue. It also facilitates the formation of a gel-like structure that allows for the printing of complex tissue architectures, aiding in the structural integrity of the bioink. Gelatin's thermoresponsive properties make it adaptable during the bioprinting process,(*23, 105*) transitioning from a liquid

state at low temperatures to a gel at physiological temperatures, ensuring proper handling and molding. Additionally, gelatin can be chemically modified to enhance its mechanical properties and promote tissue maturation by influencing the extracellular matrix (ECM) formation, which is key for muscle tissue development. Overall, gelatin serves as both a structural and bioactive component, promoting tissue growth and function in muscle bioinks.

Fibrinogen contributes to the structural integrity and mechanical properties of the printed tissue constructs.(*106, 107*) As a key component of the ECM, fibrinogen is involved in the formation of fibrin networks, which help to stabilize and reinforce the tissue scaffold. When crosslinked, fibrinogen forms a gel-like structure that mimics the natural ECM, providing mechanical support for cells and facilitating their adhesion, proliferation, and differentiation. This process is especially beneficial in muscle tissue engineering, where the formation of a strong and organized matrix is crucial for promoting muscle tissue maturation and function.(*108, 109*) Additionally, fibrinogen aids in the modulation of the tissue's stiffness, making the bioink more suitable for the mechanical forces that muscle tissue must withstand.(*106*) The incorporation of fibrinogen in bioinks can also enhance the bioactivity of the construct, supporting muscle-specific cellular behaviors, such as myotube formation, which are important for generating functional muscle tissue.

Thrombin promotes fibrinogen polymerization to form fibrin networks, which provide structural support and enhance tissue formation.(*110*) In muscle bioinks, thrombin facilitates the creation of a stable, biocompatible scaffold that mimics the natural extracellular matrix, promoting cell adhesion, migration, and differentiation. By aiding in the generation of fibrin-based hydrogels, thrombin helps to guide myoblast alignment and fusion into myotubes, thus supporting muscle tissue development and improving the mechanical properties of engineered muscle constructs

Matrigel is a widely used ECM component in skeletal muscle tissue engineering due to its ability to provide a supportive, biocompatible environment for cell growth and differentiation.(*111*) Matrigel is a reconstituted basement membrane extract derived from mouse sarcoma cells and contains a variety of ECM proteins such as laminin, collagen IV, heparan sulfate proteoglycans, and entactin. These components mimic the natural ECM that supports cell adhesion, migration, and differentiation. Matrigel provides a scaffold for myoblasts and other muscle progenitor cells to attach, proliferate, and spread.(*112, 113*) The ECM proteins in Matrigel facilitate cellular interactions and ensure a suitable microenvironment for cell growth, mimicking the natural niche of muscle tissues. Matrigel also promotes myoblast differentiation into myotubes (muscle fibers) by mimicking the composition of the natural ECM that muscle cells encounter during development. The laminin and collagen found in Matrigel can enhance the alignment and fusion of myoblasts, which is essential for forming functional muscle tissue. Although Matrigel is not as mechanically robust as native tissue, it provides some mechanical integrity and stability to the growing muscle tissue, allowing for the application of mechanical cues that may further stimulate muscle maturation. Overall, Matrigel is beneficial in promoting skeletal muscle tissue development and its properties make it an essential material in the early stages of muscle tissue engineering for creating more functional muscle tissue constructs.(*114, 115*)

Gelatin methacryloyl (GelMA) plays a critical role in muscle tissue engineering due to its excellent biocompatibility and tunable physical properties.(*116-118*) Derived from gelatin, GelMA retains bioactive sequences that promote cell adhesion, proliferation, and differentiation—key factors for myoblast fusion and myotube formation. Its photocrosslinkable nature allows precise control over

mechanical stiffness and porosity, enabling the creation of scaffolds that mimic the native extracellular matrix. GelMA is often incorporated into bioinks to increase viscosity and enhance the rheological properties of bioinks, which improves printability and helps maintain structural fidelity during and after the bioprinting process.(*23*) This increased viscosity also supports cell encapsulation and uniform distribution within the printed scaffold.

Precisely, we tested several protocols to combine various amounts of these components, according to the following rationale. GelMA was used to achieve enough stability for the constructs' durability throughout the maturation process. However, aiming to retain ink extrudability and matrix softness for cell survival and appropriate tissue remodeling, we optimized the addition of GelMA in the muscle bioink within a low concentration range (eventually selecting 2.5% w/v). We selected a higher GelMA concentration (5% w/v) in the tendon bioink to create a stiffness gradient. Prior optimization tests defined 20 and 10% w/v as the highest GelMA concentration values for successful extrusion and cell spreading within the matrix, respectively. The concentration of gelatin was adjusted for optimal printability. Below, we explain different protocols used to refine the inks' compositions.

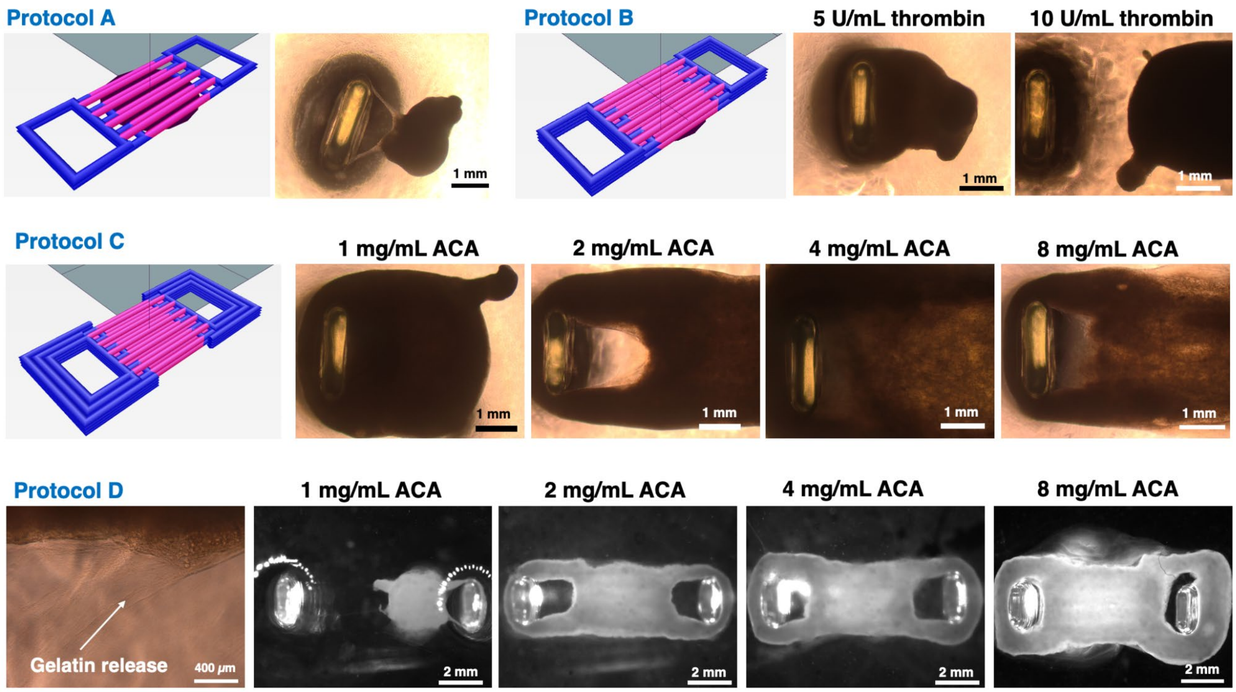

Fig. S1 | Stereomicroscopy of the MTU realized with different matrix compositions.

Detail of the tendon tissue attachment to the synthetic pillar in MTU realized with protocols described in the text below. In protocol B, constructs are shown with thrombin at 5 (left) and 10 U/ml (right).

In protocol A, we fabricated MTU constructs from the following two bioink formulations: 70 mg/mL gelatine + 20 mg/mL fibrinogen + 20% Matrigel for the muscle bioink and 70 mg/mL gelatine + 40 mg/mL fibrinogen + 20% Matrigel for the tendon bioink.

In protocol B, we used the same matrices to fabricate constructs with a higher layer number (i.e., 4), incorporating a single tendon line. We crosslinked the constructs with a thrombin solution that was tested at two different concentrations (5 or 10 U/mL). We expected that a higher thrombin concentration would have increased the crosslinking rate and improved the stability. However, the constructs were disrupted after detaching from the pillars, which suggested that an excessive condensation of the tissue occurred. In other trials, to stabilize the fibrin formation, the addition of calcium chloride was attempted, but the constructs dissolved after 10 min in the incubator, likely due to the interaction of the medium with calcium chloride, which created an exothermic reaction that led to gelatin dissolution (data not shown).

In protocol C, we used the same bioink as in protocol B, but the 3D-printed construct presented a thicker tendon region. We supplemented the culture media with 1, 2, 4, or 8 mg/mL of ACA. The constructs with 4 and 8 mg/mL in the culture media did not rupture and reached 8 days of maturation. However, they were not contractile.

In protocol D, we changed the bioink formulations to the following: 70 mg/mL gelatine + 10 mg/mL fibrinogen + 20% Matrigel for the muscle bioink and 70 mg/mL gelatine + 20 mg/mL fibrinogen + 20% Matrigel for the tendon bioink. We supplemented the culture media with 1

mg/mL, 2 mg/mL, 4 mg/mL or 8 mg/mL of ACA. Due to the lower fibrin concentration, we observed gelatin escaping the constructs. The constructs with 4 mg/mL and 8 mg/mL in the culture media did not rupture and reached 8 days of maturation. However, they were not contractile.

To improve the stability of the MTU, we incorporated GelMA into the two bioink formulations: 25 mg/mL GelMA + 45 mg/mL gelatine + 10 mg/mL fibrinogen + 20% Matrigel for the muscle bioink and 50 mg/mL GelMA + 20 mg/mL gelatine + 20 mg/mL fibrinogen + 20% Matrigel for tendon bioink. To investigate the cell biocompatibility and stability of the new bioink formulations, we fabricated constructs designed as rings. In protocol E, we compared the new formulations to the prior formulations of bioinks. The rings with the new formulations of bioinks reached 11 days of maturation, whereas the rings with prior formulations of bioinks ruptured after 7 days.

In protocol F, we fabricated MTUs using the muscle and tendon matrices containing GelMA. We observed spontaneous contractions on day 9 and were able to keep the MTUs in culture up to 29 days (**Fig. S2**).

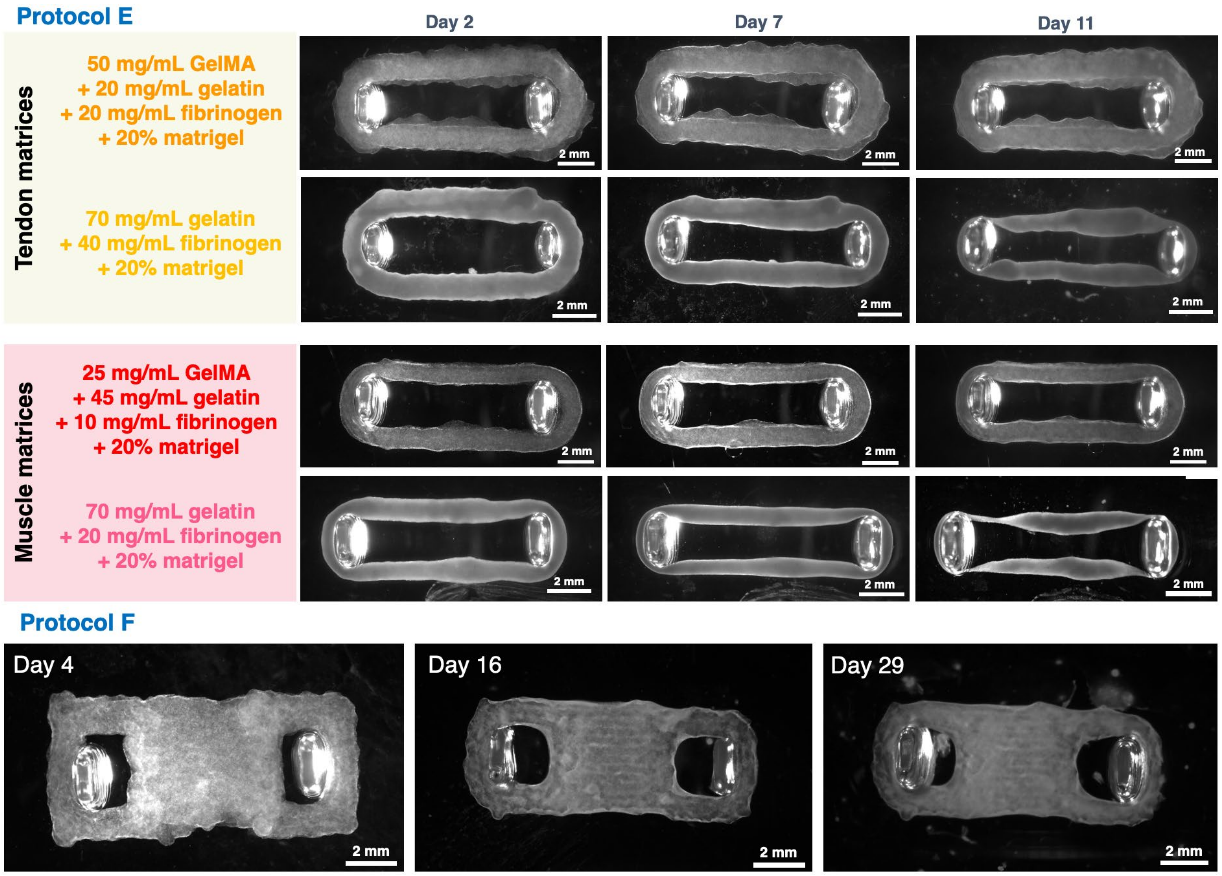

Fig. S2 | Stability and deformation of the constructs over time.

Stereotactic microscopy of the rings and MTU at different time points of the tissue development process realized with protocol E and protocol F.

Prior formulations of bioinks for the muscle and tendon components of the MTU contained 70 mg/mL gelatine + 20 mg/mL or 40 mg/mL fibrinogen + 20% Matrigel. Such a hydrogel formulation displayed a high softness, which caused issues with the stability of the bioassembly and favored the migration of muscle cells. As shown by confocal microscopy, tubular structures were found in various areas of the MTU differentiated for three days only, suggesting a spread distribution of myotubes (**Fig. S3**). Large multinucleated myotubes were observed at a high density in the muscle tissue and to a minor extent in the muscle-tendon interface, as well as in the anchor regions (which were expected to be populated by fibroblasts only). Cell nuclei were clustered and myotubes unidirectionally aligned parallel to the longitudinal axis of the construct, contributing to the anisotropic architecture expected for proper eSMT development. Myotubes were also observed in the interface area, which contained matrix areas with cells with different morphologies and densities (**Fig. S3,** middle and right**)**.

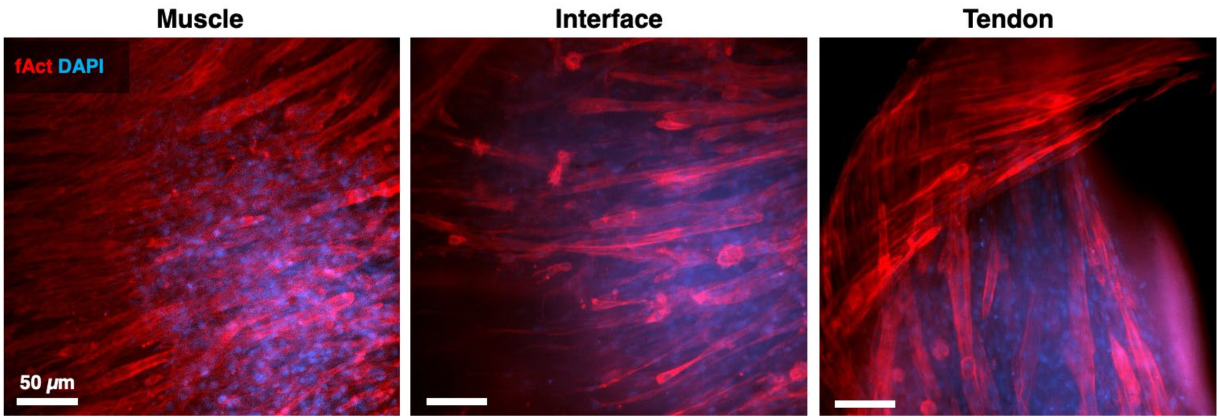

Fig. S3 | Tissue morphological analysis of MTUs with different bioink formulations.

Immuno-fluorescent staining of the MTU developed with hydrogel formulations containing gelatine, fibrinogen and Matrigel, and lacking GelMA. Cell morphology is visible in the muscle tissue, tendon-muscle interface, and tendon structure via f-Actin staining. Nuclei and F-actin are shown in blue, and red, respectively.

In summary, our optimized bioink formulations demonstrated notable conformational stability. By fine-tuning crosslinking parameters to prevent excessive tissue condensation, we achieved stabilized structures that retained key geometrical features throughout weeks of culture. Importantly, while some initial deformations were observed from the printed design to the mature tissue, this is an expected behavior for cell-laden hydrogels in a culture medium. During the early phase of tissue formation (i.e., the first two weeks), the printed constructs underwent natural tissue remodeling and hydrogel shrinkage, a well-documented phenomenon in hydrogel-based engineered tissues. Notably, unlike the other bioink components (GelMA, fibrinogen, Matrigel), which undergo physical or chemical crosslinking, the gelatin in our bioink remains non-crosslinked and instead acts as a viscosity modulator. Consequently, a portion of the gelatin gradually dissolved into the culture medium over time, potentially contributing to the acceleration of the shrinkage effect.

To illustrate the structural deformation of the constructs, we present the percentage reduction in surface area from the bioprinting time point to day 7 of the tissue maturation protocol (**Fig. S4A** and **B**). By capturing top-view images of the construct at different time points and analyzing surface area changes, we observed a reduction in surface area over time. While it is important to consider that volume loss is a three-dimensional process, surface area measurements provide a useful proxy for assessing volume changes. Thus, surface area decrease is indicative of overall volume loss, which we attribute primarily to hydrogel shrinkage and tissue remodeling. Hydrogel shrinkage is a well-documented phenomenon in tissue engineering, driven by water loss, polymer network contraction, and cell-mediated remodeling. Additionally, as cells deposit extracellular matrix and reorganize their environment, tissue remodeling may further contribute to volume reduction.

Compact MTUs and control constructs made solely of muscle matrix exhibited a surface area reduction of approximately 15–16%, indicating that condensation occurred to a similar extent in both cases. In contrast, constructs composed entirely of tendon bioink (the 'full tendon' condition) showed a lower surface area reduction (~11%). These findings suggest that the cells and matrix used for tendon printing in the MTU (namely, fibroblasts and tendon bioink polymers) do not promote hydrogel shrinkage and tissue condensation to the same extent as muscle cells and muscle bioink polymers. The MTU has a hybrid composition, in which tendon bioink occupies a smaller volume than muscle bioink. Consequently, the MTU and full-muscle control constructs exhibit a similar behavior.

Despite this initial deformation, our MTUs retained their key architectural features, ensuring proper bio-actuator formation and functionality.

Moreover, we measured the dried mass of the constructs at the end of the maturation process (culture day 15) and compared it to the initial mass. Across all tested conditions, the final mass was slightly reduced to approximately 90–95% of the initial value. This suggests some material loss, likely due to the release of polymeric components such as gelatin or non-crosslinked materials. Additionally, cell proliferation during tissue development was not sufficient to fully compensate for this loss, resulting in an overall mild mass reduction. Since no drastic differences were observed among conditions, we conclude that the matrix composition remained sufficiently stable to support tissue development and generate constructs that remained structurally intact over weeks of maturation.

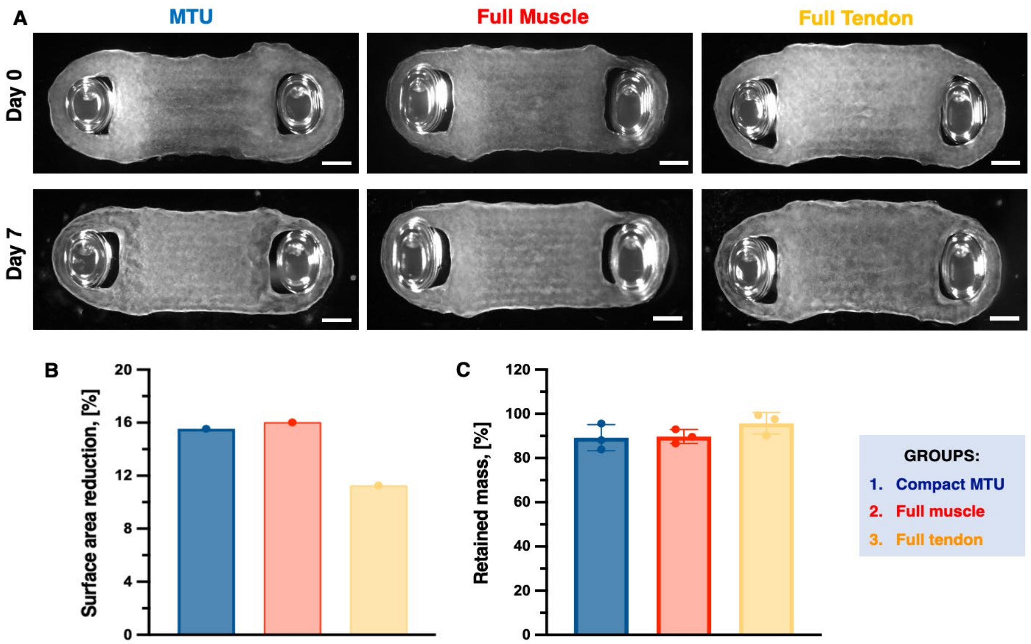

Fig. S4 | Conformational changes in the constructs.

(**A**) Representative optical pictures showing the structural deformations of MTUs from bioprinting to 7 days of tissue maturation. (**B**) Percentage surface area reduction of the constructs after 7 days of culture, as analyzed from top-view microscopic imaging. (**C**) Mass of the constructs at the end of the maturation period (15 days of culture) is expressed as a percentage fraction of the initial mass (100% corresponds to the dried mass after biofabrication). Scale bars: 1.2 mm.

Regarding long-term stability, the overall structural integrity of the designs was preserved, with key features maintained at both the millimeter and sub-millimeter scales. Notably, the shape of the anchors, bridges in the sparse designs, and interface structures with sub-millimeter invaginations remained intact over weeks and months of culture, demonstrating the robustness of our bioink formulation.

In conclusion, we observed comparable trends in volume and weight reduction for MTU constructs and control conditions made with either muscle- or tendon-bioink. The mass loss was minimal, with retained mass remaining around 90–95% of the initial value. The observed surface reduction over time was indicative of condensation effects resulting from hydrogel shrinkage and tissue remodeling, well-characterized and expected phenomena. Our bioinks were optimized to enhance stability, particularly considering the need for secure anchorage on pillars. Overall, our bioink formulations demonstrated excellent printability, enabling high printing fidelity and ensuring that construct dimensions closely matched the original design. These properties allowed the formation of mature constructs that successfully retained mm- and sub-mm-scale features throughout several weeks of culture.

S4. Characterization of the tissue maturation

To evaluate the advantages of incorporating living tendon components into bio-actuators, we compared the tissue development of MTUs to constructs consisting solely of muscle tissue and those with acellular tendons. This analysis aimed to assess how the co-culture of myoblasts and fibroblasts influences the overall progression of tissue formation. We performed confocal imaging on the constructs stained for Collagen, Myosin Heavy Chain, and f-Actin, focusing the analysis on the bio-actuator region (i.e., "central area") of the MTU (**Fig. S5**), or the anchor region (**Fig. S6**), demonstrating the different distribution of the two tissue markers.

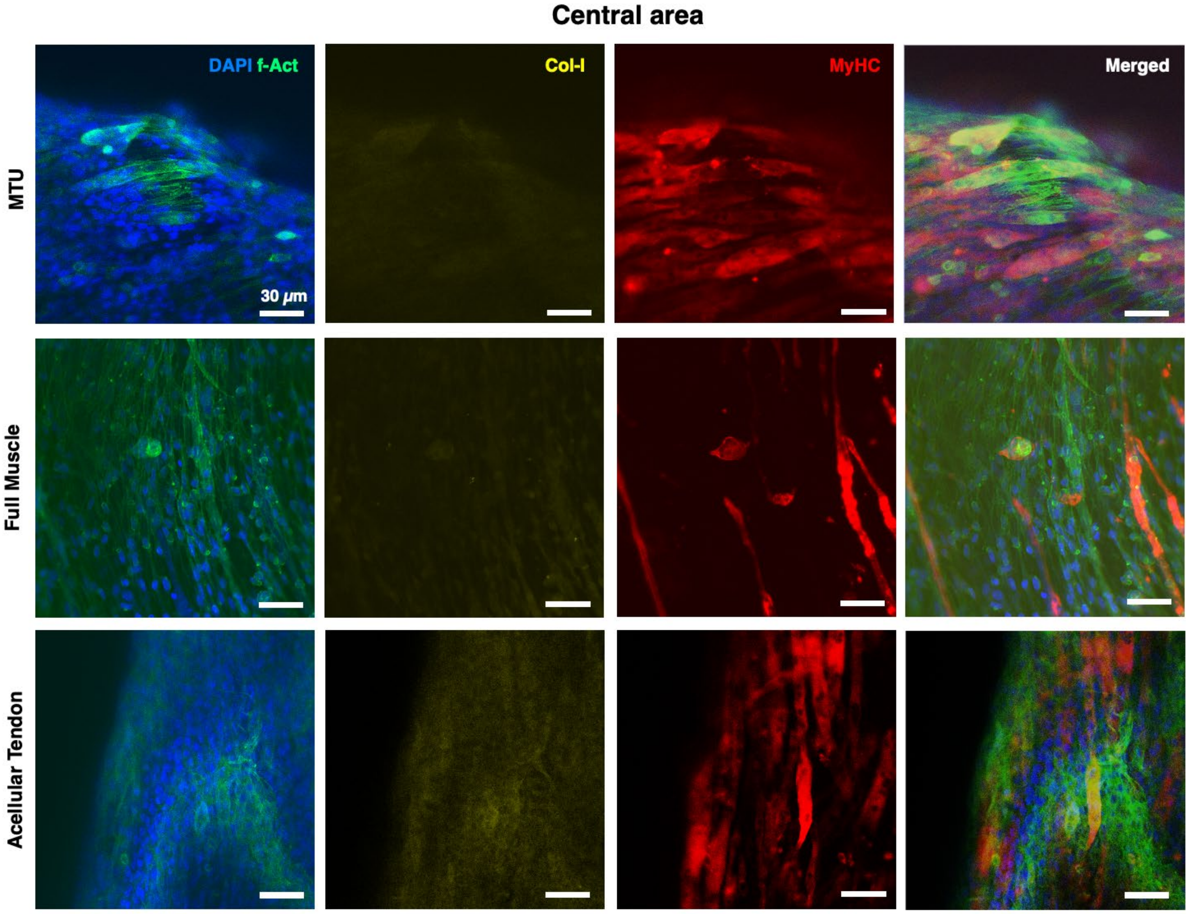

Fig. S5 | Immunostaining of the constructs' central area.

Confocal imaging of the central area of the constructs (day 13) stained for f-Actin (green), DAPI (blue), Collagen-I (yellow), and MyHC (red), in which skeletal muscle tissue is expected to grow.

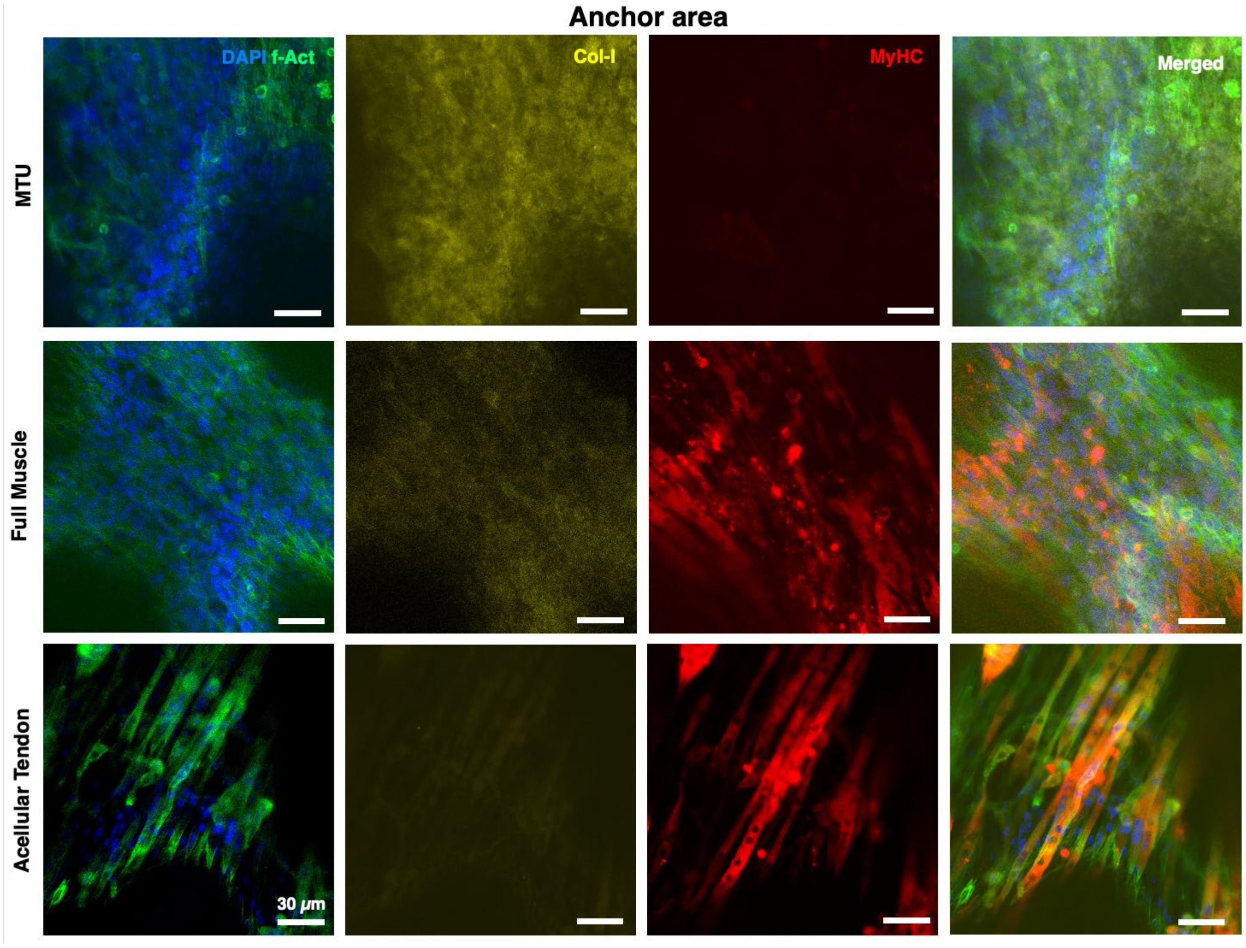

Fig. S6 | Immunostaining of the constructs' anchor area.

Confocal imaging of the anchor area of the constructs (day 13) stained for f-Actin (green), DAPI (blue), Collagen-I (yellow), and MyHC (red).

NIH 3T3 fibroblasts typically produce substantial amounts of collagen.(*27*) As key contributors to extracellular matrix (ECM) production and deposition, fibroblasts secrete high levels of collagen, primarily types I and III, to support connective tissue integrity. When constructs were printed without fibroblasts in the anchor regions (acellular tendons), myoblasts migrated into these spaces, leading to a reduction in overall collagen levels (**Figure S7, left**). Fibroblasts exhibit strong vimentin expression, whereas differentiated muscle cells do not. As myotubes mature, vimentin expression diminishes and eventually disappears, while desmin expression persists, becoming the primary intermediate filament in mature muscle fibers. Consequently, vimentin staining serves as a useful marker for distinguishing fibroblasts from myoblasts or myotubes. Compared to collagen staining, vimentin staining provides a clearer contrast against the background, as collagen staining can result in residual fluorescence, potentially due to non-specific interactions with the gelatin in the matrix (**Figure S7, right**). Vimentin staining was particularly valuable in assessing the interface between cell compartments. As shown in Figure 5, vimentin is present in the myotendinous unit (MTU) but is absent in both pure muscle controls and acellular tendons.

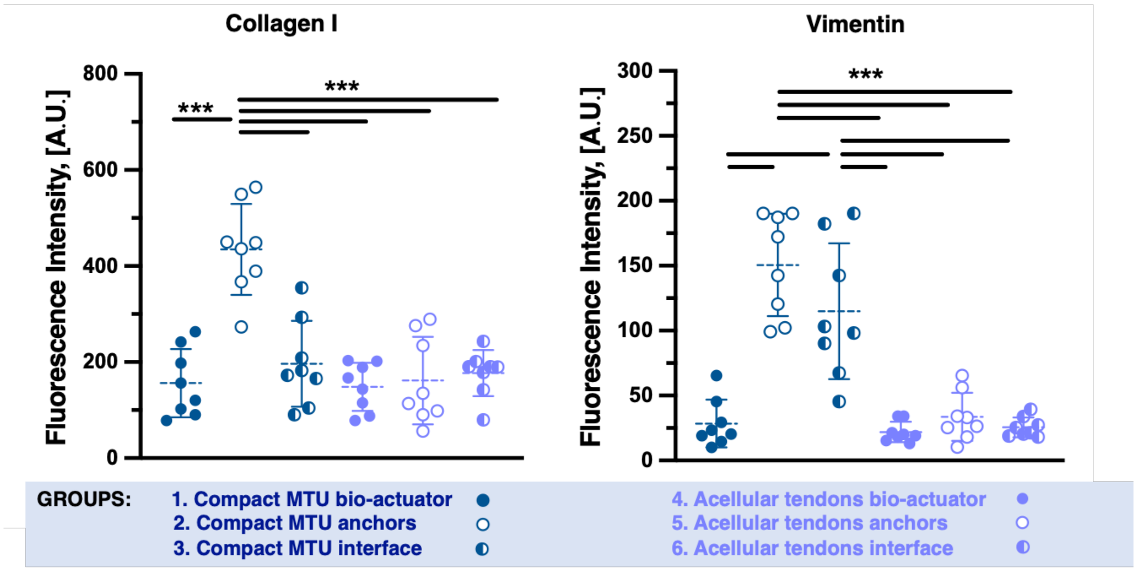

Fig. S7 | Quantitative assessment of collagen and vimentin expression.

Fluorescent signal in arbitrary units of fluorescence (a.u.) from different regions (anchor, bio-actuator, and interfaces) of MTU and acellular tendon controls. In the left picture, p-values are: 1.09×10^{-5} (groups: 1 and 2), 0.339 (1 and 3), 0.799 (1 and 4), 0.898 (1 and 5), 0.264 (1 and 6), 0.0001 (2 and 3), 2.67×10^{-6} (2 and 4), 4.027×10^{-5} (2 and 5), 7.844×10^{-6} (2 and 6), 0.206 (3 and 4), 0.455 (3 and 5), 0.596 (3 and 6), 0.722 (4 and 5), 0.264 (4 and 6), 0.682 (5 and 6). In the right picture, p-values are: 1.509×10^{-6} (1 and 2), 5.8×10^{-4} (1 and 3), 0.376 (1 and 4), 0.583 (1 and 5), 0.689 (1 and 6), 0.146 (2 and 3), 3.262×10^{-7} (2 and 4), 2.561×10^{-6} (2 and 5), 4.501×10^{-7}(2 and 6), 0.0002 (3 and 4), 0.00098 (3 and 5), 2.8×10^{-4} (3 and 6), 0.126 (4 and 5), 0.372 (4 and 6), 0.277 (5 and 6). Statistical significance is expressed as *** for $p < 0.001$.

S5. Interface design and architectural retention

We connected the two tissues by printing layers with different bioink arrangements, alternating between interwoven lines and aligned turns (Fig. 3). Microscopic imaging on H&E-stained tissue sections reveals that on the surface of the construct, two different cell populations were present at the interface area (**Fig. S8A-B**). When cutting sections at approximately 1 mm of tissue depth, we found mostly empty matrices (**Fig. S8C**). At such tissue depth, cells typically do not survive due to the limited oxygen and nutrient perfusion. However, deep tissue sectioning revealed textures corresponding to the different matrix compositions, aiding in the visual assessment of the multi-material assembly. The MTUs exhibited an inter-tissue interface characterized by interdigitations, with an interdigitation radius (depth of the interlocking zones, indicated by the black dashed arrow) of approximately 0.8 mm. Given an average construct length of 15 mm, this suggests that the tissues are interpenetrated by about 5% of the total MTU length.

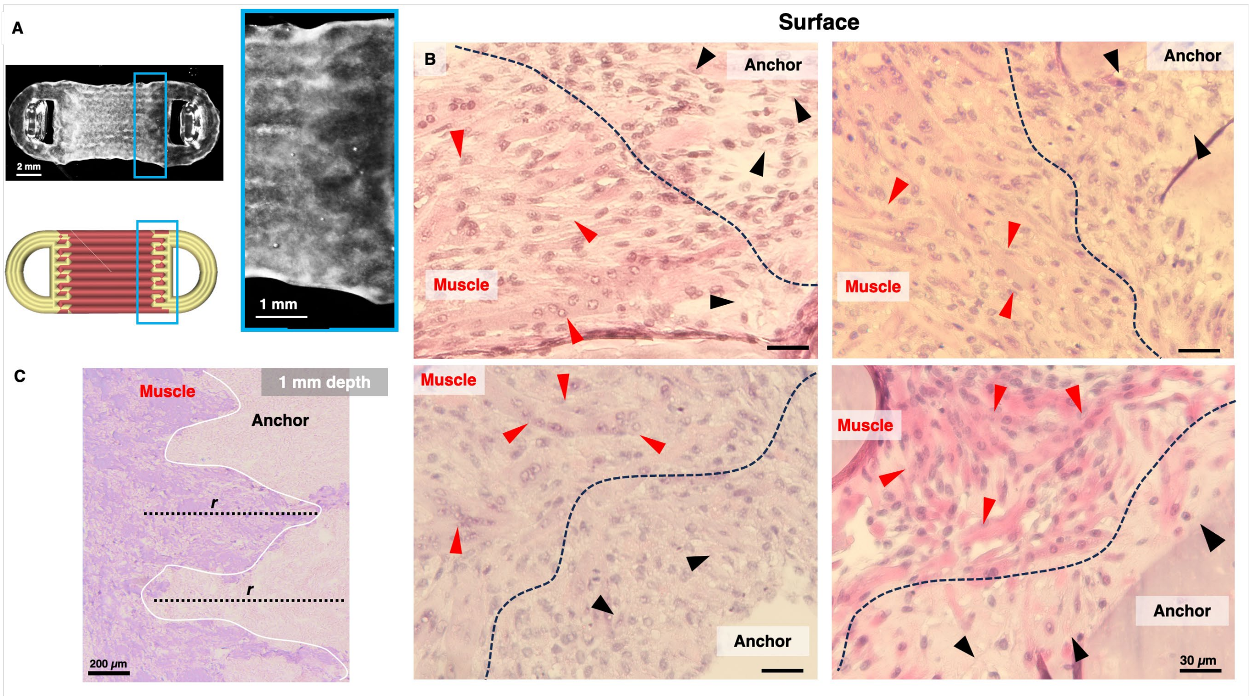

Fig. S8 | Structure of the muscle-tendon interface.

(**A**) Stereoscopic picture and CAD of the compact MTU with a magnified image detail of the muscle and tendon tissue interface, highlighting texture differences between the tissues. Cyan boxes indicate the interface area. H&E Staining of the MTU interface area from the surface (**B**) or deep tissue, at 1 mm of tissue depth circa (**C**). Red and black arrows indicate myotubes and fibroblast-shaped cells, respectively. The white line indicates the contour of the interface structure, highlighting the architecture of two matrices combined in an interlocking design. Black dashed line indicates the length of the invagination radius (*r*).

This interlocking arrangement enhanced cohesion between the printed materials, demonstrating that the combination of interdigitated design and bioink mixing zones at the muscle-tendon interface facilitated the successful integration of the developing tissues.

S6. Considerations on the choice of the MTU design

The mechanical properties of the MTU constructs are expected to differ between the compact and sparse designs due to variations in their structural configurations. Understanding these differences is useful to operate an accurate selection for tasks-specific designs. Here, we briefly propose a few considerations on the mechanics of the MTUs as realized with different designs.

In the compact design, the MTU tissue is more continuous and homogeneous, which enhances its structural integrity and provides a more rigid framework. This continuous tissue network facilitates a more even distribution of forces, resulting in increased stiffness throughout the construct.

Conversely, the sparse design incorporates parallel segments or bridges in the central region, creating gaps between the tissue that reduce the overall cross-sectional area responsible for bearing load. As a result, the sparse design contains less material that resists deformation, leading to a lower stiffness compared to the compact configuration.

The spacing between the parallel segments or bridges (i.e., the line spacing) further affects this stiffness: larger spacing reduces the contact area between the tissue and surrounding matrix, further diminishing the construct's rigidity. Consequently, the sparse design is more flexible and able to undergo greater deformation under applied loads, while the compact design is stiffer and more resistant to deformation. The relationship between line spacing and stiffness is a key aspect of MTU design, enabling the customization of the mechanical properties of the constructs to meet the specific functional requirements of the bio-actuator.

Moreover, it is worth mentioning that, although the constructs were accurately printed with initially separate segments, during the culture period, these segments tended to merge at contact points, particularly when they were nearby and moved within the medium (**Fig. S9**). Fusion between segments predominantly occurred in the central two bridges, likely due to their more constrained positioning. In contrast, the external segments might experience greater freedom of movement due to their lateral position. Fusion could occur to varying extents, ranging from 25% to 85% of the bridge length.

Tissue development is inherently complex and prone to such occurrences. We hypothesize that fusion events between adjacent bridges could cause the sparse design construct to resemble the compact configuration. This fusion is expected to influence kinetic energy variations. As shown in Fig. 6, the kinetic energy measured for the sparse design is higher than that of the compact designs, albeit with significant variability. This variability may be partially attributed to the differing degrees of fusion events.

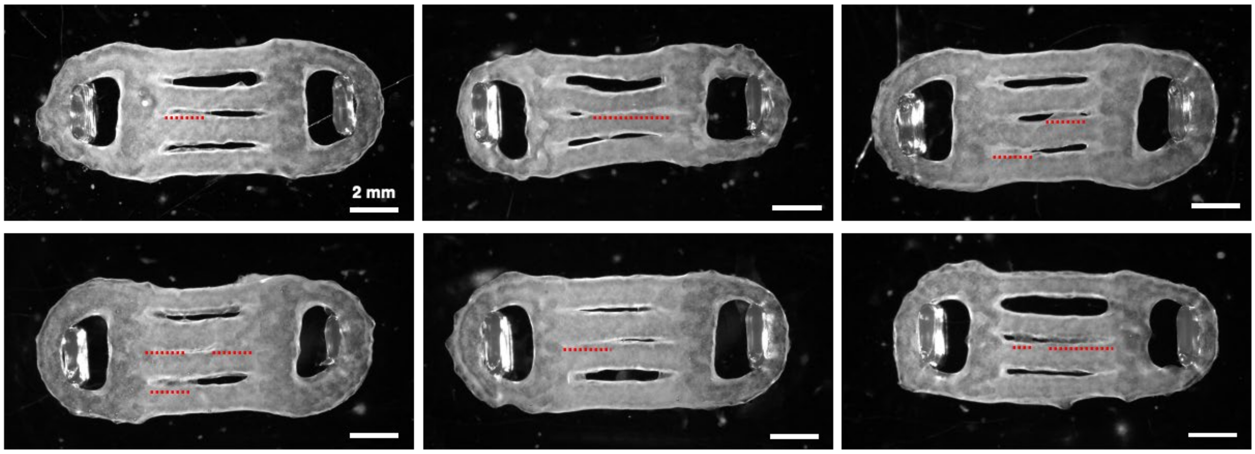

Fig. S9 | Events of bridge fusion during tissue development.

Stereoscopic imaging of representative sparse MTUs in which events of bridge fusion occurred. Red dashed lines highlighted the extension of the tissue fusion.

S7. Electrical stimulation of the MTU for dynamic analysis

We conducted bio-actuation tests on the MTU constructs and their controls by applying electrical stimulation while simultaneously capturing imaging data. This approach allowed us to record the functional response of the engineered tissue and monitor the contractile behavior in real time. In the recorded videos, we performed the dynamic analysis. The constructs were placed in an electrical stimulation bath (six-well plate well or petri dish), anchored to two soft, printed silicon pillars (**Fig. S10**). Electrodes were fabricated from graphene bars connected through metallic wires to a function generator. Videos were recorded on a stereoscopic. An optical microscope was used to image micromotions at a higher magnification and report them as lateral side motions in the constructs (see **Section S11**, *Displacement of the MTU in untethered configuration)*. The setup is shown in the picture below.

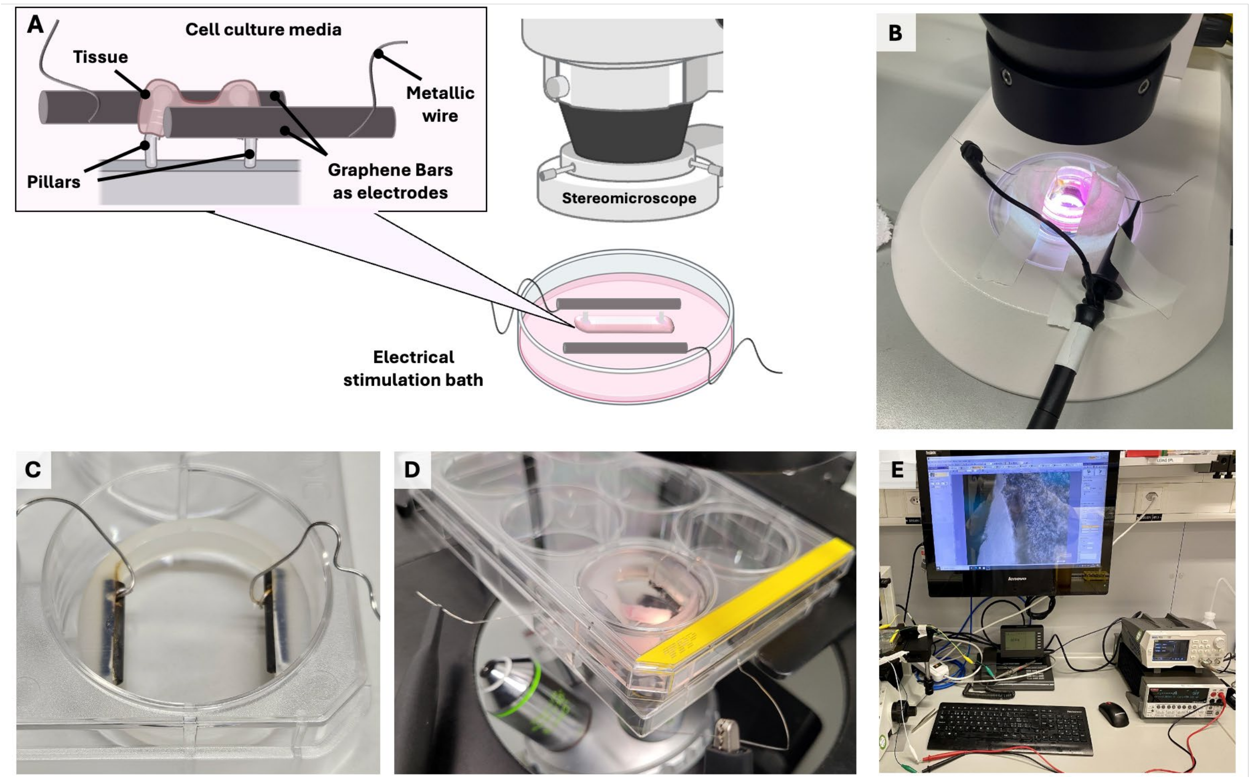

Fig. S10 | Electrical stimulation setup.

(**A**) The constructs sit at the bottom of a chamber filled with cell culture media, while being anchored to two soft pillars between two graphene bars used as electrodes. Imaging devices (stereomicroscope or optical microscope) were properly positioned to acquire top images of the constructs. Created in BioRender. Filippi, M. (2025) https://BioRender.com/0rrv0rk. (**B**) Setup mounted on a petri dish under the stereomicroscope. (**C**) The holder with the graphene bar electrodes inserted in a 6-well plate. (**D**) The stimulation chamber is mounted under the optical microscope. (**E**) The stimulation chamber is connected to the function generator and real-time imaging is recorded and shown on a screen.

S8. Stiffness gradient and elastic behavior in the MTU

We performed micro-indentation tests using the FT-MTA02 micromechanical test station (FemtoTools AG, Baar, Switzerland) with a spherical cubic zirconia indenter (R = 100 µm). Two conditions were tested: (1) samples immersed in PBS (**Fig. S11A**) and (2) samples dried in air for 10 minutes (**Fig. S11B-C**). To ensure stable and well-defined mechanical boundary conditions, samples were fixed with cyanoacrylate glue. For each condition, nine indentation curves were recorded for both tendon and muscle. Young's modulus was determined from each curve using a Hertzian contact model. An indentation depth of 10 µm (10% of the radius) was consistently used for all tests, to ensure that the Hertzian contact formula provides an acceptable approximation.

The force-displacement curves revealed a strong difference in stiffness and nonlinearity, confirming the higher stiffness of the tendon region (i.e., anchors). The variability in the tendon's response likely stems from surface topography effects in a stiffer material.

The slight difference between the *E* values obtained in wet versus dried configurations can be attributed to various factors. For the eSMT, this difference is consistent with the expected effects of drying, where tissues typically become stiffer due to water loss. For our engineered tendon tissue, the wet configuration shows a higher *E,* possibly because of the higher collagen content of the matrix, which causes that hydration likely optimizes fiber alignment and tension within the structure, whereas drying might compromise the internal organization, leading to a reduced modulus.

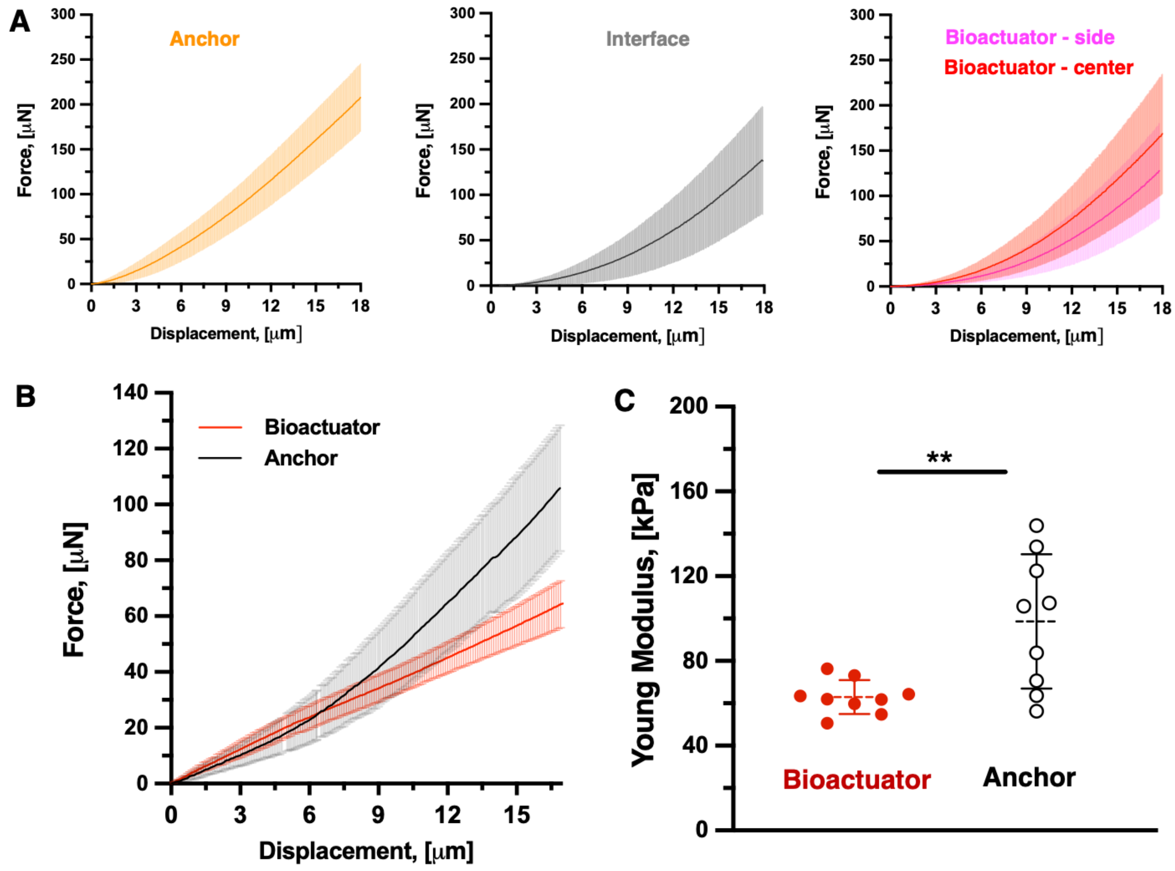

Fig. S11 | Different stiffness in anchors and bioactuator regions in air-dried MTUs.

The force-displacement curve of the wet (**A**) and dried (**B**) MTU sample indicated a strong difference in stiffness among tested areas. For wet samples, curve are shown for anchor, interface and bioactuator's regions, with muscle tested in the lateral and central position. (**C**) Young moduli are calculated from indentation curves for both the bioactuator (i.e., muscle tissue) and anchor (i.e., tendon tissue) areas. *P*-value: 0.005. Statistical significance is expressed as ** for $p < 0.01$.

To assess the elastic behavior of the MTUs, the samples were tested using a uniaxial tensile testing machine (Instron 5942, Instron, Norwood, MA, USA) at room temperature. Before testing, the constructs were washed, and their extremities were carefully attached to two pieces of paper using a strong tissue adhesive. The papers were then folded around the constructs, which were precisely positioned in a vertical orientation within the sample holder of the tensile machine. The samples underwent cyclic stress testing with variable frequencies (0.1 to 5 Hz) and applied displacements ranging from 0.2 to 0.8 mm (**Fig. S12-S13**). The range of displacements and frequencies explored for these tests matched the deformations the MTU undergoes when electrically stimulated for actuation onto the pillars. As the tensile load was increased and decreased cyclically, the material's deformation corresponded directly to the stress variations without exhibiting permanent deformation or hysteresis, indicating that the force response consistently followed the applied stress pathway in a reversible and repeatable manner, thereby confirming the material's elastic behavior.

The distinct mechanical properties of compact and sparse MTUs are a direct result of their contrasting structural configurations. From the tensile tests, we estimated the *E* for the compact and sparse MTU, being ~ 61.5 and 27.2 kPa, respectively. For our silicone pillars, we measured an *E* of 206 kPa, which falls within the stiffness range expected for printable resins with elastomeric properties, formulated with a low base-to-curing agent ratio and cured at low temperatures (~100-300 kPa) (**Fig. S12B)**.(*43,44*)

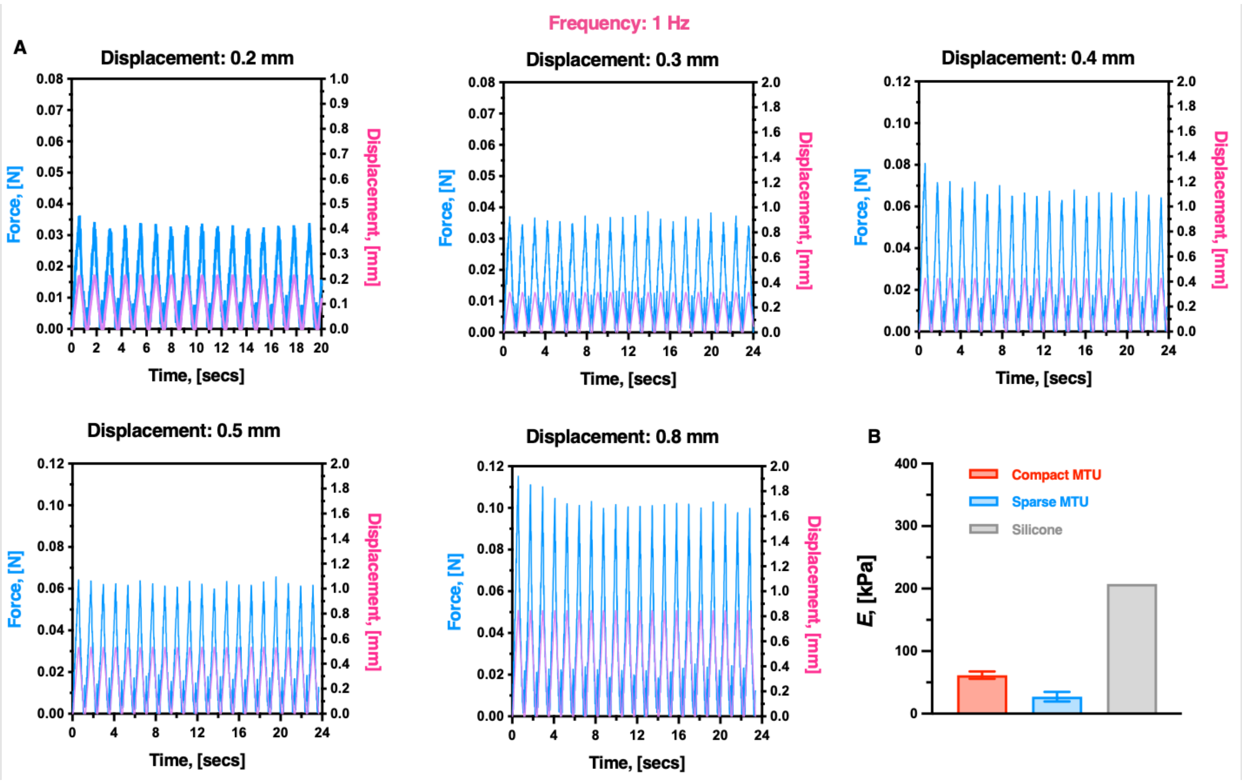

Fig. S12 | Cyclic tensile test of the MTU with different strains.

(**A**) Time-dependent force variation of the MTU exposed to cyclic tensile tests with variable imposed displacements, ranging from 0.2 to 0.8 mm, applied at 1 Hz. (**B**) Young's modulus calculated from tensile testing of the compact and sparse MTUs, and silicone used to create synthetic pillars.

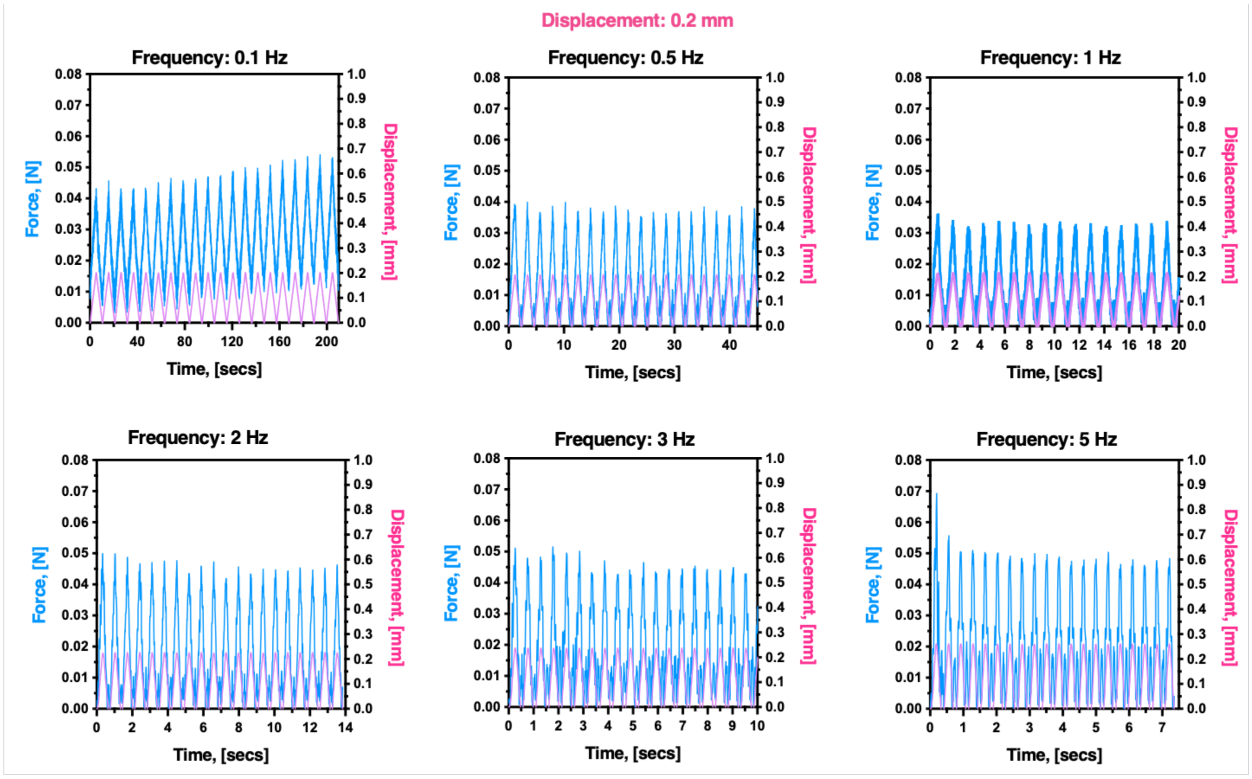

Fig. S13 | Cyclic tensile test of the MTU with different frequencies.

Time-dependent force variation of the MTU exposed to cyclic tensile tests with applied strain of 0.2 mm at variable frequencies, ranging from 0.1 to 5 Hz.

S9. Force Measurements in the MTU

To evaluate the functional performance of our muscle-tendon unit (MTU) constructs, we directly measured their force output using the Aurora Scientific 404C force transducer system and the 302C linear positioner on day 15 of culture (**Fig. S14**). The MTUs and control constructs were securely positioned between the transducer lever arm and a fixed post mounted to a linear piezoresistive positioner. Metal wires, bent into hooks, were used to attach the constructs to the system, either anchoring to the structural supports of the MTUs or the rings in the case of ring-shaped control constructs. To preserve tissue viability, all measurements were conducted in a cell culture medium. Electrical pulses (1 V mm^{-1}, 10 ms pulse duration, 1 Hz frequency) were applied via field electrodes to induce contractions, while force output was recorded simultaneously. Video recordings were co-acquired to correlate tissue motion with force generation. Data acquisition and analysis were performed using the Aurora Scientific 600A software, providing a detailed

characterization of the contractile functionality of the engineered constructs.

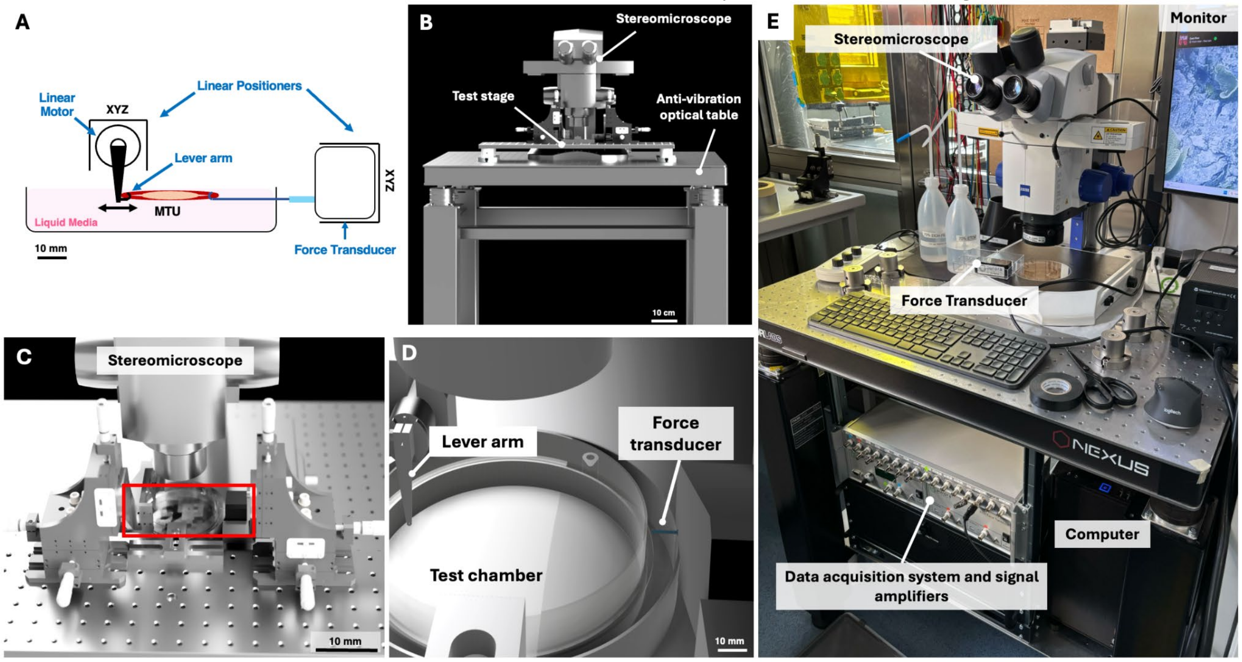

Fig. S14 | Force measurement setup.

(**A**) Schematics of the working principle of the force measurement setup. (**B**) Rendering of the force transducer's setup showing the test stage and stereomicroscope mounted on an optical table. Zoom-in of the rendering picture showing: the linear positioners for the lever arm (left side of the picture) and the force transducer (right side of the picture), mounted below the stereomicroscope (**C**); and the test chamber (**D**) with the force transducer working through a sensitive small bar-shape sensing element. (**E**) Picture of our measurement setup.

S10. Pillar-MTU interaction during actuation

Pillar displacement was quantified using image subtraction analysis performed in Fiji (ImageJ). This method allowed for the precise detection of structural shifts by comparing images captured in different states. By analyzing the differences between these images, we were able to measure and quantify the extent of pillar deformation. In **Fig. S15**, we show representative pictures of the compact MTU during contracted and relaxed states (**Fig. S15A**) and the result of image subtraction analysis (**Fig. S15B**). In **Fig. S15A**, we drew dashed yellow lines to connect specific features between the two images and illustrate the construct's displacement. Their slight deviation from vertical (rotational angle: 2° circa) suggests minimal movement during the actuator's contraction and expansion phases.

Due to differences in force production, the actuated MTUs exerted greater stresses on the pillars of the maturation platforms, resulting in larger deformations. However, the increases in force and pillar deformation between the muscle controls and MTUs were approximately 100% and 200%, respectively. Since pillar deformation did not scale linearly with the applied force, other factors

may influence the dynamic behavior of the MTUs. One such factor could be the progressive changes in the mechanical properties of the interface, driven by fibroblast-populated anchors, which may enhance force transmission to the PDMS pillars.

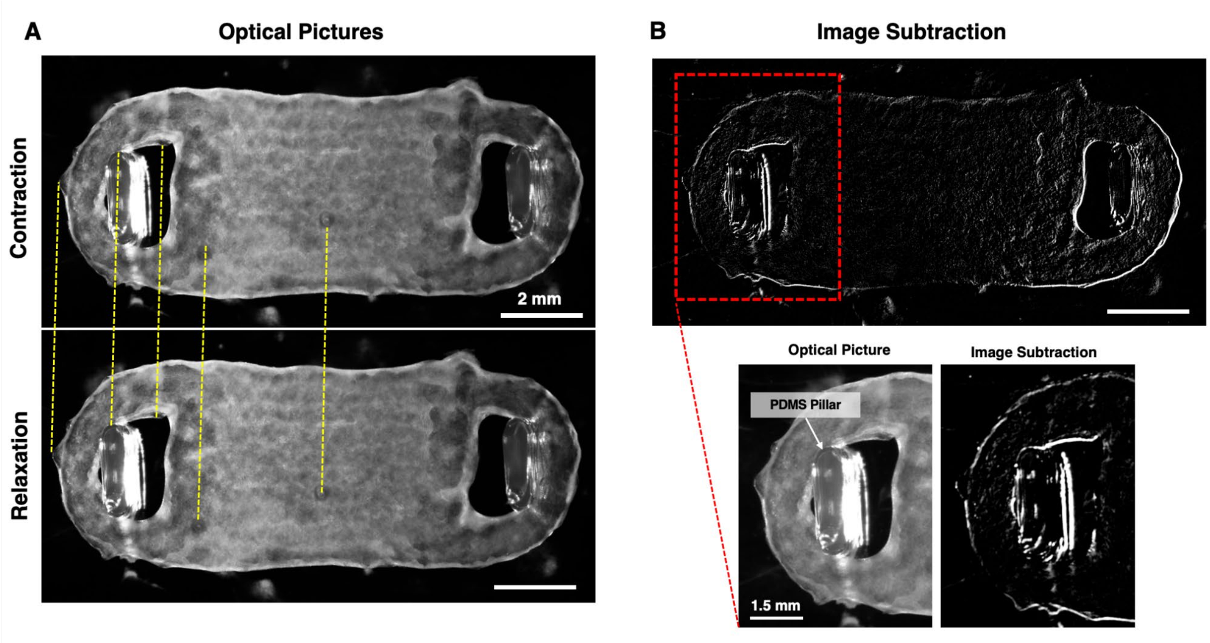

Fig. S15 | MTU-pillar interaction under actuation.

(**A**) Representative microscopic images of the compact MTU in its contracted (top) and relaxed (bottom) states. Dashed yellow lines are drawn to connect specific features between the two images, highlighting the displacement of the construct. The lines are not perfectly vertical, indicating the minimal displacements occurring during the contraction and expansion phases of the actuator. (**B**) Representative image subtraction between the contracted and relaxed states (top), along with a magnified detail of the MTU-pillar interaction.

S11. Long-term contractility and displacement in untethered configurations

In this study, we demonstrated that MTUs and controls respond differently to actuation stimuli, exhibiting distinct motility and deformation ranges (**Fig. S16A**). The contractile abilities of our MTU emerged early in tissue development with the onset of spontaneous contractions on day 5 (**Movie S5, Fig. S16**), and the responsiveness to electrical stimulation persisted for several weeks beyond the typical tissue development period (2 weeks). Indeed, we observed that our MTUs remained viable and contractile for approximately three months, significantly exceeding the durability of similar muscle tissue-based bioactuators, which typically last around 2–3 weeks. When mounted on their maturation platforms, the active tension strain of MTUs resulted in minimal deformations with lateral displacements in the range of tens of µm (**Fig. S16B**). The ability to induce lateral displacement in response to contraction increased up to 15 days. No statistically significant differences were observed between the behavior at 15, 30, and 90 days, suggesting that contractile abilities may stabilize by the end of the tissue maturation protocol.

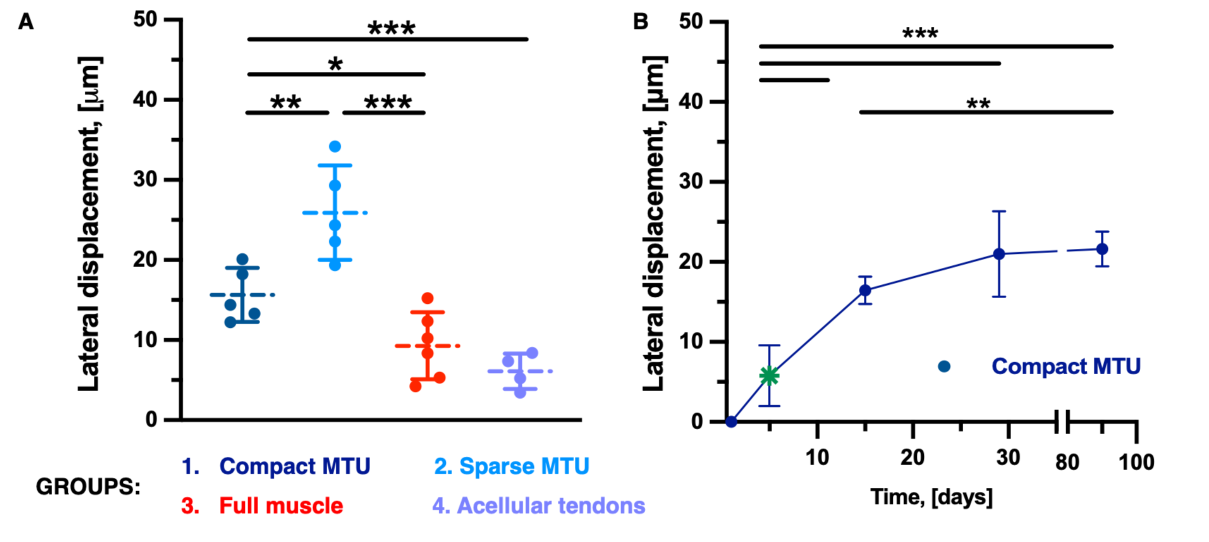

Fig. S16 | Durable contractility over time.

(**A**) Lateral displacement calculated from image subtraction analysis, quantifying the motility of the constructs after 15 days of culture, corresponding to the end of the tissue development period. (**B**) Lateral displacement of MTU constructs at different time points over a culture period of up to three months. The point indicated as a green asterisk (day 5) represents the displacement induced by spontaneous contractions. In A, P-values are: 0.009 (groups: 1 and 2), 0.046 (1 and 3), 0.002 (1 and 4), 3.9×10^{-4} (2 and 3), 4.0×10^{-4} (2 and 4), and 0.206 (3 and 4). In B, P-values are: 4.4×10^{-4} (points: 1 and 2), 8.4×10^{-4} (1 and 3), 4.8×10^{-5} (1 and 4), 0.107 (2 and 3), 0.003 (2 and 4), and 0.822 (3 and 4). Statistical significance is expressed as * for $P < 0.05$, ** for $P < 0.01$, and *** for $P < 0.001$.

Understanding the dynamic behavior in fluids is essential for designing bio-actuators capable of efficient, biomimetic movement in engineered systems. To assess the dynamic behavior of our MTU under different configurations, we stimulated the muscle's contraction while keeping the MTU anchored onto both the two pillars (tethered configuration), with only one pillar attached to the construct's hole (one-side tethered), or without any pillar inserted (untethered) (**Fig. S17**). As seen in prior experiments, the actuation of the MTU under anchorage with both pillars resulted in cyclic uniaxial contraction and expansions, causing PDMS-pillars bending.

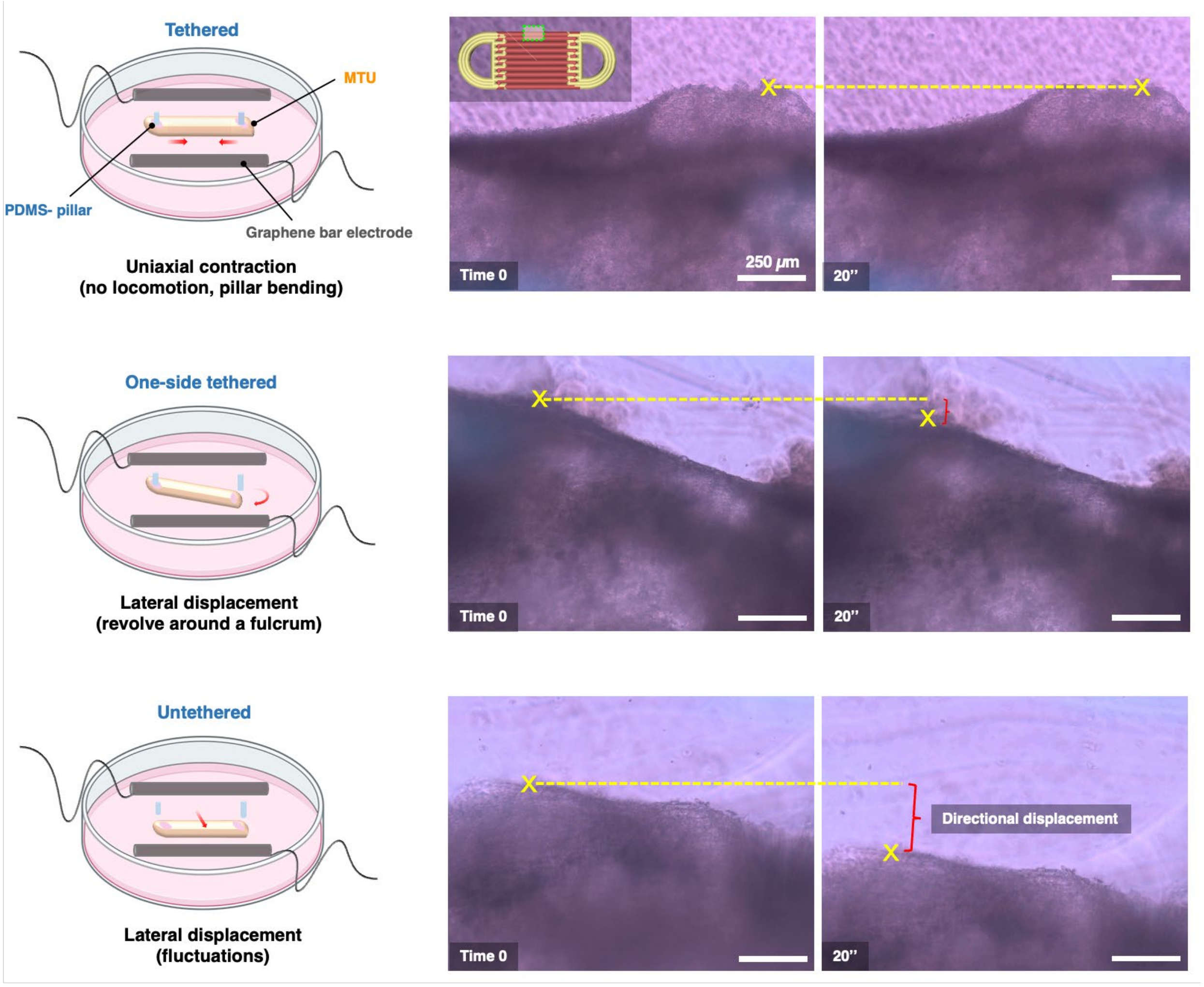

Fig. S17 | MTU displacement in tethered or untethered configurations.

Left: schemes of the different configurations to study the dynamic response of MTU when experiencing constraining anchorage or not: anchored onto both the two pillars (tethered configuration; top), or only one pillar was attached to the construct's hole (one side-tethered; middle), or finally MTU was left without any pillar inserted (untethered; bottom). Right: optical pictures of a detail of the MTU constructs that were used to image its displacement. The inset shows the imaging point on the lateral side of the constructs. Yellow signs indicate the position of features of reference; yellow dashed lines connect those features to show the directional displacement, and red lines illustrate the displacement. Created in BioRender. Filippi, M. (2025) https://BioRender.com/cepz55w.

However, when actuating one-side tethered and untethered MTUs, we observed lateral displacement (**S17, middle and bottom**). We assumed that a uniaxial actuator anchored on one side and immersed in a liquid medium can exhibit rotational motion around a fulcrum, leading to lateral displacement under certain conditions. If the contraction and expansion cycles are not

perfectly symmetrical or the actuator has an asymmetric geometry, a rotational moment may develop around the anchor point, causing pivot-like movement. Additionally, interactions with the surrounding fluid play a crucial role, as variations in drag forces can create an imbalance that drives lateral motion. The flexibility of the anchoring system can further amplify this effect, allowing slight deformations or elastic bending to contribute to curved trajectories rather than purely axial motion. In general, if the fulcrum serves as a partial constraint rather than a fixed point, contractions may induce rotational displacement instead of simple linear shortening. Our MTU was allowed to rotate around the pillar, as the hole in the anchor was sufficiently large and there was no tissue-pillar adhesion mechanism. Similar mechanisms are observed in biohybrid actuators and soft robotic systems, where asymmetric actuation patterns enable controlled locomotion in fluid environments.(*21, 27*)

Second, a perfectly symmetrical uniaxial actuator placed in a liquid medium without constraints should, in theory, remain in its original position, as its contraction and expansion cycles generate equal and opposite forces. However, if movement is observed, several factors could be responsible. Small asymmetries in force generation or timing of contraction across the actuator could introduce an imbalance, leading to directional motion. Additionally, interactions with the surrounding fluid, such as variations in drag forces or vortex formation, may create unequal resistance during expansion and contraction, resulting in net displacement. Structural factors, such as slight deformations or elasticity in the actuator, could also alter force transmission and disrupt perfect symmetry. Furthermore, if the contraction phase is faster or stronger than expansion, a nonlinear force imbalance may emerge, gradually propelling the actuator. Even in a theoretically ideal setup, external perturbations such as residual currents, uneven fluid properties, or minor imperfections in fabrication could introduce tiny biases that accumulate over multiple actuation cycles. Therefore, any observed displacement likely stems from biomechanical, hydrodynamic, and structural nonlinearities affecting the actuator's motion.

S12. Engineering biohybrid systems to mimic anatomical structures

The MTU developed in our work can be used to build dynamic models of anatomical structures of similar size. For example, here we built a multicomponent system that mimics the middle ear structure, as the MTU's size closely aligns with that of the stapedius muscle and related tendon (**Fig. 1**). We printed a synthetic stapes-like structure with PDMS. We exposed it to vibrations generated from a custom-made setup based on an imbalance fan. Vibrations were collected and transferred through a wire to the stapes structure. The MTU system was mounted on a two-pillar system, in which one of the pillars was represented by the stapes-like structure, and exposed to the induced vibration. The MTU was actuated in the effort of generating counteracting forces on the stapes and eventually stabilizing it, thus mimicking the function of the stapedius muscle (**Fig. S18**).

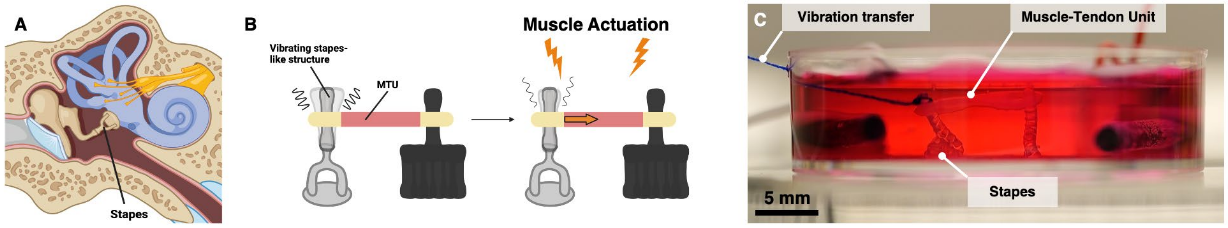

Fig. S18 | Modeling the anatomical dynamic structure of the middle ear.

We showcased the utility of our MTU to replicate the segment stapedial muscle-stapes in the middle ear, as an example of a soft-hard hybrid anatomical system in our body. (**A**) Scheme of the middle ear structure (realized with BioRender). (**B**) Schematics of the replicated system in vitro, which include the MTU as a connecting element between the vibrating system and a static pillar. Created in BioRender. Filippi, M. (2025) https://BioRender.com/3495z8y. (**C**) Optical picture of the in vitro dynamic model based on the developed MTU.

S13. Different designs for the MTUs and their maturation templates

We explored MTU design variations. A biomimetic myotendinous tissue model was developed by encapsulating engineered skeletal muscle tissue within an engineered tendon layer, mimicking the hierarchical structure of the natural myotendinous junction. This core-shell configuration was designed to replicate muscle and tendon tissue's structural and functional integration.

A core-shell design can be considered biomimetic for engineering muscle-tendon tissues because it mimics the natural hierarchical organization of these tissues. In vivo, skeletal muscle fibers are encased in connective tissue layers (such as the endomysium, perimysium, and epimysium), which transition into the tendon at the myotendinous junction (MTJ). The core-shell approach replicates this arrangement by embedding engineered muscle tissue within an outer layer of engineered tendon-like material, resembling the gradual structural and mechanical transition from muscle to tendon. This design could facilitate graded stiffness across the interface, improving mechanical integration and functional energy transfer, much like the native MTJ.

Three fabrication methods were assessed, combining multi-material extrusion-based 3D bioprinting with manual deposition techniques. Two distinct bioinks, tailored with varying stiffness properties, were formulated: one seeded with C2C12 myoblasts to form the muscle

component, and the other containing NIH/3T3 fibroblasts for the tendon component **Fig. S19**.

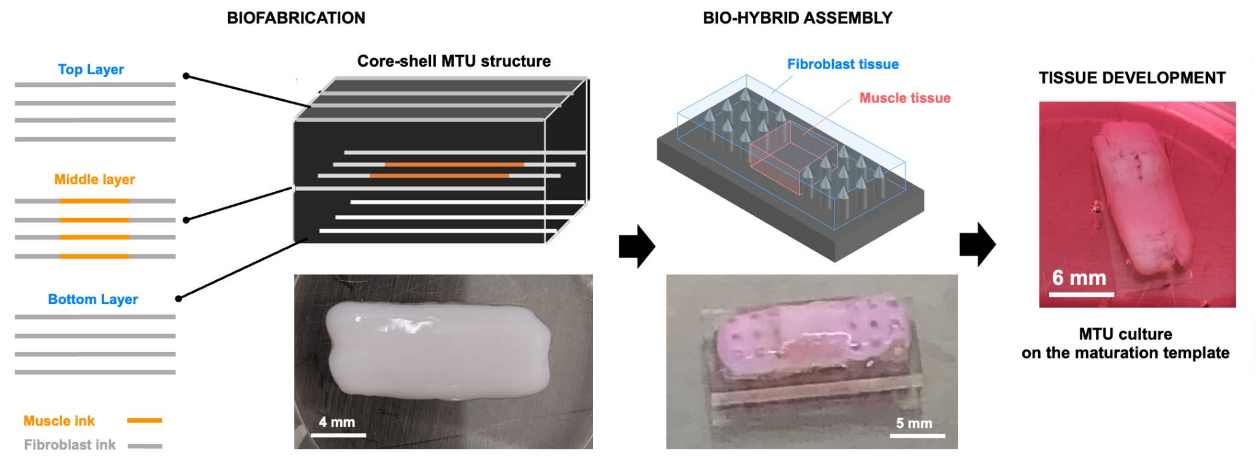

Fig. S19 | Core-shell design of the MTU.

We explored new designs for the MTU, like the core-shell design, which mimics the natural geometry of the muscle-tendon systems, in which the muscle tissue is encapsulated in the tendon. The design was realized by overlaying three layers to realize a 1.5 cm-long construct (left; an optical picture of the construct after biofabrication in top view). Constructs were mounted on maturation templates for the application of passive tensioning (middle) and then cultured for tissue development (right).

The muscle bioink used for the core of the construct was composed of 30% Matrigel (v/v), 1.6 mg/mL Collagen type I (Lifeink 200 Collagen Bioink, pH Neutral) and 5% GelMA and 7×10^6/ml C2C12 myoblast cells, while the shell part (tendon-like tissue) was composed of 25 mg/mL of Collagen type I (Lifeink 200 Collagen Bioink, pH Neutral) and 7×10^6/ml NIH/T3T fibroblasts. The constructs were created using multimaterial extrusion-based 3D bioprinting on a CELLINK BIO X6 printer, employing two depositing nozzles and CELLINK-compatible cartridges with 22-gauge tapered tips. Separate printheads were utilized for each bioink. Before bioprinting, manufacturing parameters were assessed through manual deposition. For the muscle bioink, extrusion was performed at 45-65 kPa pressure with a printing speed of 8 mm/s, crosslinked at 405 nm UV light for 15 seconds per layer, and maintained at 4-10°C. The tendon bioink was deposited at a pressure of 6-8 kPa, a printing speed of 4 mm/s, and at room temperature (**Fig. S20**). The same bioinks were used to manually fabricate control constructs to compare with the bioprinted constructs. Constructs were prepared by manually depositing the hydrogels within a stereolithography (SLA) 3D-printed mold made with clear resin (Formlabs Form 3+). Layers of bioink were sequentially deposited: a tendon bioink base layer was spread, followed by the muscle bioink as the intermediate layer in the core-shell design, and capped with the tendon bioink. The muscle bioink layer was cured under 405 nm UV light for 40 seconds. The MTUs were incubated for 1 hour to initiate collagen polymerization, after which a growth medium (GM) containing 10% Fetal Bovine Serum (FBS), 1% Penicillin-Streptomycin, and 89% DMEM (high glucose, pyruvate) was introduced. Post-deposition, constructs were anchored to a pillar system for proper

passive mechanical training. PDMS anchoring systems were coated with fibronectin by immersion in a 10 µg/ml fibronectin solution in PBS for 5 hours before use.

After fabrication, the constructs retained cohesion and adhered to the intended design, allowing successful transfer onto anchoring systems, used for proper tissue maturation under mechanical stress. Following a 5-hour incubation, the MTUs were mounted onto anchoring systems by piercing the tendon section through the pillars. This setup ensured that the tendinous section was in contact with the pillars while leaving the muscle portion free for contraction.

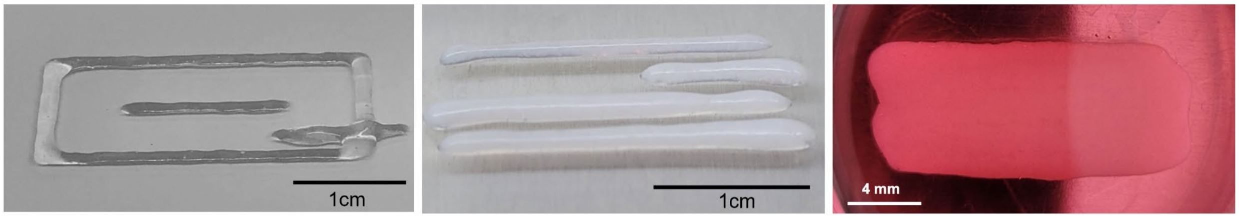

Fig. S20 | Printing optimization of the core-shell design of the MTU.

Optical picture of a printing test for the muscle (left) and the fibroblast-bioink (middle), which were optimally printed at a pressure of 45 and 6 kPa and a speed of 8 and 4 mm/s, respectively. These parameters generated the maximal printing resolution (line widths at about 300-400 µm). Core-shell construct in cell culture medium 1 hour after bioprinting (right).

The constructs were incubated for 4 days to allow proliferation before switching to differentiation medium (DM), composed of 10% Horse Serum, 1% Penicillin-Streptomycin, 200 mM L-Glutamine, 50 ng/mL IGF-1, 1 mg/mL ACA, and 78% DMEM (high glucose, pyruvate). The DM was selected following the protocol described by Guix et al. (2021), demonstrating the successful differentiation of myoblasts into functional myofibers. ACA was included to reduce hydrogel degradation by proteases, while IGF-1 facilitated myoblast fusion into myotubes and promoted compaction of the muscle actuator. Additionally, L-Glutamine enhanced differentiation and mitigated myotube atrophy. The constructs were maintained in DM for 7 days to support differentiation, with media changes every two days. Passive mechanical training occurred naturally due to hydrogel contraction during the differentiation process, further preparing the MTUs for subsequent analysis.

To optimize the maturation template's design for the most preservative and stable interaction with the tissue, we tested different designs of insertion mechanisms in the tendon region of the constructs (**Fig. S21**). We used pillar-anchoring systems with variable numbers and geometry of the pillars to find the most stabilized interface with the tissue. When comparing the 12-pillar systems with those with 9 pillars, we noticed that the 9-pillar configuration provided significantly better integration of MTUs. The 12-pillar structures exhibited poor integration, and in some cases, even structural damage. Additionally, cylindrical pillars were more effective at supporting MTU integration than conical pillars, which frequently led to MTU detachment during maturation. Overall, manually deposited MTUs successfully integrated with the anchoring system utilizing the 9-pillar configuration and cylindrical pillar design.

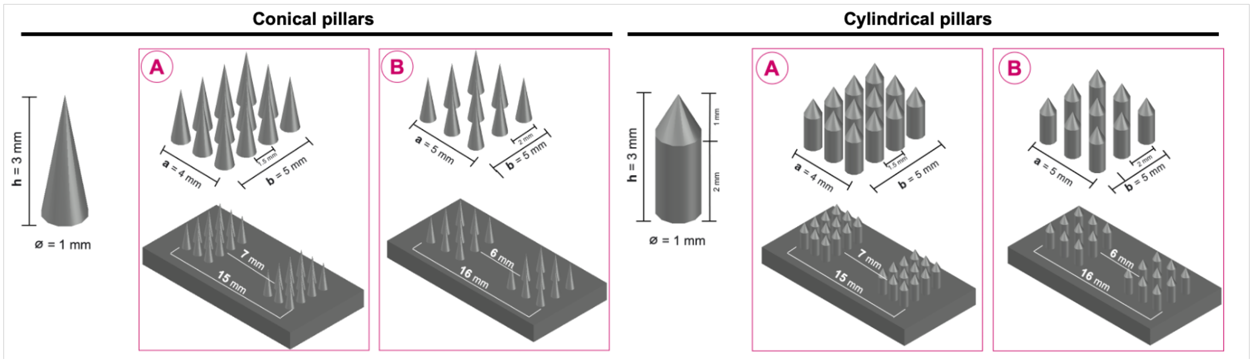

Fig. S21 | Design optimization of the tissue maturation templates.

We tested different configurations and geometries of pillars interfacing with the tendon-like regions of our constructs. Conical (left) or cylindrical (right) pillars in different numbers were tested, to evaluate if the shape and density of pillars, as well as the pillar-pillar distance, could have a role in stabilizing the bio-hybrid system.

Histological analysis showed the initiation of muscle tissue formation with myotubes forming in the bioactuator's area (**Fig. S22A**). The interface between the materials was solid and continuous, suggesting that the two bioinks can seamlessly integrate (**Fig. S22B**). Moreover, most fibroblasts migrated toward the external surface of the MTU, with few or no cells observed within the central tendinous region, as shown in **Fig. S22C**. The biphasic structure was also preserved in fragile areas, such as the insertion points of the pillars of the maturation templates (**Fig. S22D**).

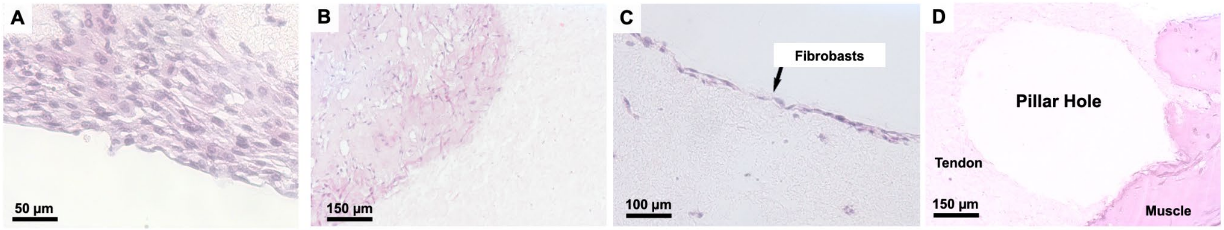

Fig. S22 | Tissue formation in core-shell MTU.

H&E staining on the tissue section showing a high-cell density tissue and early myotube formation (A), a cohesive interface created between the muscle and tendon-like tissue (B), fibroblasts aligned to the external surface of the MTU (C), and an intact interface in the area of tissue pinning to the maturation template with visible holes left from attachment to the pillars (D).

Despite this, the interface between the muscle and tendon sections was cohesive and well-established, enabling the two distinct hydrogels to endure passive forces generated by the mechanical training (**Figs. S22-S23**). In a few constructs, no muscle tissue was observed in the core, and only a small number of fibroblasts were observed surviving at the construct's borders (**Fig. S23**).

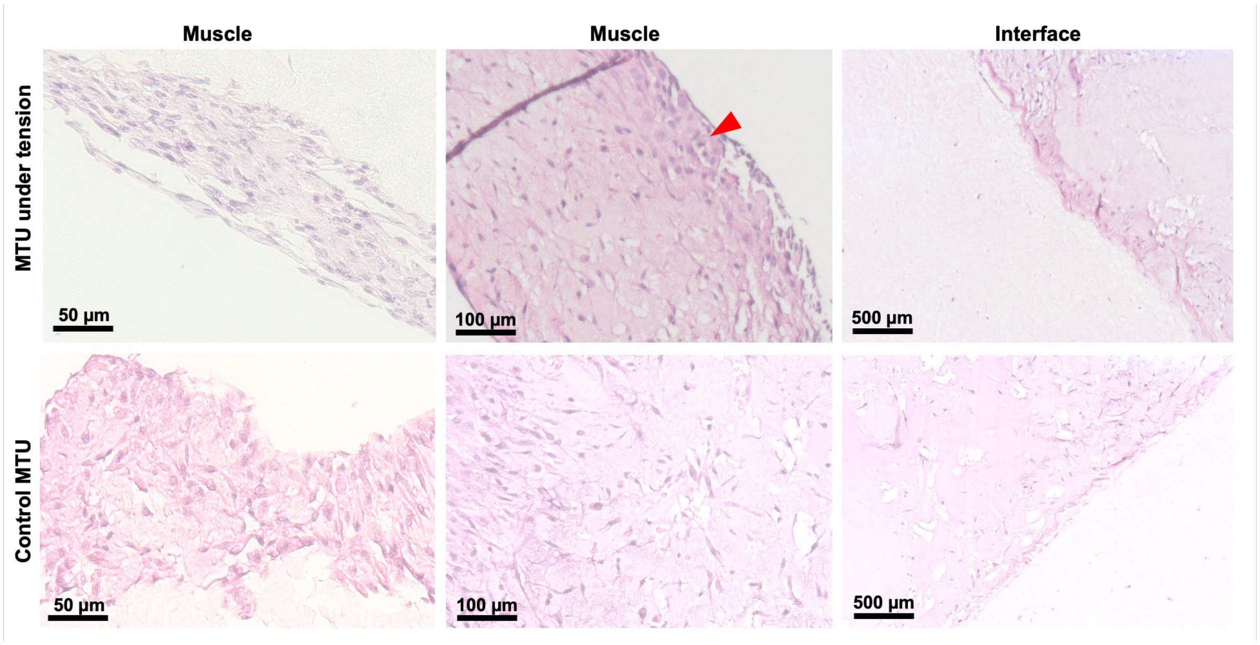

Fig. S23 | Tissue formation in core-shell MTUs with mechanical tensioning.

H&E staining on the tissue section showing aligned myotubes on the surface of MTU that were mechanically trained and more isotropic cell dispersion in the controls (left), myotube formation (red arrows) clustering to the surface of the tissue (middle), and the interface area where cells are present in the muscle zone and the tissue-tissue border but not in the tendon-like zone (right).

S14. Supplementary Movies

Movie S1. Comparative optical flow analysis.
Dynamic analysis performed on the MTU and control groups with optical flow analysis.

Movie S2. Motion in the anchors of the acellular tendon group.
On day 15 post-bioprinting, localized contractions were detected in the anchor area of the constructs printed without any cell in the anchor space ("acellular tendons"). In contrast, no localized movement was observed within the anchors of the fibroblast-seeded MTUs, which acted as rigid elements and passively followed the contraction of the bio-actuator.

Movie S3. Pulse pattern-synchronized motion of MTU.
The contraction frequency of the sparse MTU matched the electrical pulse pattern provided. The kinetic energy was calculated from recorded movies of the actuation experiments by segmenting the bioactuator area (internal region of the constructs).

Movie S4. Functional durability of the MTUs.
At 12 weeks after the biofabrication, the MTU responded to electrical stimulation by displaying vigorous contractions leading to the construct's displacement when left in an untethered pose (namely, removed from the constraints of the pillars).

Movie S5. Spontaneous contractility of the MTUs.
At 5 days after the biofabrication, the MTU displayed localized events of spontaneous muscle tissue contractions, which could sporadically be observed also after almost 4 weeks of tissue culture.

S15. Conclusions

In summary, we formulated muscle and tendon bioinks suitable for extrusion-based bioprinting and biofabricated functional MTUs composed of a fibroblast-myoblast co-culture. To ensure the stability of the interface between the two tissues, our design incorporated an interdigitated MTJ that mimics the native MTJ structure. Comparative analysis between the MTU and control systems revealed that including fibroblasts enhanced myogenic differentiation and prevented myoblast migration from the central area to the tendon components, contributing to stabilizing a heterocellular design across a tissue development of several weeks. The motile function expressed as contractile kinetic energy significantly varied only for more effective MTU designs that accounted for less bulky structures and larger muscle tissue area. Nevertheless, these designs featured lower myotube alignment, suggesting that further optimization of culture protocols is required to ensure uniform mechanical tension across the tissue. Our study contributed insights into understanding force production from different designs of bioactuators and, importantly, highlighted that due to the presence of tendons with intermediate stiffness between soft muscle tissue and printable PDMS, an effective energy transfer across the system elements occurred.

Our printed MTU showcased the potential of designing and realizing bioactuators with strong similarity to the native musculoskeletal system's mechanics. The printability of the muscle and tendon bioinks also made our approach easily adaptable for different geometries of printed constructs, amplifying the design space of MTUs and unlocking new possible forms of efficient robotic systems. Furthermore, the functional durability/lifetime, mechanical stability, and size (*i.e.,* centimeter-scale dimensions) of the printed MTU make it promising for use in replicating anatomically relevant structures. Designs aiming to maximize perfusability and tissue survival hold the promise to scale this system up allowing one to replicate even larger anatomic structures that incorporate musculoskeletal organization and energetic efficiency. From a wider perspective, our highlights to optimize MTU systems will contribute to realizing more biomimetic and functional 3D in vitro myotendinous models for biomedical applications in disease modeling, drug testing, and tissue regeneration studies. In conclusion, our work demonstrated a pivotal step towards engineering scalable, biomimetic muscle-tendon constructs that bridge the gap between tissue engineering and biohybrid robotics, providing a versatile, dynamic platform for advancing both robotic systems and cell culture models.

S16. Extended Description of the Authors' Contributions

M.F., D.M., R.K., and J.F. conceived the original idea; M.F., D.M., R.K. designed the study; M.F., D.M., and J.F. performed experiments relating to material characterization, cell biology, and biomolecular assays; performed the analysis; and wrote the manuscript. M.F., D.M. and A.Bal. performed Calcium imaging. M.F., P.P. and A.Bal. performed force measurements. D.M. and J.F. performed the bioprinting. M.M. performed DiffPD simulation of the MTU motion and optical flow analysis. R.H. performed microindentation tests. A.Bad. supported experimental realization in bioprinting. A.Bar. and S.E. performed design MTU optimization. M.F., D.M., J.F., and R.K. revised the manuscript. R.K. provided funding; M.F. and R.K. supervised the study. All authors approved the final draft of the manuscript.

REFERENCES AND NOTES

1. M. Filippi, T. Buchner, O. Yasa, S. Weirich, R. K. Katzschmann, Microfluidic tissue engineering and bio-actuation. *Adv. Mater.* **34**, e2108427 (2022).

2. L. Gao, M. U. Akhtar, F. Yang, S. Ahmad, J. He, Q. Lian, W. Cheng, J. Zhang, D. Li, Recent progress in engineering functional biohybrid robots actuated by living cells. *Acta Biomater.* **121**, 29–40 (2021).

3. L. Ricotti, B. Trimmer, A. W. Feinberg, R. Raman, K. K. Parker, R. Bashir, M. Sitti, S. Martel, P. Dario, A. Menciassi, Biohybrid actuators for robotics: A review of devices actuated by living cells. *Sci. Rob.* **2**, eaaq0495 (2017).

4. M. Filippi, O. Yasa, R. D. Kamm, R. Raman, R. K. Katzschmann, Will microfluidics enable functionally integrated biohybrid robots? *Proc. Natl. Acad. Sci. U.S.A.* **119**, e2200741119 (2022).

5. S. Yin, X. Zhang, C. Zhan, J. Wu, J. Xu, J. Cheung, Measuring single cardiac myocyte contractile force via moving a magnetic bead. *Biophys. J.* **88**, 1489–1495 (2005).

6. H. Fujita, V. T. Dau, K. Shimizu, R. Hatsuda, S. Sugiyama, E. Nagamori, Designing of a Si-MEMS device with an integrated skeletal muscle cell-based bio-actuator. *Biomed. Microdevices* **13**, 123–129 (2011).

7. T. Morita, M. Nie, S. Takeuchi, Human induced pluripotent stem cell-derived cardiac muscle rings for biohybrid self-beating actuator. *Lab Chip* **24**, 3377–3387 (2024).

8. R. Raman, C. Cvetkovic, S. G. M. Uzel, R. J. Platt, P. Sengupta, R. D. Kamm, R. Bashir, Optogenetic skeletal muscle-powered adaptive biological machines. *Proc. Natl. Acad. Sci. U.S.A.* **113**, 3497–3502 (2016).

9. G. J. Pagan-Diaz, X. Zhang, L. Grant, Y. Kim, O. Aydin, C. Cvetkovic, E. Ko, E. Solomon, J. Hollis, H. Kong, T. Saif, M. Gazzola, R. Bashir, Simulation and fabrication of stronger, larger, and faster walking biohybrid machines. *Adv. Funct. Mater.* **28**, 1801145 (2018).

10. B. Jo, K. Motoi, Y. Morimoto, S. Takeuchi, Dynamic and static workout of in vitro skeletal muscle tissue through a weight training device. *Adv. Healthc. Mater.* **13**, e2401844 (2024).

11. Y. Morimoto, H. Onoe, S. Takeuchi, Biohybrid robot powered by an antagonistic pair of skeletal muscle tissues. *Sci. Rob.* **3**, eaat4440 (2018).

12. K. A. Shama, M. A. Turner, H. B. Broadaway, E. L. Aikman, W. L. Stoppel, B. L. Taylor, Advances in tissue engineering approaches for repairing and rehabilitating the myotendinous junction. *Curr. Opin. Biomed. Eng.* **30**, 100532 (2024).

13. S. Tong, Y. Sun, B. Kuang, M. Wang, Z. Chen, W. Zhang, J. Chen, A comprehensive review of muscle–tendon junction: Structure, function, injury and repair. *Biomedicines* **12**, 423 (2024).

14. J. R. Jakobsen, M. R. Krogsgaard, The myotendinous junction—A vulnerable companion in sports. A narrative review. *Front. Physiol.* **12**, 635561 (2021).

15. F. Snow, C. O'Connell, P. Yang, M. Kita, E. Pirogova, R. J. Williams, R. M. I. Kapsa, A. Quigley, Engineering interfacial tissues: The myotendinous junction. *APL Bioeng.* **8**, 021505 (2024).

16. R. M. Duffy, A. W. Feinberg, Engineered skeletal muscle tissue for soft robotics: Fabrication strategies, current applications, and future challenges. *Wiley Interdiscip. Rev. Nanomed. Nanobiotechnol.* **6**, 178–195 (2014).

17. T. Nomura, M. Takeuchi, E. Kim, Q. Huang, Y. Hasegawa, T. Fukuda, Development of cultured muscles with tendon structures for modular bio-actuators. *Micromachines* **12**, 379 (2021).

18. N. Rao, S. Evans, D. Stewart, K. H. Spencer, F. Sheikh, E. E. Hui, K. L. Christman, Fibroblasts influence muscle progenitor differentiation and alignment in contact independent and dependent manners in organized co-culture devices. *Biomed. Microdevices* **15**, 161–169 (2013).

19. T. K. Merceron, M. Burt, Y.-J. Seol, H.-W. Kang, S. J. Lee, J. J. Yoo, A. Atala, A 3D bioprinted complex structure for engineering the muscle–tendon unit. *Biofabrication* **7**, 035003 (2015).

20. W. J. Kim, G. H. Kim, A bioprinted complex tissue model for myotendinous junction with biochemical and biophysical cues. *Bioeng. Transl. Med.* **7**, e10321 (2022).

21. R. Raman, C. Cvetkovic, R. Bashir, A modular approach to the design, fabrication, and characterization of muscle-powered biological machines. *Nat. Protoc.* **12**, 519–533 (2017).

22. R. Raman, L. Grant, Y. Seo, C. Cvetkovic, M. Gapinske, A. Palasz, H. Dabbous, H. Kong, P. P. Pinera, R. Bashir, Damage, healing, and remodeling in optogenetic skeletal muscle bioactuators. *Adv. Healthc. Mater.* **6**, 1700030 (2017).

23. S. Asim, T. A. Tabish, U. Liaqat, I. T. Ozbolat, M. Rizwan, Advances in gelatin bioinks to optimize bioprinted cell functions. *Adv. Healthc. Mater.* **12**, e2203148 (2023).

24. R. Derda, A. Laromaine, A. Mammoto, S. K. Y. Tang, T. Mammoto, D. E. Ingber, G. M. Whitesides, Paper-supported 3D cell culture for tissue-based bioassays. *Proc. Natl. Acad. Sci. U.S.A.* **106**, 18457–18462 (2009).

25. M. Filippi, O. Yasa, J. Giachino, R. Graf, A. Balciunaite, L. Stefani, R. K. Katzschmann, Perfusable biohybrid designs for bioprinted skeletal muscle tissue. *Adv. Healthc. Mater.* **12**, e2300151 (2023).

26. M. Filippi, T. Später, M. Herrmann, M. W. Laschke, A. Scherberich, S. Verrier, "Chapter 14—Strategies to promote vascularization, survival, and functionality of engineered tissues," in *Tissue Engineering*, J. De Boer, C. A. V. Blitterswijk, J. A. Uquillas, N. Malik, Eds. (Academic Press, 3rd ed. 2023), pp. 457–489; https://sciencedirect.com/science/article/pii/B9780128244593000147.

27. B. Goldberg, Collagen synthesis as a marker for cell type in mouse 3T3 lines. *Cell* **11**, 169–172 (1977).

28. N. Ito, Y. Miyagoe-Suzuki, S. Takeda, A. Kudo, Periostin is required for the maintenance of muscle fibers during muscle regeneration. *Int. J. Mol. Sci.* **22**, 3627 (2021).

29. P. Muñoz-Cánoves, C. Scheele, B. K. Pedersen, A. L. Serrano, Interleukin-6 myokine signaling in skeletal muscle: A double-edged sword? *FEBS J.* **280**, 4131–4148 (2013).

30. M. Hoene, H. Runge, H. U. Häring, E. D. Schleicher, C. Weigert, Interleukin-6 promotes myogenic differentiation of mouse skeletal muscle cells: Role of the STAT3 pathway. *Am. J. Physiol. Cell Physiol.* **304**, C128–C136 (2013).

31. P. J. Steyn, K. Dzobo, R. I. Smith, K. H. Myburgh, Interleukin-6 induces myogenic differentiation via JAK2-STAT3 signaling in mouse C2C12 myoblast cell line and primary human myoblasts. *Int. J. Mol. Sci.* **20**, 5273 (2019).

32. A. L. Serrano, B. Baeza-Raja, E. Perdiguero, M. Jardí, P. Muñoz-Cánoves, Interleukin-6 is an essential regulator of satellite cell-mediated skeletal muscle hypertrophy. *Cell Metab.* **7**, 33–44 (2008).

33. J. E. Belizário, C. C. Fontes-Oliveira, J. P. Borges, J. A. Kashiabara, E. Vannier, Skeletal muscle wasting and renewal: A pivotal role of myokine IL-6. *SpringerPlus* **5**, 619 (2016).

34. Y. Li, J. Zhao, Y. Yin, K. Li, C. Zhang, Y. Zheng, The role of IL-6 in fibrotic diseases: Molecular and cellular mechanisms. *Int. J. Biol. Sci.* **18**, 5405–5414 (2022).

35. S. Ray, X. Ju, H. Sun, C. C. Finnerty, D. N. Herndon, A. R. Brasier, The IL-6 trans-signaling-STAT3 pathway mediates ECM and cellular proliferation in fibroblasts from hypertrophic scar. *J. Invest. Dermatol.* **133**, 1212–1220 (2013).

36. F. N. Kenny, S. Marcotti, D. B. De Freitas, E. M. Drudi, V. Leech, R. E. Bell, J. Easton, M.-C. Díaz-de-la-Loza, R. Fleck, L. Allison, C. Philippeos, A. Manhart, T. J. Shaw, B. M. Stramer, Autocrine IL-6 drives cell and extracellular matrix anisotropy in scar fibroblasts. *Matrix Biol.* **123**, 1–16 (2023).

37. W. Lin, H. Song, J. Shen, J. Wang, Y. Yang, Y. Yang, J. Cao, L. Xue, F. Zhao, T. Xiao, R. Lin, Functional role of skeletal muscle-derived interleukin-6 and its effects on lipid metabolism. *Front. Physiol.* **14**, 1110926 (2023).

38. S. Docherty, R. Harley, J. J. McAuley, L. A. N. Crowe, C. Pedret, P. D. Kirwan, S. Siebert, N. L. Millar, The effect of exercise on cytokines: Implications for musculoskeletal health: A narrative review. *BMC Sports Sci. Med. Rehabil.* **14**, 5 (2022).

39. I. H. Jonsdottir, P. Schjerling, K. Ostrowski, S. Asp, E. A. Richter, B. K. Pedersen, Muscle contractions induce interleukin-6 mRNA production in rat skeletal muscles. *J. Physiol.* **528**, 157–163 (2000).

40. C. Zhang, Y. Li, Y. Wu, L. Wang, X. Wang, J. Du, Interleukin-6/Signal Transducer and Activator of Transcription 3 (STAT3) pathway is essential for macrophage infiltration and myoblast proliferation during muscle regeneration. *J. Biol. Chem.* **288**, 1489–1499 (2013).

41. E. Wada, J. Tanihata, A. Iwamura, S. Takeda, Y. K. Hayashi, R. Matsuda, Treatment with the anti-IL-6 receptor antibody attenuates muscular dystrophy via promoting skeletal muscle regeneration in dystrophin-/utrophin-deficient mice. *Skelet. Muscle* **7**, 23 (2017).

42. N. R. Richbourg, M. K. Rausch, N. A. Peppas, Cross-evaluation of stiffness measurement methods for hydrogels. *Polymer* **258**, 125316 (2022).

43. F. Prabowo, A. L. Wing-Keung, H. H. Shen, Effect of curing temperature and cross-linker to pre-polymer ratio on the viscoelastic properties of a PDMS elastomer. *Adv. Mater. Res.* **1112**, 410–413 (2015).

44. Y. Yu, D. Sanchez, N. Lu, Work of adhesion/separation between soft elastomers of different mixing ratios. *J. Mater. Res.* **30**, 2702–2712 (2015).

45. F. A. Kiani, S. Fischer, Comparing the catalytic strategy of ATP hydrolysis in biomolecular motors. *Phys. Chem. Chem. Phys.* **18**, 20219–20233 (2016).

46. S. Assenza, A. S. Sassi, R. Kellner, B. Schuler, P. De Los Rios, A. Barducci, Efficient conversion of chemical energy into mechanical work by Hsp70 chaperones. *eLife* **8**, e48491 (2019).

47. L. Sun, Y. Yu, Z. Chen, F. Bian, F. Ye, L. Sun, Y. Zhao, Biohybrid robotics with living cell actuation. *Chem. Soc. Rev.* **49**, 4043–4069 (2020).

48. M. Costantini, C. Colosi, W. Święszkowski, A. Barbetta, Co-axial wet-spinning in 3D bioprinting: State of the art and future perspective of microfluidic integration. *Biofabrication* **11**, 012001 (2019).

49. M. Volpi, A. Paradiso, E. Walejewska, C. Gargioli, M. Costantini, W. Swieszkowski, Automated microfluidics-assisted hydrogel-based wet-spinning for the biofabrication of biomimetic engineered myotendinous junction. *Adv. Healthc. Mater.* **288**, 2402075 (2024).

50. S. Laternser, H. Keller, O. Leupin, M. Rausch, U. Graf-Hausner, M. Rimann, A novel microplate 3D bioprinting platform for the engineering of muscle and tendon tissues. *SLAS Technol.* **23**, 599–613 (2018).

51. M. G. P. Stoker, H. Rubin, Density dependent inhibition of cell growth in culture. *Nature* **215**, 171–172 (1967).

52. X. Trepat, J. J. Fredberg, Plithotaxis and emergent dynamics in collective cellular migration. *Trends Cell Biol.* **21**, 638–646 (2011).

53. D. T. Tambe, C. Corey Hardin, T. E. Angelini, K. Rajendran, C. Y. Park, X. Serra-Picamal, E. H. Zhou, M. H. Zaman, J. P. Butler, D. A. Weitz, J. J. Fredberg, X. Trepat, Collective cell guidance by cooperative intercellular forces. *Nat. Mater.* **10**, 469–475 (2011).

54. F. Nakamura, The role of mechanotransduction in contact inhibition of locomotion and proliferation. *Int. J. Mol. Sci.* **25**, 2135 (2024).

55. C. D. Paul, W.-C. Hung, D. Wirtz, K. Konstantopoulos, Engineered models of confined cell migration. *Annu. Rev. Biomed. Eng.* **18**, 159–180 (2016).

56. M. Filippi, F. Garello, O. Yasa, J. Kasamkattil, A. Scherberich, R. K. Katzschmann, Engineered magnetic nanocomposites to modulate cellular function. *Small* **18**, e2104079 (2022).

57. M. T. Doolin, K. M. Stroka, Physical confinement alters cytoskeletal contributions towards human mesenchymal stem cell migration. *Cytoskeleton* **75**, 103–117 (2018).

58. D. Pally, A. Naba, Extracellular matrix dynamics: A key regulator of cell migration across length-scales and systems. *Curr. Opin. Cell Biol.* **86**, 102309 (2024).

59. M. S. Hall, J. T. Decker, L. D. Shea, Towards systems tissue engineering: Elucidating the dynamics, spatial coordination, and individual cells driving emergent behaviors. *Biomaterials* **255**, 120189 (2020).

60. M. A. Skylar-Scott, J. Y. Huang, A. Lu, A. H. M. Ng, T. Duenki, S. Liu, L. L. Nam, S. Damaraju, G. M. Church, J. A. Lewis, Orthogonally induced differentiation of stem cells for the programmatic patterning of vascularized organoids and bioprinted tissues. *Nat. Biomed. Eng.* **6**, 449–462 (2022).

61. A. Sanchez-Rubio, V. Jayawarna, E. Maxwell, M. J. Dalby, M. Salmeron-Sanchez, Keeping it organized: Multicompartment constructs to mimic tissue heterogeneity. *Adv. Healthc. Mater.* **12**, e2202110 (2023).

62. S. Sittadjody, J. M. Saul, E. C. Opara, Compartmentalization of two cell types in multilayered alginate microcapsules. *Methods Mol. Biol.* **1479**, 225–235 (2017).

63. S. Kriegman, D. Blackiston, M. Levin, J. Bongard, A scalable pipeline for designing reconfigurable organisms. *Proc. Natl. Acad. Sci. U.S.A.* **117**, 1853–1859 (2020).

64. C. Vesga-Castro, J. Aldazabal, A. Vallejo-Illarramendi, J. Paredes, Contractile force assessment methods for in vitro skeletal muscle tissues. *eLife* **11**, e77204 (2022).

65. L. Terrie, M. Burattini, S. Van Vlierberghe, L. Fassina, L. Thorrez, Enhancing myoblast fusion and myotube diameter in human 3D skeletal muscle constructs by electromagnetic stimulation. *Front. Bioeng. Biotechnol.* **10**, 892287 (2022).

66. Z. Ren, E. H. Ahn, M. Do, D. B. Mair, A. Monemianesfahani, P. H. U. Lee, D.-H. Kim, Simulated microgravity attenuates myogenesis and contractile function of 3D engineered skeletal muscle tissues. *NPJ Microgravity* **10**, 18 (2024).

67. R. Hashiguchi, H. Ichikawa, M. Kumeta, D. Koyama, Control of myotube orientation using ultrasonication. *Sci. Rep.* **14**, 25737 (2024).

68. A. Jiao, C. T. Moerk, N. Penland, M. Perla, J. Kim, A. S. T. Smith, C. E. Murry, D.-H. Kim, Regulation of skeletal myotube formation and alignment by nanotopographically controlled cell-secreted extracellular matrix. *J. Biomed. Mater. Res. A* **106**, 1543–1551 (2018).

69. H. Kim, M.-C. Kim, H. H. Asada, Extracellular matrix remodelling induced by alternating electrical and mechanical stimulations increases the contraction of engineered skeletal muscle tissues. *Sci. Rep.* **9**, 2732 (2019).

70. H. Xu, T. Liang, L. Wei, J.-C. Zhu, X. Liu, C.-C. Ji, B. Liu, Z.-P. Luo, Nano-elastic modulus of tendon measured directly in living mice. *J. Biomech.* **116**, 110248 (2021).

71. I. Kurtaliaj, M. Golman, A. C. Abraham, S. Thomopoulos, Biomechanical testing of murine tendons. *J. Vis. Exp.* 10.3791/60280 (2019).

72. V. Burgio, M. Civera, M. Rodriguez Reinoso, E. Pizzolante, S. Prezioso, A. Bertuglia, C. Surace, Mechanical properties of animal tendons: A review and comparative study for the identification of the most suitable human tendon surrogates. *Processes* **10**, 485 (2022).

73. S. E. Brashear, R. P. Wohlgemuth, G. Gonzalez, L. R. Smith, Passive stiffness of fibrotic skeletal muscle in mdx mice relates to collagen architecture. *J. Physiol.* **599**, 943–962 (2021).

74. M. Beldjilali-Labro, A. Garcia Garcia, F. Farhat, F. Bedoui, J.-F. Grosset, M. Dufresne, C. Legallais, Biomaterials in tendon and skeletal muscle tissue engineering: Current trends and challenges. *Materials* **11**, 1116 (2018).

75. A. Herchenhan, M. L. Bayer, R. B. Svensson, S. P. Magnusson, M. Kjær, In vitro tendon tissue development from human fibroblasts demonstrates collagen fibril diameter growth associated with a rise in mechanical strength. *Dev. Dyn.* **242**, 2–8 (2013).

76. J. T. Shearn, N. Juncosa-Melvin, G. P. Boivin, M. T. Galloway, W. Goodwin, C. Gooch, M. G. Dunn, D. L. Butler, Mechanical stimulation of tendon tissue engineered constructs: Effects on construct stiffness, repair biomechanics, and their correlation. *J. Biomech. Eng.* **129**, 848–854 (2007).

77. L. Thorrez, K. DiSano, J. Shansky, H. Vandenburgh, Engineering of human skeletal muscle with an autologous deposited extracellular matrix. *Front. Physiol.* **9**, (2018).

78. H. Duong, B. Wu, B. Tawil, Modulation of 3D fibrin matrix stiffness by intrinsic fibrinogen–thrombin compositions and by extrinsic cellular activity. *Tissue Eng. Part A* **15**, 1865–1876 (2009).

79. H. Kim, T. Osaki, R. D. Kamm, H. H. Asada, Tri-culture of spatially organizing human skeletal muscle cells, endothelial cells, and fibroblasts enhances contractile force and vascular perfusion of skeletal muscle tissues. *FASEB J.* **36**, e22453 (2022).

80. H. Ma, C. Yang, Z. Ma, X. Wei, M. R. Younis, H. Wang, W. Li, Z. Wang, W. Wang, Y. Luo, P. Huang, J. Wang, Multiscale hierarchical architecture-based bioactive scaffolds for versatile tissue engineering. *Adv. Healthc. Mater.* **11**, e2102837 (2022).

81. S. F. Levinson, M. Shinagawa, T. Sato, Sonoelastic determination of human skeletal muscle elasticity. *J. Biomech.* **28**, 1145–1154 (1995).

82. G. A. Johnson, D. M. Tramaglini, R. E. Levine, K. Ohno, N.-Y. Choi, S. L.-Y. Woo, Tensile and viscoelastic properties of human patellar tendon. *J. Orthop. Res.* **12**, 796–803 (1994).

83. C. N. Maganaris, J. P. Paul, In vivo human tendon mechanical properties. *J. Physiol.* **521**, 307–313 (1999).

84. A. J. Engler, M. A. Griffin, S. Sen, C. G. Bönnemann, H. L. Sweeney, D. E. Discher, Myotubes differentiate optimally on substrates with tissue-like stiffness: Pathological implications for soft or stiff microenvironments. *J. Cell Biol.* **166**, 877–887 (2004).

85. R. Raman, Modeling muscle. *Science* **363**, 1051–1051 (2019).

86. Q.-V. Le, G. Shim, Biorobotic drug delivery for biomedical applications. *Molecules* **29**, 3663 (2024).

87. J. Troccaz, G. Dagnino, G.-Z. Yang, Frontiers of medical robotics: From concept to systems to clinical translation. *Annu. Rev. Biomed. Eng.* **21**, 193–218 (2019).

88. Y. Morimoto, H. Onoe, S. Takeuchi, Biohybrid device with antagonistic skeletal muscle tissue for measurement of contractile force. *Adv. Rob.* **33**, 1–11 (2019).

89. S. Rangarajan, L. Madden, N. Bursac, Use of flow, electrical, and mechanical stimulation to promote engineering of striated muscles. *Ann. Biomed. Eng.* **42**, 1391–1405 (2014).

90. A. Balciunaite, O. Yasa, M. Filippi, M. Y. Michelis, R. K. Katzschmann, "Bilayered biofabrication unlocks the potential of skeletal muscle for biohybrid soft robots," in *2024 IEEE 7th International Conference on Soft Robotics (RoboSoft)* (IEEE, 2024), pp. 525–530; https://ieeexplore.ieee.org/document/10522009.

91. R. Raman, Biofabrication of living actuators. *Annu. Rev. Biomed. Eng.* **26**, 223–245 (2024).

92. P. Won, S. H. Ko, C. Majidi, A. W. Feinberg, V. A. Webster-Wood, Biohybrid actuators for soft robotics: Challenges in scaling up. *Actuators* **9**, 96 (2020).

93. N. Iwasaki, M. Roldo, A. Karali, A. Sensini, G. Blunn, Development of muscle tendon junction in vitro using aligned electrospun PCL fibres. *Eng. Regen.* **5**, 409–420 (2024).

94. A. K. Miri, I. Mirzaee, S. Hassan, S. M. Oskui, D. Nieto, A. Khademhosseini, Y. S. Zhang, Effective bioprinting resolution in tissue model fabrication. *Lab Chip* **19**, 2019–2037 (2019).

95. O. Yasa, Y. Toshimitsu, M. Y. Michelis, L. S. Jones, M. Filippi, T. Buchner, R. K. Katzschmann, An overview of soft robotics. *Annu. Rev. Control Robot. Auton. Syst.* **6**, 1–29 (2023).

96. C.-H. Li, I.-H. Yang, C.-J. Ke, C.-Y. Chi, J. Matahum, C.-Y. Kuan, N. Celikkin, W. Swieszkowski, F.-H. Lin, The production of fat-containing cultured meat by stacking aligned

muscle layers and adipose layers formed from gelatin-soymilk scaffold. *Front. Bioeng. Biotechnol.* **10**, 875069 (2022).

97. D. Zoccolan, A. Giachetti, V. Torre, The use of optical flow to characterize muscle contraction. *J. Neurosci. Methods* **110**, 65–80 (2001).

98. B. K. P. Horn, B. G. Schunck, Determining optical flow. *Artif Intell* **17**, 185–203 (1981).

99. B. D. Lucas, T. Kanade, “An iterative image registration technique with an application to stereo vision,” in *IJCAI'81: Proceedings of the 7th International Joint Conference on Artificial Intelligence* (Morgan Kaufmann Publishers Inc., 1981), pp. 674–679.

100. T. Du, K. Wu, P. Ma, S. Wah, A. Spielberg, D. Rus, W. Matusik, DiffPD: Differentiable projective dynamics. *ACM Trans. Graph.* **41**, 13 (2022).

101. F. Raza, J. Su, J. Zhong, M. Qiu, “Recent advancement of gelatin for tissue engineering applications,” in *Interaction of Nanomaterials With Living Cells*, F. A. Sheikh, S. Majeed, M. A. Beigh, Eds. (Springer Nature, 2023), pp. 821–837; https://doi.org/10.1007/978-981-99-2119-5_27.

102. L. T. Denes, L. A. Riley, J. R. Mijares, J. D. Arboleda, K. McKee, K. A. Esser, E. T. Wang, Culturing C2C12 myotubes on micromolded gelatin hydrogels accelerates myotube maturation. *Skelet. Muscle* **9**, 17 (2019).

103. M. C. Echave, L. Saenz del Burgo, J. L. Pedraz, G. Orive, Gelatin as biomaterial for tissue engineering. *Curr. Pharm. Des.* **23**, 3567–3584 (2017).

104. I. Lukin, I. Erezuma, L. Maeso, J. Zarate, M. F. Desimone, T. H. Al-Tel, A. Dolatshahi-Pirouz, G. Orive, Progress in gelatin as biomaterial for tissue engineering. *Pharmaceutics* **14**, 1177 (2022).

105. X. Wang, Q. Ao, X. Tian, J. Fan, H. Tong, W. Hou, S. Bai, Gelatin-based hydrogels for organ 3D bioprinting. *Polymers* **9**, 401 (2017).

106. S. Li, X. Dan, H. Chen, T. Li, B. Liu, Y. Ju, Y. Li, L. Lei, X. Fan, Developing fibrin-based biomaterials/scaffolds in tissue engineering. *Bioact. Mater.* **40**, 597–623 (2024).

107. R. Sanz-Horta, A. Matesanz, A. Gallardo, H. Reinecke, J. L. Jorcano, P. Acedo, D. Velasco, C. Elvira, Technological advances in fibrin for tissue engineering. *J. Tissue Eng.* **14**, 20417314231190288 (2023).

108. J. Y. Joo, M. L. Amin, T. Rajangam, S. S. A. An, Fibrinogen as a promising material for various biomedical applications. *Mol. Cell. Toxicol.* **11**, 1–9 (2015).

109. M. L. Terpstra, J. Li, A. Mensinga, M. de Ruijter, M. H. P. van Rijen, C. Androulidakis, C. Galiotis, I. Papantoniou, M. Matsusaki, J. Malda, R. Levato, Bioink with cartilage-derived extracellular matrix microfibers enables spatial control of vascular capillary formation in bioprinted constructs. *Biofabrication* **14**, 034104 (2022).

110. Y.-C. Huang, R. G. Dennis, L. Larkin, K. Baar, Rapid formation of functional muscle in vitro using fibrin gels. *J. Appl. Physiol.* **98**, 706–713 (2005).

111. S. Xu, L. Zhao, Y. Li, X. Gu, Z. Liu, X. Han, W. Li, W. Ma, Activating the healing process: Three-dimensional culture of stem cells in Matrigel for tissue repair. *BMC Biotechnol.* **24**, 36 (2024).

112. Z. Lin, T. Jiang, Y. Yang, Y. Hong, Y. Gao, H. Xie, Z. Luo, Matrigel-fibrinogen-thrombin hydrogels with high bioactivity for the fabrication of self-propelled in vitro muscular tissues. *Appl. Mater. Today* **39**, 102315 (2024).

113. A. Alave Reyes-Furrer, S. De Andrade, D. Bachmann, H. Jeker, M. Steinmann, N. Accart, A. Dunbar, M. Rausch, E. Bono, M. Rimann, H. Keller, Matrigel 3D bioprinting of contractile human skeletal muscle models recapitulating exercise and pharmacological responses. *Commun Biol.* **4**, 1183 (2021).

114. J. M. Lyles, W. Amin, C. L. Weill, Matrigel enhances myotube development in a serum-free defined medium. *Int. J. Dev. Neurosci.* **10**, 59–67 (1992).

115. E. A. Aisenbrey, W. L. Murphy, Synthetic alternatives to Matrigel. *Nat. Rev. Mater.* **5**, 539–551 (2020).

116. K. Yue, G. Trujillo-de Santiago, M. M. Alvarez, A. Tamayol, N. Annabi, A. Khademhosseini, Synthesis, properties, and biomedical applications of gelatin methacryloyl (GelMA) hydrogels. *Biomaterials* **73**, 254–271 (2015).

117. M. Filippi, G. Born, M. Chaaban, A. Scherberich, Natural polymeric scaffolds in bone regeneration. *Front. Bioeng. Biotechnol.* **8**, 474 (2020).

118. G. H. Yang, W. Kim, J. Kim, G. Kim, A skeleton muscle model using GelMA-based cell-aligned bioink processed with an electric-field assisted 3D/4D bioprinting. *Theranostics* **11**, 48–63 (2021).
